# CCAAT-displacement protein/cut homeobox transcription factor (CUX1) represses estrogen receptor-alpha (ER-α) in triple-negative breast cancer cells and can be antagonized by muscadine grape skin extract (MSKE)

**DOI:** 10.1371/journal.pone.0214844

**Published:** 2019-04-09

**Authors:** Liza J. Burton, Ohuod Hawsawi, Janae Sweeney, Nathan Bowen, Tamaro Hudson, Valerie Odero-Marah

**Affiliations:** 1 Center for Cancer Research and Therapeutic Development, Department of Biological Sciences, Clark Atlanta University, Atlanta, Georgia, United States of America; 2 Department of Medicine, Howard University, Washington, DC, United States of America; University of Alabama at Birmingham, UNITED STATES

## Abstract

Triple-Negative Breast Cancers (TNBCs) are the most difficult to treat subtype of breast cancer and are often associated with high nuclear expression of Snail and Cathepsin L (Cat L) protease. We have previously shown that Snail can increase Cat L expression/activity in prostate and breast cancer cells. This study investigated the role of CUX1 (a downstream substrate of Cat L) in TNBC. We showed that Cat L and CUX1 were highly expressed in TNBC patient tissue/cell lines, as compared to ER-positive samples, using cBioportal data and western blot/zymography analyses. Additionally, luciferase reporter and chromatin immunoprecipitation assays showed that CUX1 directly bound to estrogen receptor-alpha (ER-α) promoter in MDA-MB-468, a representative TNBC cell line, and that CUX1 siRNA could restore ER-α transcription and protein expression. Furthermore, Snail and CUX1 expression in various TNBC cell lines was inhibited by muscadine grape skin extract (MSKE, a natural grape product rich in anthocyanins) or Cat L inhibitor (Z-FY-CHO) leading to decreased cell invasion and migration. MSKE decreased cell viability and increased expression of apoptotic markers in MDA-MB-468 cells, with no effect on non-tumorigenic MCF10A cells. MSKE also decreased CUX1 binding to ER-α promoter and restored ER-α expression in TNBC cells, while both MSKE and CUX1 siRNA restored sensitivity to estradiol and 4-hydoxytamoxifen as shown by increased cell viability. Therefore, CUX1 activated by Snail-Cat L signaling may contribute to TNBC *via* ER-α repression, and may be a viable target for TNBC using natural products such as MSKE that targets cancer and not normal cells.

## Introduction

Multiple studies have confirmed that Triple Negative Breast Cancer (TNBC) occurs in a higher percentage of African American and Latino women leading to demographic and racial outcome disparities [1]. TNBC is an aggressive subtype with limited treatment options and very poor prognosis following progression; consequently, there is a major need to better understand the molecular basis of TNBC and to develop effective treatments for this aggressive type of breast cancer. Snail transcription factor, a protein overexpressed in TNBC [2], is associated with the epithelial mesenchymal transition (EMT), which is characterized by cells transforming from epithelial cells that are cuboidal and adherent to mesenchymal cells that are spindle-shaped and migratory [3]. Snail has been shown to suppress estrogen receptor-alpha (ER-α), a key regulatory molecule in mammary epithelial cell development, by direct binding to its promoter [4, 5]. Loss of ER-α is correlated with TNBC, poor prognosis, increased recurrence after treatment, and an elevated incidence of metastasis [6].

Cysteine proteases such as Cathepsin L (Cat L) acts extracellularly to increase the degradation of basement membranes and extracellular matrix, thereby promoting cell invasion and metastasis [7]. However, an intracellular role for Cat L that does not involve the lysosomes has been reported with data showing that Cat L functions in the regulation of cell cycle progression through its presence in the nucleus and its ability to proteolytically process the CUX1 transcription factor from the full-length p200 form to the p110 and p90 isoform [8–10]. CUX1 p110/90 isoforms have been shown to bind Snail promoter to increase it transcription and bind E-cadherin promoter to repress its transcription leading to increased EMT, tumor migration and invasion [11]. We have recently shown that Snail promotes its own transcription in prostate and breast cancer cells by upregulating nuclear Cat L expression and activity which subsequently increases CUX1 degradation to the p110 and p90 isoforms, further promoting EMT [12]. However, the role of CUX1 in TNBC has not been reported.

Over the years natural products have been shown to have chemopreventive effects in various cancers. Muscadine grape skin extract (MSKE) has shown its ability to inhibit prostate cancer cell growth and promote apoptosis *in vitro* without toxicity to normal prostate epithelial cell, in part by antagonizing ERK and PI3K signaling [13]. It can also revert EMT in prostate and breast cancer cells [14]. Presently, MSKE has completed Phase I and II clinical trials for the management of localized prostate cancer at John Hopkins University, and currently a new clinical trial is enrolling at Wake Forest University, to test its treatment for metastatic cancer [15]. However, MSKE has never been tested in breast cancer patients. In our current study we show for the first time that CUX1 is higher in TNBC and can negatively regulate the transcription and expression of ER-α by direct binding to its promoter, which is antagonized by MSKE, possibly allowing for sensitivity to tamoxifen.

## Materials and methods

### Cell culture, reagents and antibodies

The human breast cancer cells lines MCF10-A, MCF-7, and MDA-MB-468, and MDA-MB-231 were obtained from ATCC, Manassas, VA. Cell lines were authenticated using short tandem repeats (STR) analysis, and used up to 15 passages. The authenticated MCF-7 cells were stably transfected with empty Neo vector (MCF-7 Neo) or constitutively active Snail (MCF-7 Snail) and represent an EMT model as described previously [16]. Cells were grown in RPMI medium (VWR Int., West Chester, PA) supplemented with 10% fetal bovine serum (FBS, Hyclone, South Logan, UT) and 1% penicillin/streptomycin (VWR Int., West Chester, PA), at 37°C with 5% CO_2_ in a humidified incubator. Charcoal/dextran treated FBS (DCC-FBS) was from Hyclone, South Logan, UT. Anti-mouse α-tubulin antibody, 4-hydroxytamoxifen and 17β-estradiol were from Sigma-Aldrich, St Louis, MO. Rat monoclonal anti-Snail, rabbit monoclonal anti-progesterone receptor A/B, rabbit monoclonal anti-Her2/ErbB2 and horseradish peroxidase (HRP)-conjugated goat anti-rat antibodies, anti-Bax, -Bcl-2 and–Caspase-7 antibodies were from Cell Signaling Technology, Danvers, MA. Cat L-specific inhibitor (Z-FY-CHO) was purchased from R&D Systems (Minneapolis, MN). The HRP-conjugated donkey anti-goat, mouse monoclonal anti-ER-α and goat monoclonal anti-CUX1 were purchased from Santa Cruz Biotechnology (Santa Cruz, CA). HRP-conjugated sheep anti-mouse and sheep anti-rabbit secondary antibodies were purchased from Amersham Biosciences, Buckinghamshire, UK. Luminata Forte HRP chemiluminescence detection reagent was purchased from EMD Millipore (Billerica, MA). The protease inhibitor cocktail was from Roche Molecular Biochemicals, Indianapolis, IN. MSKE was a kind gift from Dr. Tamaro Hudson, Department of Medicine, Howard University, Washington, DC.

### Ethics approval and consent to participate

Breast tumors with matched normal tissues were obtained from Protein biotechnologies, Ramona, CA. Protein Biotechnologies Inc. provides pharmaceutical, biotechnology, government, and academic institutions with human clinical specimen derivatives. Tissues are obtained through a global network of participating medical centers that employ IRB approved protocols and strict ethical guidelines to ensure patient confidentiality and safety.

### Breast patient tissue lysates

Breast tumors with matched normal tissues were obtained from Protein biotechnologies, Ramona, CA. Identical procedures are used to prepare all patient samples. Specimens are flash frozen to -120°C within 5 min of removal to minimize autolysis, oxidation, and protein degradation. Tissue specimens are homogenized in modified RIPA buffer (PBS, pH 7.4, 1 mM EDTA, and protease inhibitors) to obtain the soluble proteins, and centrifuged to clarify. Patient information is shown in S1 Table.

### Analysis of Cat L and CUX1 using publicly available datasets

To analyze Cat L and CUX1 mRNA expression in breast cancer patients, we obtained data from METABRIC, Nature 2012 and Nat Commun 2016, by using www.cbioportal.org. More specifically, on the website homepage, we selected “Query”, then “Breast Cancer (METABRIC, Nature 2012 & Nat Commun 2016)” which contained 2,509 samples. We selected “Putative copy-number alterations from DNA copy” from Select Genomic Profiles, and “All Tumors (2509)” from Select Patient/Case Set, then entered the gene set “CTSL” for Cat L, or “CUX1”, then selected “Submit Query”. We selected “Plots”. Under Horizontal Axis, we then selected “Clinical Attribute”, and “3-Gene classifier subtype”, while the Vertical Axis was “Genetic Profile” showing the gene mRNA Expression. This generated the figures. The mRNA expression (RNA Seq RPKM, Reads Per Kilobase Million) values for CTSL and CUX1 from the samples included in the 3-Gene classifier subtype were downloaded from the cbioportal plot page described above and saved as CTSL.txt and CUX1.txt for statistical analyses. One-way ANOVA with post-hoc Tukey HSD (honestly significant difference) test calculations were performed with the R package multcomp. The .txt files and the results of the statistical analyses are included in the Supplementary_Table file (S2 Table).

### Luciferase assay

MDA-MB-468 cells were cultured in phenol red-free RPMI for 3 days prior to the assay to remove residual estrogen from the cells. Transient transfections were performed with 25 nM of control non-silencing ON-TARGET (Catalog #D-001810-10) or ON-TARGET plus CUX1 siRNA (Catalog #L-005841-00; Thermo Scientific—Dharmacon, Lafayette, CO) as per the manufacturer's instructions. Briefly, MDA-MB-468 cells were seeded at a density of 2X10^4^ cells/well on solid white polystyrene 96-well tissue culture plates (Fisher Scientific) in phenol red-free RPMI overnight, and then incubated with either non-silencing control or CUX1 siRNA (25 nM) in phenol red-free RPMI without FBS or antibiotics for 5 h; subsequently the media was replaced with 5% DCC-FBS in phenol red-free RPMI for an additional 24 h. After treatment, cells were transiently transfected with luciferase reporter linked to 3 estrogen response elements (EREs), a kind gift from Dr Wei Xu, University of Wisconsin-Madison, Madison, WI. This pGL4.32 vector (Promega) contains the *luc2P* gene that was modified to contain 3 tandem consensus EREs upstream of the minimal promoter (pGL4.3xER) [17]. Transfections were performed using lipofectamine transfection reagent, according to manufacturer’s instructions, for 48 h. Cell were then washed with PBS and lysed with 35 μL lysis buffer (100 mM K_2_HPO_4_, 0.2% triton X-100, pH 7.8). Luciferase activity was assessed with Dual-Glo Luciferase assay system from Promega (Madison, WI) according to the supplier's protocol.

### Quantitative real time-PCR (qPCR)

Total RNA was isolated by using an RNeasy Mini Kit (Qiagen, Valencia, CA). Gene expression was defined as the threshold cycle number (CT). Mean fold change in expression of the target genes were calculated using the comparative CT method (RU; 2-ΔCt). All data were normalized to the quantity of RNA input by Glyceraldehyde 3-phosphate dehydrogenase (GAPDH). The following primers were used; CUX1 Forward primer: 5’ TGAACGACCCCAACAATGTGG 3’ Reverse primer: 5’ GGCTTTTGCTGATACGCTCG 3’, ER-α Forward primer: 5’ TGGTCAGTGCCTTGTTGGATG 3’ Reverse primer: 5’ TGTCTTGCCAGGTTGGTCAGTAAG 3’.

### Western blot analysis

Cells were lysed in a modified RIPA buffer as described previously [18].

Supernatants were collected and quantified using a micro BCA assay (Promega, Madison, WI). 30 μg of cell lysate was resolved using 10% sodium dodecyl sulfate–polyacrylamide gel electrophoresis, followed by trans-blotting onto nitrocellulose membrane (Bio-Rad Laboratories, Hercules, CA). Membranes were incubated with appropriate primary and secondary antibody, followed by visualization using Luminata Forte ECL reagent. The membranes were stripped using Restore western blot stripping buffer (Pierce Biotechnology, Rockford, IL) prior to reprobing with a different antibody. For treatments, 70% confluent cells were serum-starved in phenol red-free serum-free RPMI containing penicillin/streptomycin for 24 h prior to treatment with Z-FY-CHO (1, 5, or 20 μm) or MSKE (5 or 20 μg/ml) in phenol-free serum-free RPMI containing 5% DCC-FBS for 3 days.

### Zymography

We utilized the cathepsin zymograpy technique as described previously [19]. Briefly, lysates were electrophoresed using 0.2% gelatin substrate (Scholar Chemistry, Rochester, NY), incubated in cathepsin-renaturing buffer (65 mM Tris buffer, pH 7.4 with 20% glycerol) followed by overnight incubation in pH 6 sodium phosphate assay buffer (0.1 M sodium phosphate buffer, 1 mM ethylenediaminetetraacetic acid, 2 mM dithiothreitol) at 37°C. The gel was stained with Coomassie blue stain (10% acetic acid, 25% isopropanol, 4.5% Coomassie Blue), destained (10% isopropanol and 10% acetic acid) and proteolytic activity visualized as cleared bands. The pH conditions used will show both Cat L and cathepsin S (Cat S) activity.

### Cell migration and invasion assays

We utilized Costar 24-well plates containing a polycarbonate filter insert with an 8-μm pore size, to coat with 4.46 μg/ μl rat tail collagen I (BD Bioscience, Bedford, MA) on the outside for 24 h at 4°C. 5X10^4^ cells were plated in the upper chamber containing RPMI supplemented with 0.1% fetal bovine serum (FBS), whereas the lower chamber contained RPMI supplemented with 10% FBS. After 5 h, cells that migrated to the bottom of the insert were fixed, stained with 0.05% crystal violet, and counted to obtain the relative migration. Invasion assays were performed similarly, but for 24 h, using BD Matrigel invasion chambers (8-μm pores Thermo Fisher Scientific, Waltham, MA, USA). Each experiment was done in triplicate, and the graphs represent an average of the 3 wells. All the experiments were repeated at least three times.

### Subcellular fractionation

Subcellular fractionations were performed per the manufacturer's instructions (Thermo Scientific, Waltham, MA, USA). Briefly, cells at 80–90% confluence were lysed in a series of buffers containing protease inhibitors (25X) with CERI (250 μl), CERII (11 μl), or NER (100 μl). Centrifugation steps were performed to obtain a non-nuclear fraction and an intact nuclear pellet, followed by further lysing to isolate the nuclear fraction. 30 μg of non-nuclear and nuclear fractions were utilized for Western blot analysis. Mouse anti-topoisomerase I (Santa Cruz Biotechnology Santa Cruz, CA) and rabbit anti-GAPDH antibodies (Cell Signaling Technology, Inc., Danvers, MA) were used to ensure the integrity of nuclear and cytoplasmic fractions, respectively. Rabbit anti-Calnexin (Santa Cruz Biotechnology Santa Cruz, CA) was utilized as a control to ensure that the nuclear fraction was not contaminated with endoplasmic reticulum.

### Immunofluorescence

5X10^3^ cells were plated into 16 well chamber slides (Bio-Tek, Nunc, Winooski, VT). For treatments, cells were either untreated, treated with Z-FY-CHO or MSKE for 72 h. Fixation was performed with methanol/ethanol 1:1 volume for 5 min, followed by washes with 1X PBS and blocking with protein blocking solution without serum (Dako, Camarillo, CA) for 10 min at room temp. Subsequently, slides were incubated with primary antibody at 1:50 or 1:100 dilutions in Dako antibody diluent solution for 1 h at room temp. Slides were washed with 1X TBS-T (Dako, Camarillo, CA), then incubated with secondary antibody in the dark for 1 h at room temp. Secondary antibodies used were anti-rabbit Oregon green 488, anti-mouse Alexa red 594 (Invitrogen, Carlsbad, CA) or anti-goat Texas red (Vector Laboratories, Burlingame, CA). Slides were washed with 1X TBS-T and double deionized water, prior to counterstaining with DAPI (1 μg/ml, Santa Cruz Biotechnology, Santa Cruz, CA). Slides were mounted using Fluorogel mounting medium (Electron Microscopy Sciences, Hatfield, PA). Fluorescence microscopy was performed using Zeiss microscope and Axiovision Rel 4.8 software.

### Cell viability (MTS) assay

MCF-10A and MDA-MB-468 cells were plated at a density of 2,000 cells per well in 96-well plates and allowed to attach overnight. Cells were then treated with ethanol control or 20 μg/mL MSKE, and viability was assessed daily using the CellTiter 96 Aqueous One Solution Cell Proliferation Assay according to the supplier's protocol. MDA-MB-468 cells were also transiently transfected with 25 nM CUX1 or Control siRNA; treated with 20 μg/mL MSKE, 20 μM Z-FY-CHO alone; or pretreated with 20 μg/mL MSKE, 20 μM Z-FY-CHO, 25 nM CUX1 or Control siRNA for 24 h, and then subjected to 0.01 μM 17β-estradiol or 1 μM 4-hydroxytamoxifen, followed by assessment of cell viability daily.

### Chromatin immunoprecipitation (ChIP) assay

MCF-7 Snail and MDA-MB-468 cells were used for ChIP analysis using the EZ-ChIP kit according to manufacturer’s instructions (EMD Millipore). Briefly, 2.5 X 10^6^ cells were cross-linked with formaldehyde for 10 min at 37°C, washed in ice cold PBS, unreacted formaldehyde was quenched with glycine, then washed with PBS and re-suspended in SDS buffer. Samples were sonicated to approximately 600 bps with Sonicator (Misonix Sonicator S-3000), diluted in dilution buffer with inhibitors and precleared with agarose G beads. The supernatant was used directly for immunoprecipitation with anti-CUX1 or goat-IgG (for negative control). The immunocomplexes were mixed with 120 μl of DNA coated agarose G beads followed by incubation overnight. Pellets were washed in a low salt wash buffer (X1), high salt wash buffer (xX1), LiCl wash buffer (X1) and TE buffer (X2). This was followed by elution of the protein/DNA complex and reversal of cross-linking with 5M NaCl overnight. The protein was then digested with proteinase K followed by DNA purification with elution buffer. 2 μl of the DNA eluates from the ChIP assay were added into a 96 well qRT-PCR plate for each corresponding sample. Subsequently, a master mix was made using promoter primers that recognize the ER-α promoter region where the consensus sequence of CUX1 (ATCAAT) could bind. QRT-PCR was then done using an I-cycler (Bio-Rad) to quantitate transcript levels by the SYBR Green method. Cycle threshold differences were then determined using an I-cycler (Bio-Rad) relative to input chromatin (chromatin initially used for the immunoprecipitation). Fold changes in transcript levels of ER-α gene were then calculated and the results were graphed. Samples were also resolved on an agarose gel. As another control, qRT-PCR was performed with primers for ER-α control (promoter region that did not contain the CUX1 consensus sequence). The following are the specific primer pairs: ER-α Promoter (Forward-5’-AATTCTCCAATTAACAGTGAC-3’; Reverse-5’-GAACATGTGAACATAAAAACT-3’), ER-α Control (Forward 5’-GGCCTCACACATCAGGATAAA-3’; Reverse 5’-ATATCCCACAGCCTTGTCTTG-3’).

### Statistical analysis

Data were analyzed by a paired student's t-test or ANOVA using GraphPad Prism software. For all experiments * means 0.05 > *p* value > 0.01, ** means 0.01 > *p* value > 0.001, and *** means *p* value < 0.001.

## Results

### TNBC cells have higher nuclear active Cat L and cleaved CUX1 levels as compared to ER-positive breast cancer cells

**Fig 1:**
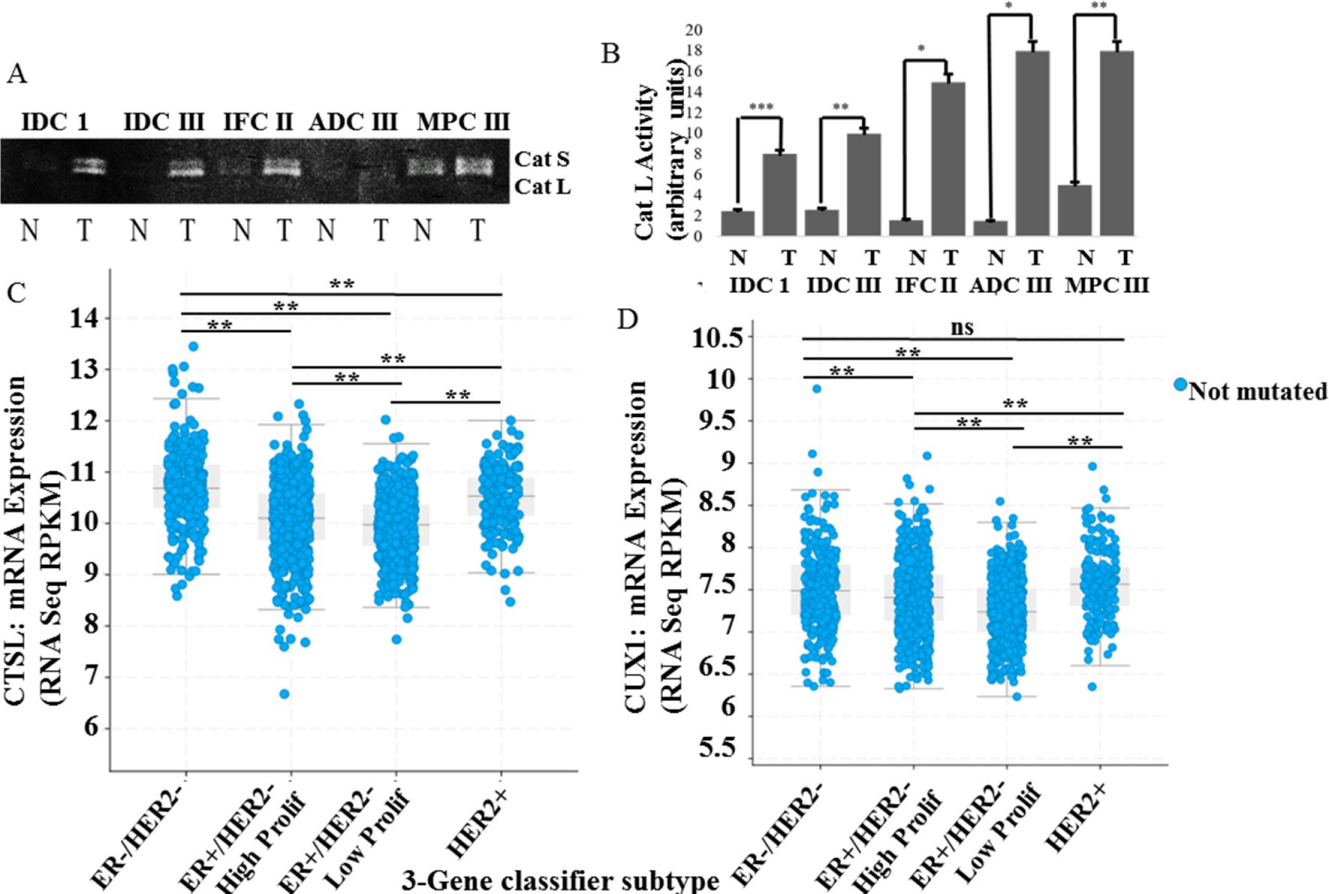
Increased Cat L activity/expression and CUX1 mRNA in high grade tumors and TNBC. (**A**) Cat L activity was analyzed by zymography in patient tumor lysates from invasive ductal carcinoma (IDC) grades 1 and 3, infiltrating carcinoma (IFC) grade 3, adenocarcinoma (grade 3) and metaplastic carcinoma (MPC) grade 3 as compared to normal matched control. (**B**) Densitometry was performed on zymograms. (C) Cat L (CTSL) and (D) CUX1 mRNA expression from METABRIC, Nature 2012 and Nat Commun 2016 was obtained using www.cbioportal.org. Graphical data represents at least three independent experiments * means 0.05 > *p* value > 0.01, ** means 0.01 > *p* value > 0.001, and *** means *p* value < 0.001.

To examine the difference in Cat L activity in patient lysates we used lysates prepared from normal/tumor-matched breast cancer patients (S1 Table for patient data) to perform zymography. We observed that patient tumor lysates expressed higher levels of active Cat L as compared to normal matched patient tissue (Fig 1A and 1B). Unfortunately, receptor status on these patient samples was not available from the pathology case reports to correlate to Cat L activity. Additionally, cbioportal, a publicly available database, revealed that Cat L and CUX1 mRNA levels were significantly higher in TNBC as compared to ER-positive tumors (Fig 1C and 1D).

**Fig 2:**
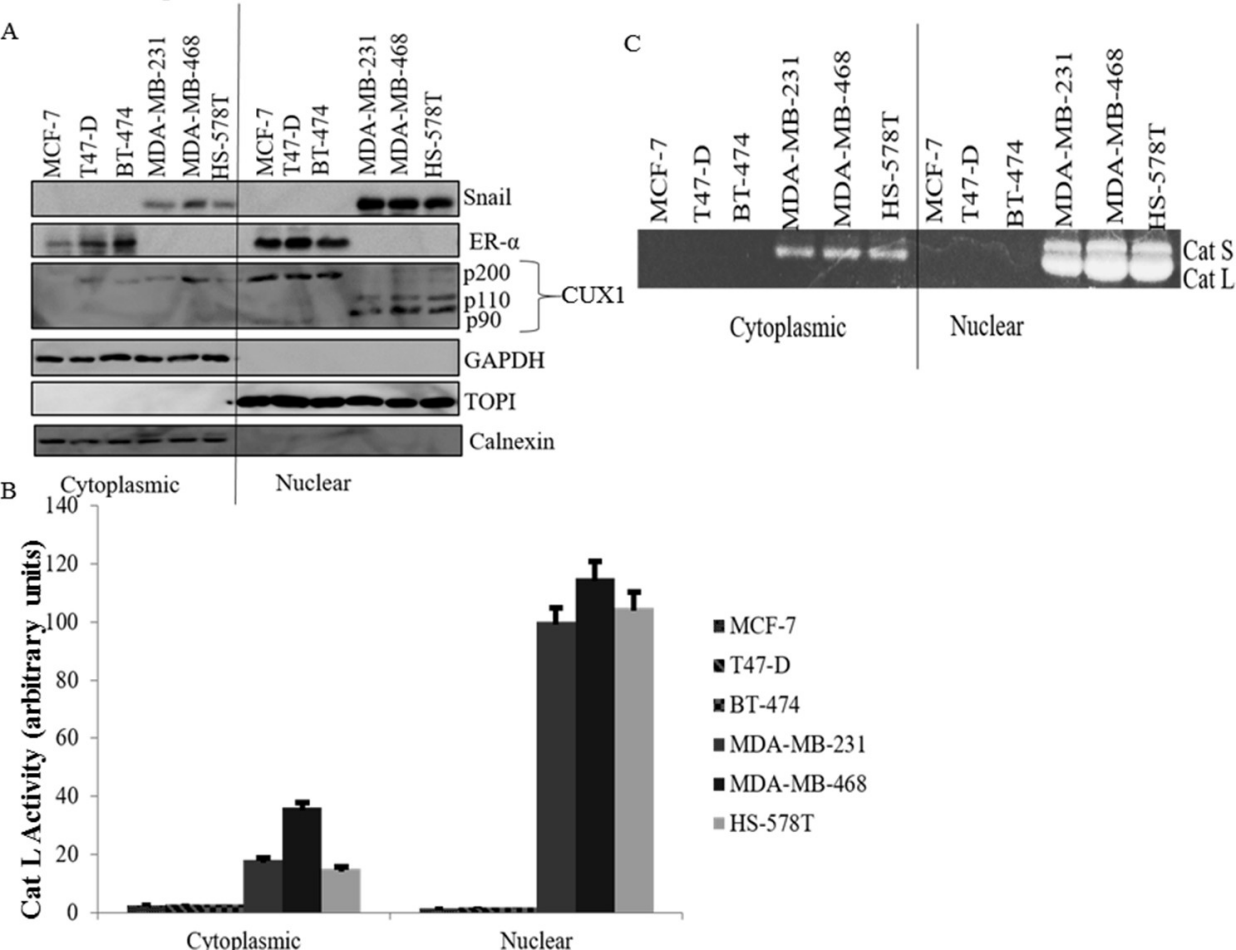
TNBC cells have higher levels of nuclear active Cat L and cleaved CUX1 as compared to ER-positive breast cancer cells. (**A**) CUX1 expression was analyzed by western blot in cytoplasmic and nuclear compartments of TNBC cells (MDA-MB-231, MDA-MB-468, HS-578T) compared to ER-positive cell lines (MCF-7, T47-D, BT-474). Blots were also probed with Snail and ER-α antibodies. GAPDH and topoisomerase I (TOPI) antibodies were utilized to confirm the integrity of the cytoplasmic and nuclear fractions, respectively, while calnexin antibody was included to ensure that the nuclear fraction was not contaminated with endoplasmic reticulum. (**B**) Zymography was also performed in these cytoplasmic and nuclear fractions of TNBC and ER-positive cells. (**C**) Densitometry of active Cat L is shown.

We then examined the expression of Snail, ER-α and CUX1 using subcellular fractionation of TNBC cells (MDA-MB-231, MDA-MB-468, and HS-578T) compared to ER-positive cells (MCF-7, T47-D, BT-474) followed by western blot analysis, as well as activity of Cat L by zymography. Western blot analysis showed TNBC cells have higher nuclear Snail expression as well as nuclear p110 and p90 cleavage products of CUX1 compared to ER-positive cells that have low Snail expression, express only the uncleaved p200 form of CUX1, as well as more nuclear ER-α (Fig 2A). We also found higher levels of active Cat L within nuclear compartment of TNBC cells as compared to ER-positive cells (Fig 2B and 2C). Immunofluorescence data confirmed western blot results and showed that TNBC cells have nuclear expression of Snail and CUX1 compared to ER-positive cells that display ER-α expression in the nucleus with undetectable expression of Snail or CUX1 (S1 Fig). This data shows that CUX1 and Cat L activity are higher in TNBC and have an inverse relationship with ER-α.

### CUX1 knockdown increases ER-α transcription and nuclear protein expression

**Fig 3:**
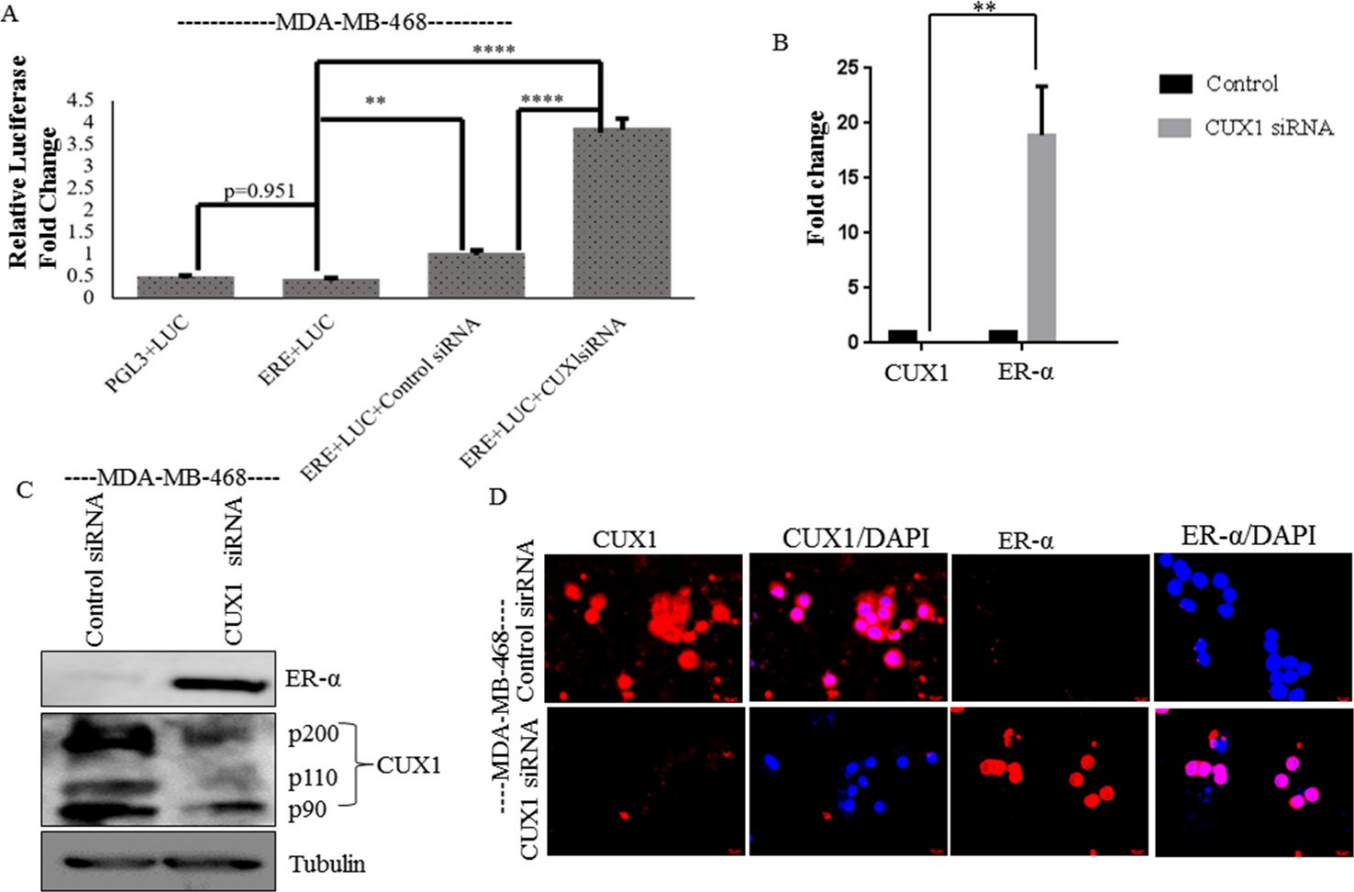
Knockdown of CUX1 restores ER-α expression in TNBC cells. (**A**) MDA-MB-468 cells were transiently transfected with luciferase reporter linked to 3 EREs (ERE+LUC) or empty vector (PGL3+LUC) or ERE+LUC with CUX1 siRNA or Control siRNA, followed by analysis of luciferase using Dual-Glo assay. All luciferase data were calculated using relative light units (RLU) on a luminometer. (**B**) qPCR and (**C**) Western blot analysis was performed on MDA-MB-468 cells transfected with CUX1 or Control siRNA and analyzed with CUX1 antibody to confirm CUX1 knockdown. ER-α expression was also analyzed in these cells. Tubulin was utilized as a loading control. (**D**) Immunofluorescence analysis shows ER-α is expressed in the nucleus of MDA-MB-468 cells when there is a knockdown of CUX1. The experiments were performed in triplicate at least three times independently. Graphical data represents three independent experiments ** means 0.01 > *p* value > 0.001, and **** means *p* value < 0.0001.

To further examine the possible relationship between ER-α and CUX1, we transiently knocked down CUX1 using siRNA in MDA-MB-468 cells and performed a luciferase assay using a luciferase reporter linked to 3 EREs. Transfection with CUX1 siRNA showed a significant increase in luciferase activity compared to control siRNA (Fig 3A). We performed qPCR, western blot analysis and immunofluorescence which confirmed that transient knockdown of CUX1 led to re-expression of ER-α compared to control siRNA (Fig 3B and 3C), and that it was localized in the nucleus (Fig 3D).

### CUX1 transcription factor directly represses ER-α and can be antagonized by Cat L inhibitor or MSKE

**Fig 4:**
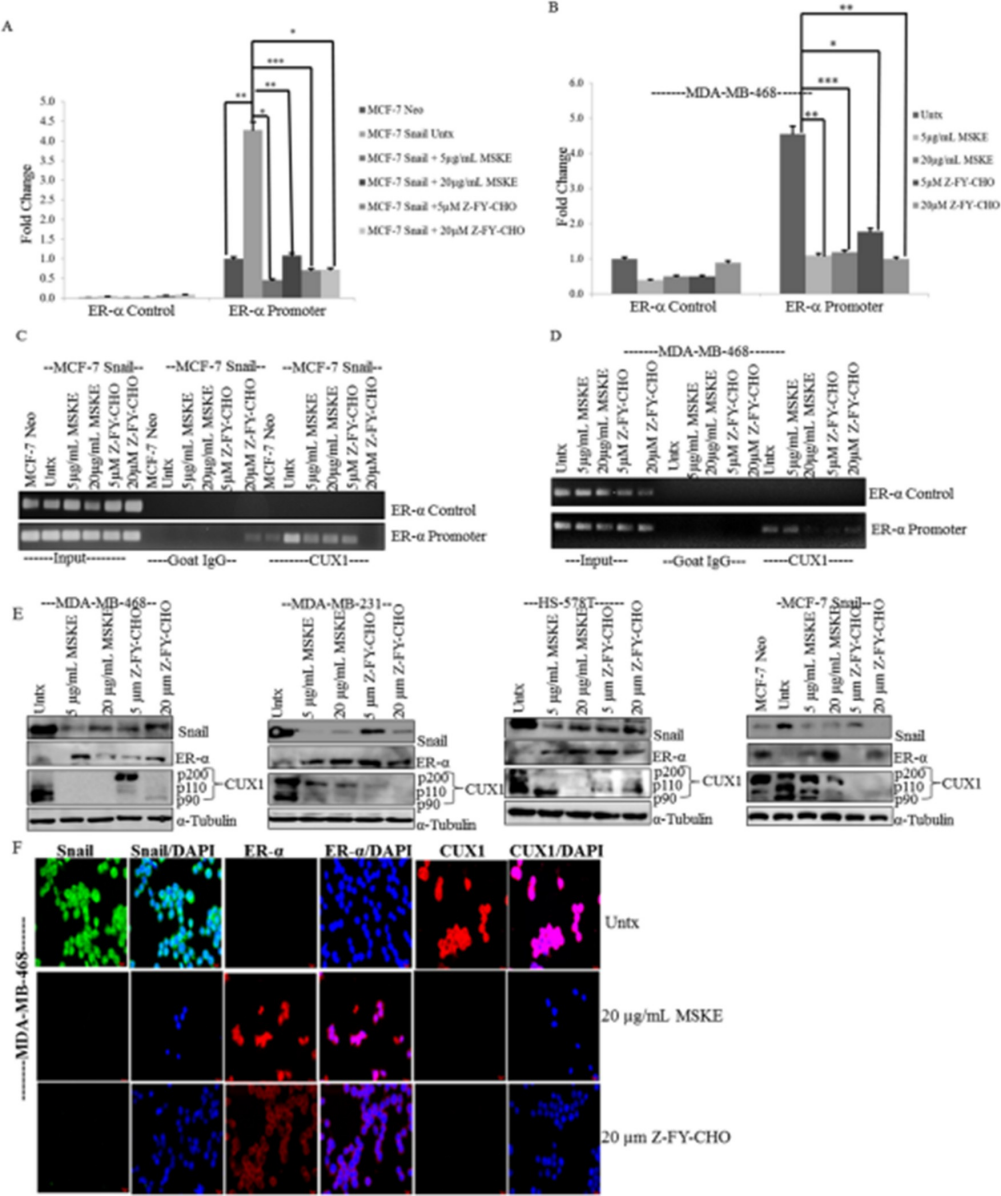
CUX1 directly binds to the ER-α promoter and is antagonized by Cat L inhibitor (Z-FY-CHO) or MSKE. (**A**) MCF-7 Neo and MCF-7 cells overexpressing Snail (MCF-7 Snail) or (**C**) MDA-MB-468 cells were utilized for ChIP analysis with anti-CUX1 antibody or goat IgG as a negative control to immunoprecipitate, and qRT-PCR with primers to ER-α promoter containing the CUX1 consensus site or primers to a promoter region that does not contain CUX1 consensus site (ER-α Control) as another control. Cells were also treated with Z-FY-CHO or MSKE prior to ChIP analysis which led to decreased CUX1 binding to the promoter. The results of the qRT-PCR were plotted as fold change of binding to ER-α promoter. (**B,D**). Results of qRT-PCR on agarose gel. MDA-MB-468, MDA-MB-231, HS-578T and MCF-7 Snail cells were treated with Z-FY-CHO (5, 20 μm) or MSKE (5, 20 μg/mL) for 72 h. (**E**) Western blot analysis shows treatments with MSKE and Z-FY-CHO led to decreased expression of Snail and CUX1 and increased ER-α. (**F**) Immunofluorescence analysis shows that in MDA-MB-468 cells treated with MSKE, ER-α is expressed in the nucleus while it is predominantly cytoplasmic in cells treated with Z-FY-CHO. The experiments were performed in triplicate at least three times independently. Graphical data represents three independent experiments * means 0.05 > *p* value > 0.01, ** means 0.01 > *p* value > 0.001, and *** means *p* value < 0.001.

CUX1 has been shown physically bind to the Snail or E-cadherin promoter to increase or decrease its transcription, respectively, thereby promoting EMT [11]. It has also been shown that Snail can promote its own transcription by increasing Cat L activity which then increases CUX1 proteolytic cleavage to generated p110 and p90 isoforms which subsequently bind to Snail promoter to increase its transcription [12]. Since it has been shown that Snail can bind ER-α promoter to repress ER-α [4, 5], it was plausible that CUX1may be repressing ER-α via Snail. However, we tested a novel hypothesis that CUX1 may also directly bind ER-α promoter. We also tested whether this could be antagonized with Z-FY-CHO (Cat L inhibitor which may prevent proteolytic cleavage of CUX1) or MSKE, a natural product that we have previously shown can inhibit Cat L activity [12]. Firstly, we had always alluded to MCF-7 cells overexpressing Snail as a possible TNBC model, and western blot analysis confirmed that Snail overexpression eliminates not just ER-α, but also PR and HER2 expression, showing that indeed it may represent a TNBC model (S2 Fig). Subsequently, a ChIP assay was performed using MDA-MB-468, MCF-7 Neo and Snail-transfected cells to immunoprecipitate CUX1 from chromatin and perform qRT-PCR with ER-α promoter primer that contains the CUX1 consensus sequence. Goat IgG and ER-α control primers (recognizes promoter region that does not contain CUX1 consensus site) were utilized as negative controls. The data revealed that CUX1 directly binds to ER-α promoter of MDA-MB-468 cells and more greatly in MCF-7 Snail cells as compared to MCF-7 Neo cells (Fig 4A–4D). Z-FY-CHO (5 and 20 μM) and MSKE (5 and 20 μg/mL) significantly decreased the binding of CUX1 to the ER-α promoter in both MCF-7 Snail and MDA-MB-468 cells (Fig 4A–4D). Next, we examined whether antagonizing CUX1 binding to ER-α promoter by MSKE would also restore ER-α protein expression as we had seen with CUX1 siRNA, and included Z-FY-CHO as a control. We treated three TNBC cells lines (MDA-MB-468, HS-578T, MDA-MB-231) and Snail overexpressing MCF-7 cells with 5 and 20 μg/mL MSKE or 5 and 20 μM Z-FY-CHO for 72 h, and found that the expression of Snail and CUX1 decreased with both treatments (Fig 4E), as we have previously seen [12]. Interestingly, ER-α was re-expressed in cells treated with Z-FY-CHO or MSKE (Fig 4E). For immunofluorescent analysis, we utilized MDA-MB-468 cells as a representative TNBC, which showed that treatments with 20 μM Z-FY-CHO and 20 μg/mL MSKE decreased Snail and CUX1 levels (Fig 4F). Although both treatments increased ER-α expression, Z-FY-CHO led to ER-α expression mainly in the cytoplasm, while MSKE led to re- expression of ER-α within the nucleus. We included MCF10A as a control and observed low levels of Snail and pro Cat L, while mature Cat L was negligible and did not change significantly with MSKE or Z-FY-CHO treatments (S3C Fig). ER- α expression in MCF10A cells was absent as has been previously reported [20, 21] and was unaffected by MSKE or Z-FY-CHO treatment (S3C Fig). Therefore, in control MCF10A cells, MSKE and Z-FY-CHO has no effect on the markers in this study. Thus, our data shows that binding of CUX1 to the ER-α promoter in TNBC cells can be antagonized by Z-FY-CHO or MSKE, which can also restore ER-α expression in TNBC cells.

### MSKE decreases cell migration and invasion in TNBC cells

**Fig 5:**
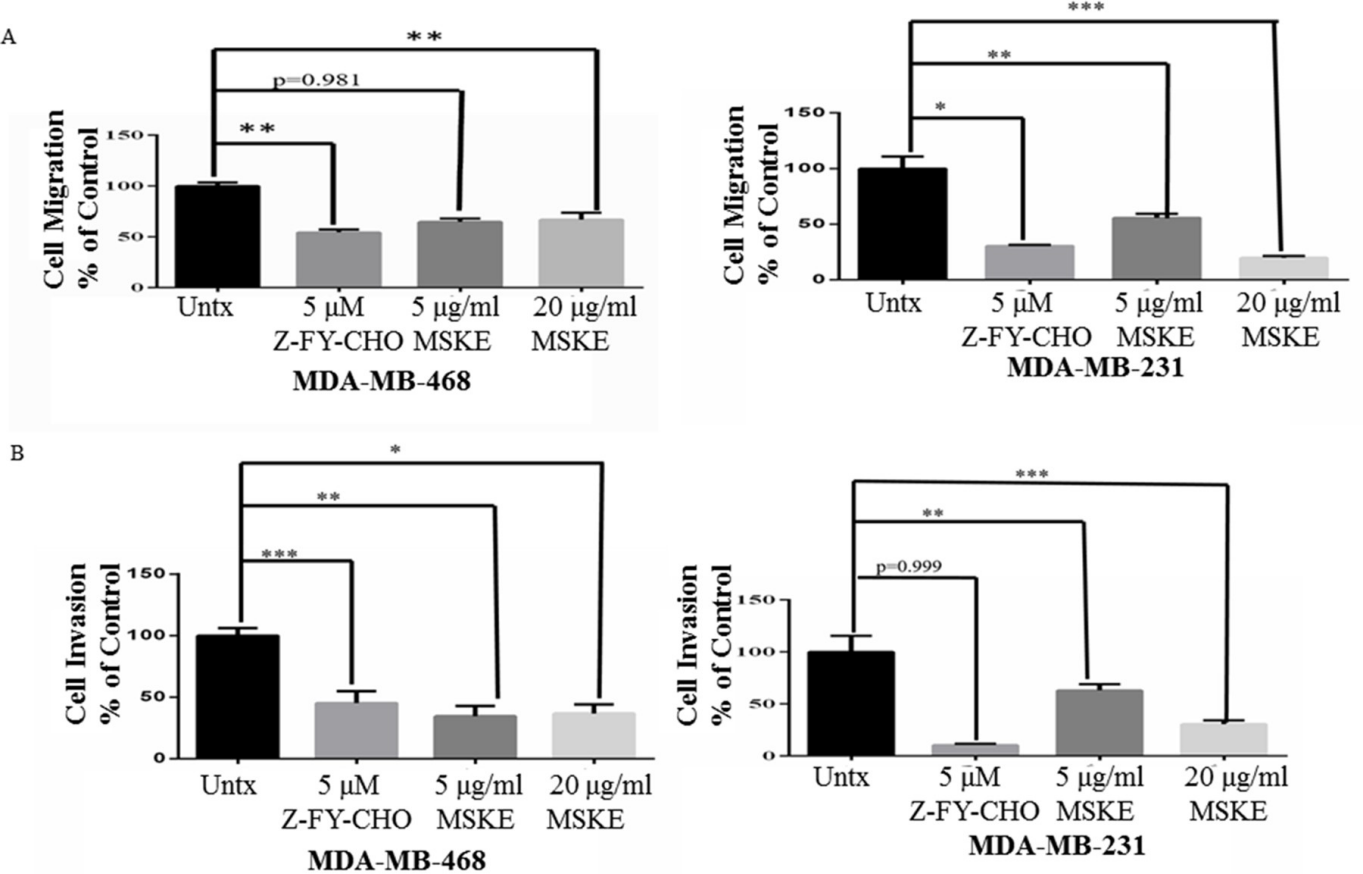
MSKE and Z-FY-CHO inhibit cell migration and invasion. (**A**) Migration on collagen and (**B**) Invasion on matrigel was performed using a boyden chamber with or without treatment of MDA-MB-468 or MDA-MB-231 cells with MSKE or Z-FY-CHO. Graphical data represents three independent experiments * means 0.05 > *p* value > 0.01, ** means 0.01 > *p* value > 0.001, and *** means *p* value < 0.001. Treatment with Z-FY-CHO and MSKE decreased migration and invasion in TNBC cells.

Next we examined if MSKE can inhibit cell migration and invasion comparable to Cat L inhibitor (Z-FY-CHO) as we had previously shown [12]. MDA-MB-468 and MDA-MB-231 cell migration and invasion decreased significantly upon treatment with MSKE or Z-FY-CHO (Fig 5). MSKE, a plant product has recently been shown to promote apoptosis of prostate cancer cells, but not normal cells [13], and revert EMT, in part by decreasing Snail expression [14]. Since MSKE has not been tested non-tumorigenic breast cells, we wanted to see if this same paradigm was true in breast cells. We probed for pro-apoptotic (Bax, cleaved caspase-7) and anti-apoptotic (Bcl-2) markers in MDA-MB-468 and MCF-10A (non-tumorigenic) cell lines, and following MSKE treatment, MDA-MB-468 cells had an increase in pro-apoptotic markers (Bax and cleaved caspase -7), but not MCF-10A cells, while Bcl-2 was only detected in MCF-10A cells and did not change with MSKE treatment (S3A Fig). Additionally, treatment with 20 μg/mL MSKE significantly inhibited cell viability in MDA-MB-468 cells, but not in MCF-10A cells (S3B Fig). Thus, MSKE only affects TNBC cancer cells and not non-tumorigenic cells and can antagonize cell migration and invasion in TNBC cells.

### MSKE and CUX1 siRNA, but not Z-FY-CHO inhibit TNBC cell viability and increase sensitivity to 4-hydroxytamoxifen

**Fig 6:**
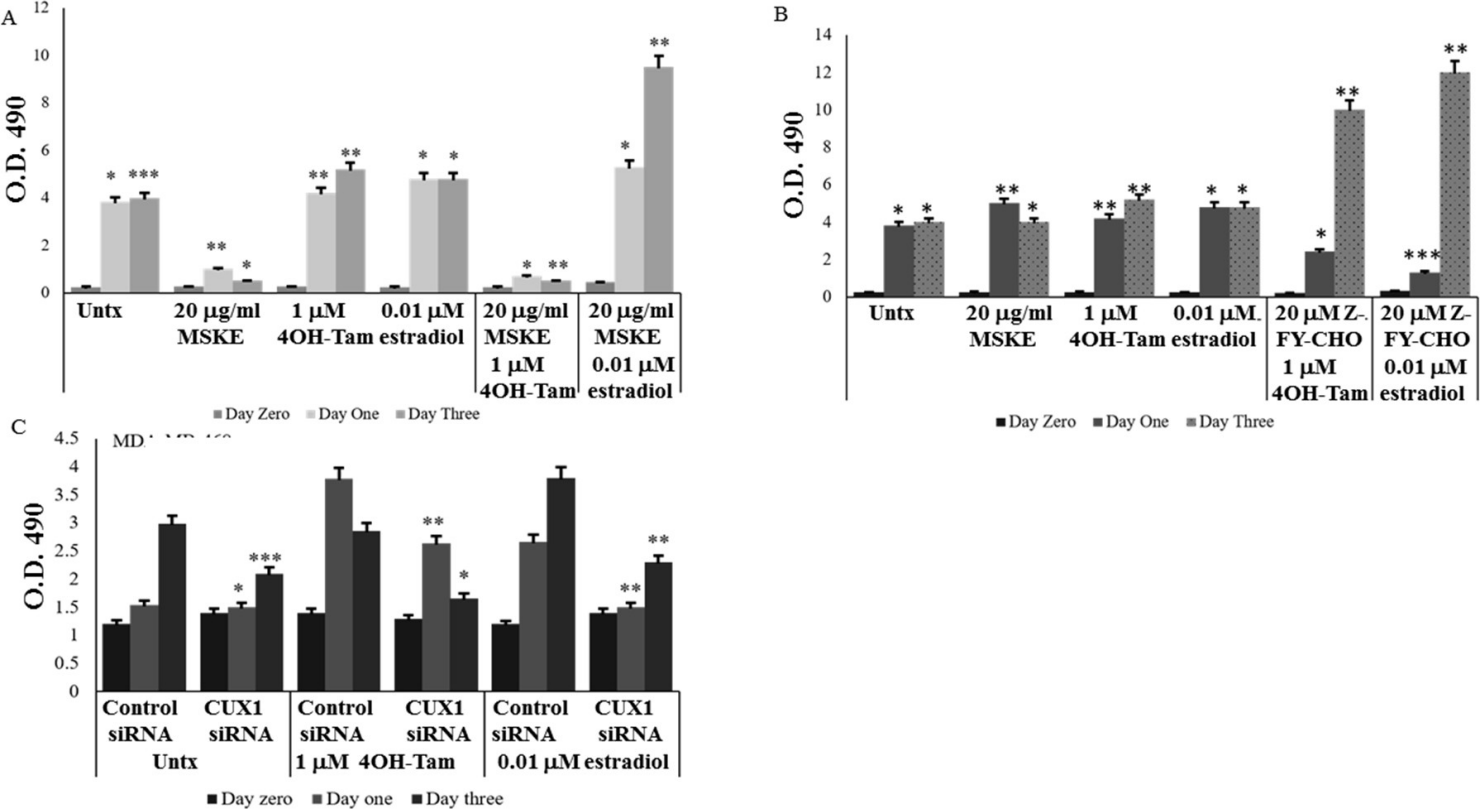
MSKE and CUX1 siRNA, but not Z-FY-CHO, sensitizes TNBC cells to 4-hydroxytamoxifen. (**A**) MDA-MB-468 cells were treated with MSKE (20 μg/ml), 4-hydroxytamoxifen (1μM) or 17-β estradiol (0.01 μM) or combinations of MSKE plus 4-hydroxytamoxifen or 17-β estradiol, followed by MTS assay for cell viability. (**B**) MDA-MB-468 cells were treated with Z-FY-CHO (20 μM), 4-hydroxytamoxifen (1μM) or 17-β estradiol (0.01 μM) or combinations of Z-FY-CHO plus 4-hydroxytamoxifen or 17-β estradiol, followed by MTS assay for cell viability. (**C**) MDA-MB-468 cells were transiently transfected with Control of CUX1 siRNA with or without 4-hydroxytamoxifen (1μM) or 17-β estradiol (0.01 μM) followed by MTS assay for cell viability. Graphical data represents three independent experiments * means 0.05 > *p* value > 0.01, ** means 0.01 > *p* value > 0.001, and *** means *p* value < 0.001.

In order to test whether MSKE may be a potential therapy for TNBC, and compare it to CUX1 knockdown or 4-hydroxytamoxifen, MDA-MB-468 cells were treated with MSKE (20 μg/mL), Z-FY-CHO (20 μM), control or CUX1 siRNA, or 4-hydroxytamoxifen (1 μM). The effect of 17-β estradiol (0.01 μM) was also tested to see whether the restoration of ER-α would render these cells responsive. 20 μg/mL MSKE significantly and drastically decreased the cell viability of MDA-MB-468 cells, while as expected 4-hydroxytamoxifen and 17-β estradiol had no effect (Fig 6A). Co-treatment of MSKE with 4-hydroxytamoxifen did not appear to show any further decrease, while co-treatment with 17-β estradiol led to increased proliferation (Fig 6A). Surprisingly, treatment with Z-FY-CHO alone did not decrease cell viability, in fact, co-treatment with 4-hydroxytamoxifen significantly increased viability by 72 h (Fig 6B). Compared to control siRNA which displayed increasing cell viability daily, CUX1 knockdown showed slower rate of cell proliferation which was more pronounced upon co-treatment with 4-hydroxytamoxifen, while co-treatment with 17-β estradiol had a similar slowed rate of proliferation as seen with CUX1 siRNA (Fig 6C). Overall, the data suggests that MSKE is the most effective agent in suppressing cell viability alone, or in combination with 4-hydroxytamoxifen in MDA-MB-468 cells as compared to CUX1 siRNA, with Z-FY-CHO being the least effective. It also shows that MSKE and Z-FY-CHO restore responsiveness of the TNBC cells to 17-β estradiol, suggesting that it may be binding to ER-α to increase cell viability.

## Discussion

Triple negative breast cancers (TNBCs) contains subgroups of aggressive forms of breast cancer that lack expression of estrogen receptor (ER), progesterone receptor (PR) and human epidermal growth factor receptor 2 (Her2/*neu*) [22]. Due to the lack of ER α, TNBCs are not susceptible to endocrine therapy, and with chemotherapy being the only option, there is high occurrence of relapse and toxicity [23]. Therefore, more novel therapies that are less toxic are wanting. The presence of Cat L in nucleus of TNBC and colon cancer have been associated with poor prognosis [9, 10]. Nuclear Cat L has been shown to cause the proteolytic cleave of CUX1 from the full length p200 to the p110 and p90 forms [24]. The p200 full-length protein has been found to act as a tumor suppressor; p110, but not p200, was capable of stimulating expression of Snail by direct binding to the Snail promoter, and also direct binding to E-cadherin promoter, leading to the repression of E cadherin and increased cell migration and invasion [11]. Other CUX1 isoforms have been reported, such as the p90, which seems to act cooperatively with the p110 isoform and displays similar DNA-binding and transcriptional activities [25].

Our previous research showed a positive feedback loop between Snail-Cat L-CUX1 in which Snail signaling via Cat L activation could lead to CUX1 proteolytic cleavage that led to CUX1 binding to Snail promoter to increase its transcription, while binding to E-cadherin to repress its transcription [12]. Our present study shows that TNBC cells and patient cancer tissue have higher Cat L expression/ activity and CUX1 expression compared to normal or ER-positive cells. For patient tissue used in western blots, unfortunately, receptor status was unavailable in pathology case reports to correlate it to Cat L activity. Although zymography is commonly used in conditioned media to detect activity by secreted protein, we show in this study that it can also detect cathepsin activity in whole cell lysate, cytoplasmic and nuclear extracts. In particular, we show by zymography that levels of active Cat L are higher within the nuclear compartment of TNBC, as compared to ER-positive cells. We also show that CUX1 cleavage products are higher in TNBC cell nucleus as compared to ER-positive cells, and that knocking down CUX1 with siRNA restored ER-α transcription and protein expression. In our current study we further show novel data that CUX1 can regulate the expression of ER-α by direct promoter binding, just as it has been shown between Snail transcription factor and ER-α [4]. Therefore, although CUX1 can increase Snail expression which subsequently binds ER-α promoter to suppress it [4, 11], we show that there is also direct binding of ER-α promoter by CUX1, which alludes to the multiple cooperative paths that cancer cells take to ensure silencing of tumor suppressors.

Muscadine grape skin extract (MSKE) with anthocyanin as the main bioactive component has shown its ability to inhibit prostate cancer cell growth and promote apoptosis *in vitro* without toxicity to normal prostate epithelial cells [13]. MSKE has been shown to revert EMT by decreasing the expression of Snail and causing re-expression of E-cadherin [14]. MSKE has completed Phase I and II clinical trials at John Hopkins University for treatment of localized prostate cancer [15], and is currently recruiting at Wake Forest University as a potential treatment for metastatic malignancies that have failed standard therapies; however, it has never been tested as a potential therapy for TNBC. This study was able to demonstrate that treatment of non-tumorigenic MCF10A cells with MSKE had no effect on cell viability and expression of Bcl-2 anti-apoptotic marker, or Cat L/CUX1 indicating that MSKE may not affect normal breast epithelial cells as compared to TNBC cells that exhibited an increase in apoptotic marker expression (Bax and cleaved caspase-7) and decreased cell viability upon MSKE treatment. The anti-estrogenic activity of 4-hydroxytamoxifen mediated by ER is well established and is the main reason for 4-hydroxytamoxifen treatment in ER-positive breast cancers, which does not work in ER-negative tumors that lack ER-α expression [26]. Our studies herein show that treatment with MSKE or Z-FY-CHO Cat L inhibitor cause the re-expression of ER-α in TNBC cells. ChIP analysis also confirms that treating with MSKE or Z-FY-CHO led to a decrease in binding of CUX1 to the ER-α promoter. From Fig 4E western blots, both MSKE and Z-FY-CHO decrease amounts of p110 and p90 CUX1 isoforms which coincides with decrease in promoter occupancy as shown in Fig 4A and 4B. Moreover, MDA-MB-468 cells treated with 5 μM Z-FY-CHO as well as MCF-7 Snail cells treated with 5 μg/ml MSKE still display high amounts of p200 CUX1 isoform yet ER-α promoter occupancy by CUX1 is low, suggesting that p110 and/or p90 but not p200 isoform can bind to ER- α promoter. Whether it is the p110 or the p90 isoform that binds to ER- α promoter is not clear, but previous studies suggest that the p110 and p90 isoforms work cooperatively and have similar DNA-binding and transcriptional activities [25]. Therefore, it is plausible that both the p110 and p90 isoforms directly bind to and repress ER-α promoter, although further studies are needed to definitively confirm this hypothesis. Both MSKE and Z-FY-CHO promote ER-α re-expression in TNBC cells. However, we noticed a difference in the localization of the re-expressed ER-α; MSKE resulted in nuclear ER-α re-expression, while Z-FY-CHO led to cytoplasmic ER-α re-expression. This may help explain the difference in the response to co-treatment of MDA-MB-468 cells with 4-hydroxytamoxifen and either MSKE or Z-FY-CHO; while MSKE drastically decreased cell viability in the presence or absence of 4-hydroxytamoxifen, Z-FY-CHO co-treated with 4-hydroxytamoxifen actually increased cell viability, similar to increased cell viability seen with co-treatment with 17-β estradiol. This was unexpected, as we had touted Z-FY-CHO as a great potential therapy in breast and cancer cells since it could decrease cell invasion and migration [12], results that were also confirmed in these studies. However, literature has shown that ER-α biology is complex with its response depending on its localization. Indeed, cytoplasmic and membrane ER-α has been associated with tamoxifen-resistant cells, and one study showed that long term treatment of MCF-7 cells with 4-hydroxytamoxifen led to re-localization of ER-α from the nucleus to the cytoplasm and enhanced its interaction with EGFR [27, 28]. This suggests that Z-FY-CHO is able to inhibit Snail and possibly migration/invasion similar to MSKE, but the CUX1 inhibition by Z-FY-CHO although sufficient to restore ER-α, has some other presently unknown mechanism that sequesters this ER-α in the cytoplasm and possibly makes it resistant to 4-hydroxytamoxifen. This underscores the importance of considering not just ER-α expression, but also its localization in combination therapies, going forward. If we had used western blot analysis alone, it would have been confusing as to why MSKE and Z-FY-CHO both restored ER-α expression, yet only MSKE was effective in combination treatments with 4-hydroxytamoxifen. Further confirmatory studies are required to conclude that Z-FY-CHO may be effective alone in reducing cell migration and invasion, but not in combination with 4-hydroxytamoxifen. Moreover, in future, we also need to test different doses of 4-hydroxytamoxifen, since in this study we used the lowest dose (1 μM) similar to metabolite concentrations measured when low-dose tamoxifen regimens are utilized [29–31].

In conclusion, while these molecular pathways could be relevant to TNBC, more studies are needed to comprehensively determine the precise and the relative contributions of each and a combination of these pathways to the pathogenesis of TNBC. Such studies will be valuable not only for gaining better understanding on the pathogenesis of TNBC but also allow identification and development of novel biomarkers/targets for diagnostic and therapeutic approaches for prevention and treatment of these types of breast cancer. There is a major need to better understand the molecular basis of TNBC and to develop revolutionizing treatment regimens by replacing interventions that have life-threatening toxicities (chemotherapy) with ones that are safe and effective such as using natural products. Our current study puts forward a provoking thought that using MSKE alone or in conjunction with conventional therapies, may not only treat TNBC, but may be protective to normal cells that are usually damaged during treatment. Thus, MSKE may represent a novel and viable treatment strategy for TNBC that warrants further study.

## Supporting information

S1 Fig TNBC cells have higher levels of Cat L and CUX1 as compared to ER-positive breast cancer cells. Immunofluorescence was performed on the TNBC and ER-positive cells using Cat L and CUX1 antibodies.
(TIF)

S2 Fig Overexpression of Snail leads to TNBC signature. Western blot analysis using ER-α, PR, HER-2 antibodies were performed on MCF-7 parental, MDA-MB-468, MCF-7 neo and MCF-7 Snail cells.
(TIF)

S3 Fig MSKE inhibits cell viability in MDA-MB-468 TNBC cells but does not affect MCF-10A non-tumorigenic cells. (**A**) MCF10A non-tumorigenic or MDA-MB-468 TNBC cells were treated with ethanol control (Untx) or 20 μg/mL MSKE for 24 h, followed by western blot analysis with pro-apoptotic markers (Bax, Cleaved Caspase-7) or anti-apoptotic marker (Bcl-2). (**B**) Cell viability following MSKE treatment was analyzed using MTS assay. (**C**) Western blot analysis for Snail, ER-a, Cat L and CUX1 was performed on MCF10A cells treated with MSKE or Z-FY-CHO for 3 days. Actin was utilized as a loading control. Graphical data represents three independent experiments * means 0.05 > *p* value > 0.01, ** means 0.01 > *p* value > 0.001, and *** means *p* value < 0.001.
(TIF)

S1 Table Patient information for lysates used for Zymography in Fig 1. (TIF)

S2 Table Patient information and statistical analysis of Cat L and CUX1 mRNA Seq data from cbioportal. The mRNA expression (RNA Seq RPKM, Reads Per Kilobase Million) values for CTSL (Cat L) and CUX1 from the samples included in the 3-Gene classifier subtype were downloaded from the cbioportal plot page and saved as CTSL.txt and CUX1.txt for statistical analyses. Statistical analysis was performed by One-way ANOVA with post-hoc Tukey HSD (honestly significant difference) using the R package multcomp.
(PDF)
