# Supplementary figures and images for: CCAAT-displacement protein/cut homeobox transcription factor (CUX1) represses estrogen receptor-alpha (ER-α) in triple-negative breast cancer cells and can be antagonized by muscadine grape skin extract (MSKE)
Source: PLoS One. 2019 Apr 9;14(4):e0214844. doi: 10.1371/journal.pone.0214844 (PMC6460785; doi:10.1371/journal.pone.0214844)

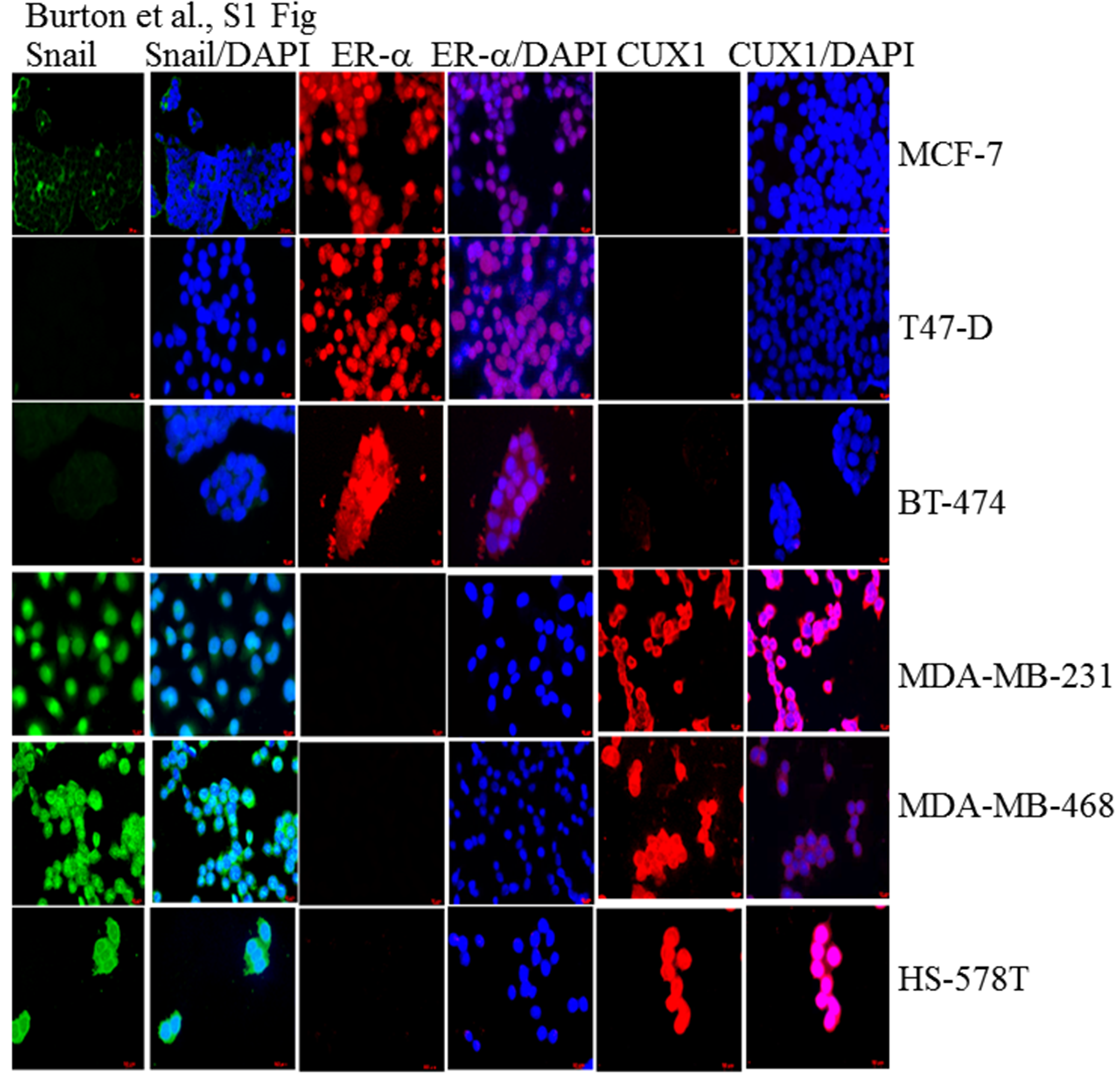

Supplement: S1 Fig — Immunofluorescence was performed on the TNBC and ER-positive cells using Cat L and CUX1 antibodies. (TIF) [file pone.0214844.s001.tif]

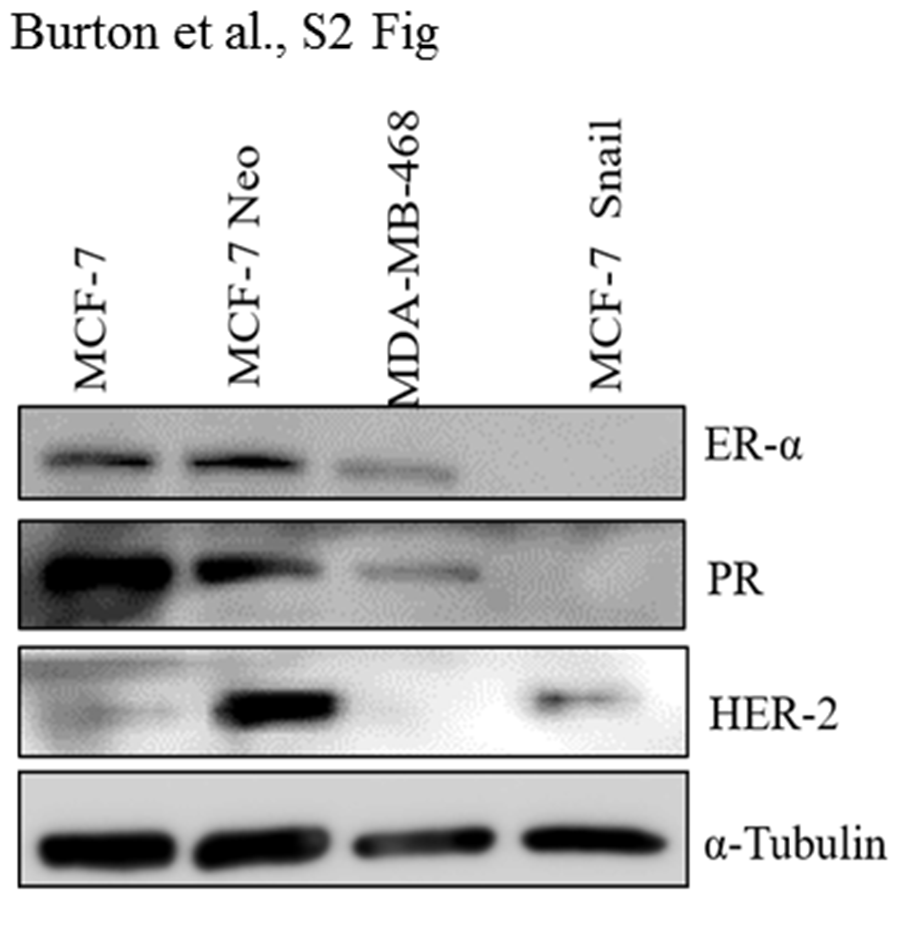

Supplement: S2 Fig — Western blot analysis using ER-α, PR, HER-2 antibodies were performed on MCF-7 parental, MDA-MB-468, MCF-7 neo and MCF-7 Snail cells. (TIF) [file pone.0214844.s002.tif]

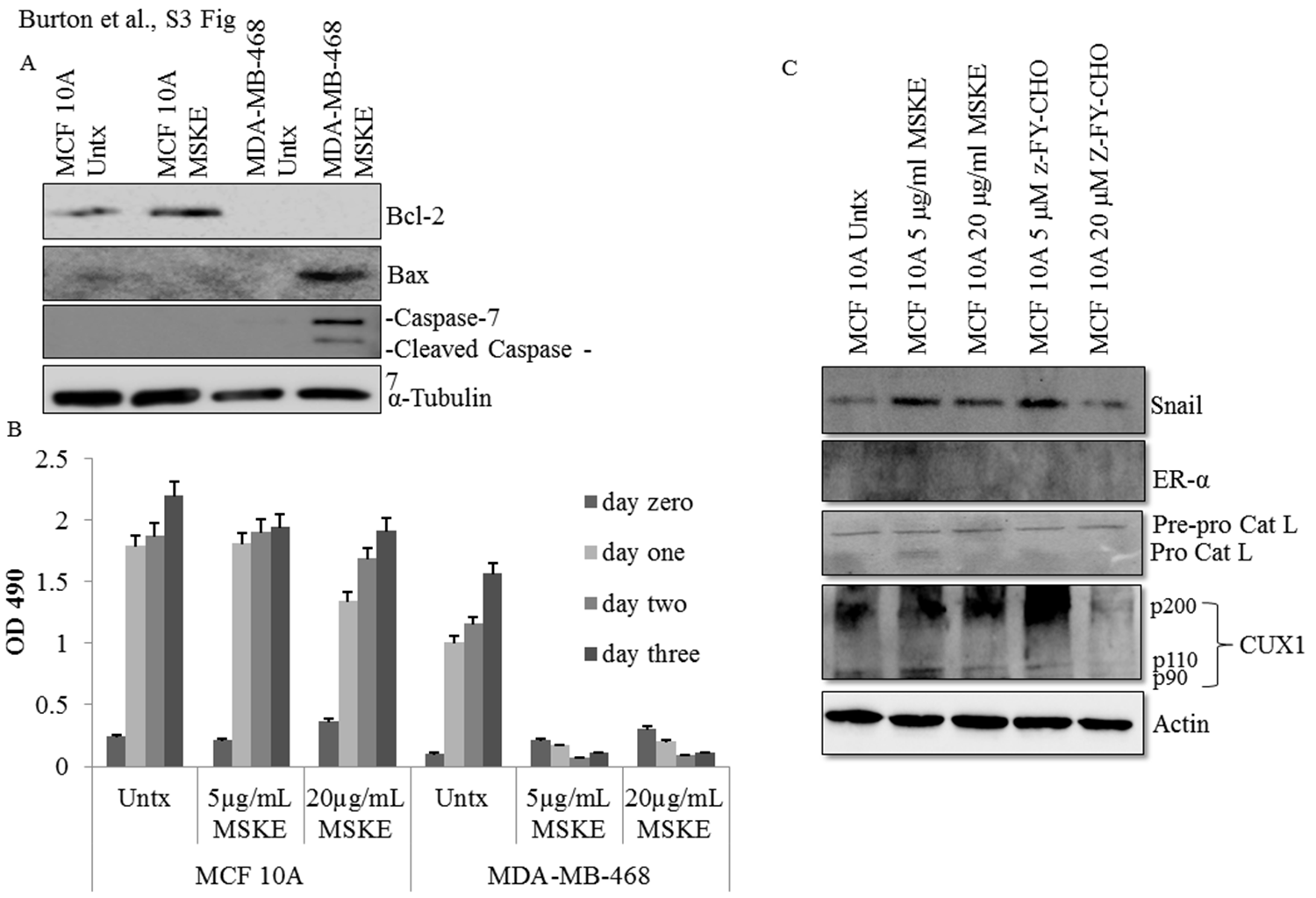

Supplement: S3 Fig — (A) MCF10A non-tumorigenic or MDA-MB-468 TNBC cells were treated with ethanol control (Untx) or 20 μg/mL MSKE for 24 h, followed by western blot analysis with pro-apoptotic markers (Bax, Cleaved Caspase-7) or anti-apoptotic marker (Bcl-2). (B) Cell viability following MSKE treatment was analyzed using MTS assay. (C) Western blot analysis for Snail, ER-a, Cat L and CUX1 was performed on MCF10A cells treated with MSKE or Z-FY-CHO for 3 days. Actin was utilized as a loading control. Graphical data represents three independent experiments * means 0.05 > p value > 0.01, ** means 0.01 > p value > 0.001, and *** means p value < 0.001. (TIF) [file pone.0214844.s003.tif]

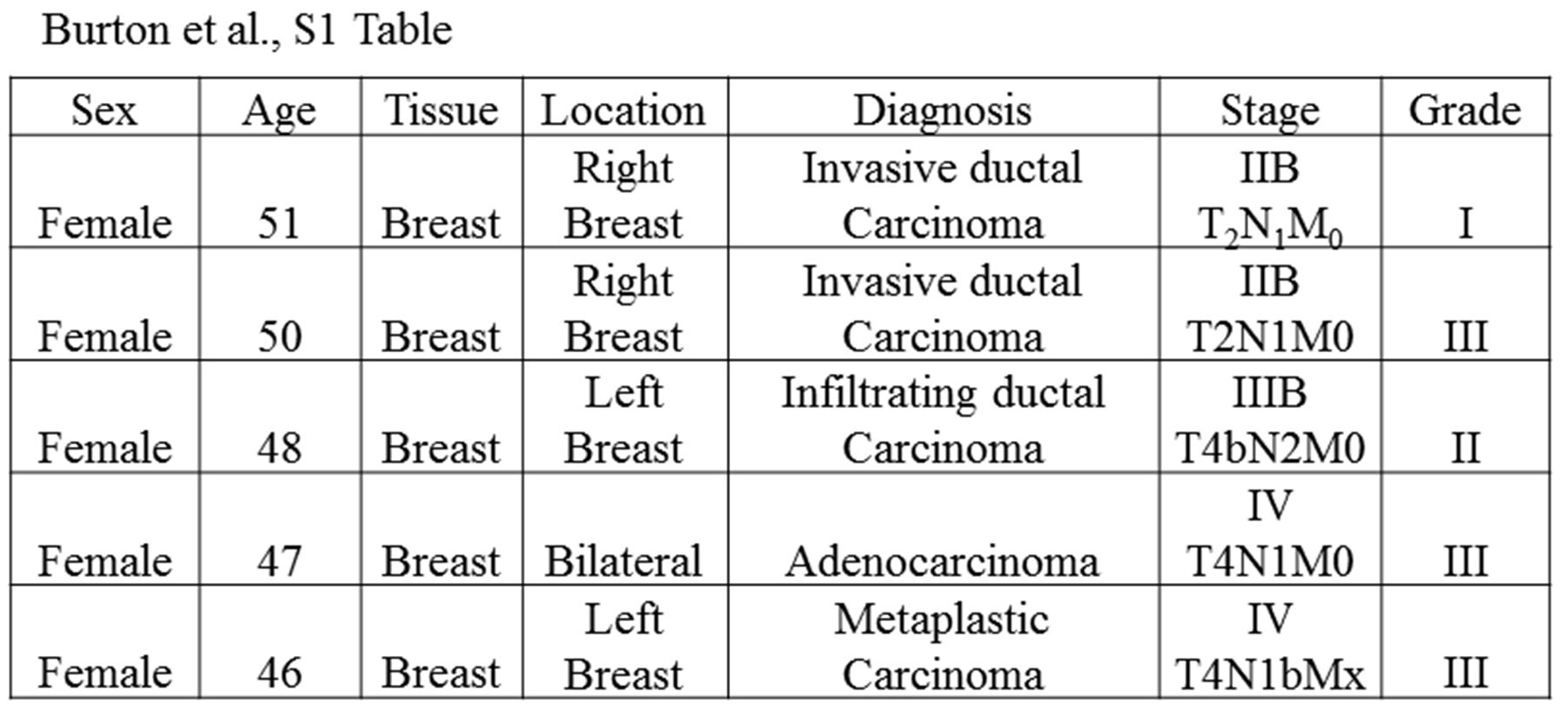

Supplement: S1 Table — (TIF) [file pone.0214844.s004.tif]
