# Supplementary material for: CCAAT-displacement protein/cut homeobox transcription factor (CUX1) represses estrogen receptor-alpha (ER-α) in triple-negative breast cancer cells and can be antagonized by muscadine grape skin extract (MSKE)
Source: PLoS One. 2019 Apr 9;14(4):e0214844. doi: 10.1371/journal.pone.0214844 (PMC6460785; doi:10.1371/journal.pone.0214844)
Supplement: S2 Table — The mRNA expression (RNA Seq RPKM, Reads Per Kilobase Million) values for CTSL (Cat L) and CUX1 from the samples included in the 3-Gene classifier subtype were downloaded from the cbioportal plot page and saved as CTSL.txt and CUX1.txt for statistical analyses. Statistical analysis was performed by One-way ANOVA with post-hoc Tukey HSD (honestly significant difference) using the R package multcomp. (PDF) [file pone.0214844.s005.pdf]

Sample Id	3-Gene classi	CTSL: mRNA expression (RN	ER-/HER2-	ER+/HER- Hig	ER+/HER2- Lc
MB-0000	ER-/HER2-	10.074874	10.074874	9.547659	10.570197
MB-0100	ER-/HER2-	9.203283	9.203283	10.999749	9.662606
MB-0115	ER-/HER2-	11.182727	11.182727	10.552298	10.669429
MB-0149	ER-/HER2-	9.686657	9.686657	9.506558	9.566815
MB-0150	ER-/HER2-	10.302505	10.302505	7.929349	9.559906
MB-0157	ER-/HER2-	11.321323	11.321323	11.526585	9.837235
MB-0164	ER-/HER2-	11.89128	11.89128	11.022788	10.163683
MB-0174	ER-/HER2-	12.320848	12.320848	10.202884	9.89235
MB-0179	ER-/HER2-	10.293014	10.293014	10.141794	10.049783
MB-0206	ER-/HER2-	12.3358	12.3358	8.865289	10.180187
MB-0209	ER-/HER2-	12.92822	12.92822	9.443071	9.813701
MB-0214	ER-/HER2-	9.902631	9.902631	10.122889	9.837119
MB-0238	ER-/HER2-	11.590513	11.590513	10.753348	10.455735
MB-0249	ER-/HER2-	11.692421	11.692421	10.110437	10.560879
MB-0259	ER-/HER2-	9.727652	9.727652	10.565411	10.006762
MB-0265	ER-/HER2-	10.428076	10.428076	11.270627	11.250565
MB-0269	ER-/HER2-	13.010996	13.010996	9.434573	9.785267
MB-0278	ER-/HER2-	8.969407	8.969407	9.234262	9.329582
MB-0284	ER-/HER2-	10.951151	10.951151	9.738255	10.285775
MB-0285	ER-/HER2-	11.240185	11.240185	9.696775	9.742329
MB-0286	ER-/HER2-	10.843784	10.843784	9.422678	9.316393
MB-0289	ER-/HER2-	12.752417	12.752417	10.336715	10.617633
MB-0292	ER-/HER2-	10.719445	10.719445	11.11603	10.770026
MB-0293	ER-/HER2-	10.539198	10.539198	8.924406	9.590881
MB-0303	ER-/HER2-	11.593061	11.593061	9.967956	10.092974
MB-0316	ER-/HER2-	10.800178	10.800178	9.874404	10.920228
MB-0318	ER-/HER2-	8.586508	8.586508	9.975526	10.551519
MB-0340	ER-/HER2-	11.721069	11.721069	11.132973	10.093199
MB-0352	ER-/HER2-	10.996992	10.996992	9.891977	10.112902
MB-0354	ER-/HER2-	11.490807	11.490807	10.618041	10.554601
MB-0365	ER-/HER2-	11.495996	11.495996	10.042043	10.495371
MB-0372	ER-/HER2-	9.466893	9.466893	9.797694	10.749492
MB-0375	ER-/HER2-	10.193403	10.193403	10.563777	9.732535
MB-0396	ER-/HER2-	10.377976	10.377976	10.347866	9.43
MB-0399	ER-/HER2-	11.133667	11.133667	10.753313	10.508641
MB-0400	ER-/HER2-	10.920964	10.920964	9.566125	10.398645
MB-0401	ER-/HER2-	11.752771	11.752771	10.399223	10.345083
MB-0414	ER-/HER2-	11.036171	11.036171	10.277674	10.313197
MB-0420	ER-/HER2-	10.96248	10.96248	10.171126	10.227911
MB-0424	ER-/HER2-	11.504265	11.504265	9.822406	9.326726
MB-0436	ER-/HER2-	11.069391	11.069391	9.684751	10.417907
MB-0446	ER-/HER2-	12.525577	12.525577	10.289918	11.682904

MB-0464	ER-/HER2-	10.295432		10.295432	9.492757	10.406776
MB-0476	ER-/HER2-	11.195504		11.195504	10.188584	10.608847
MB-0481	ER-/HER2-	10.605285		10.605285	11.16494	10.488611
MB-0488	ER-/HER2-	10.691786		10.691786	9.957136	9.78858
MB-0489	ER-/HER2-	10.173819		10.173819	10.345693	10.219419
MB-0494	ER-/HER2-	12.007152		12.007152	10.08963	9.907967
MB-0495	ER-/HER2-	10.972775		10.972775	10.57486	10.11634
MB-0500	ER-/HER2-	10.750715		10.750715	10.699423	9.427589
MB-0502	ER-/HER2-	13.058459		13.058459	10.66984	9.825336
MB-0516	ER-/HER2-	11.182362		11.182362	11.323513	10.608432
MB-0525	ER-/HER2-	10.916376		10.916376	10.070066	10.865556
MB-0540	ER-/HER2-	10.367863		10.367863	10.612029	11.254058
MB-0558	ER-/HER2-	10.772019		10.772019	10.356447	10.760923
MB-0581	ER-/HER2-	11.141802		11.141802	9.762292	10.437545
MB-0582	ER-/HER2-	10.657896		10.657896	10.543753	9.938955
MB-0608	ER-/HER2-	11.570062		11.570062	10.112312	10.561095
MB-0613	ER-/HER2-	11.928577		11.928577	9.32004	8.699085
MB-0617	ER-/HER2-	10.506879		10.506879	9.695369	9.186847
MB-0620	ER-/HER2-	10.385477		10.385477	11.422731	9.897777
MB-0627	ER-/HER2-	11.480479		11.480479	10.933062	10.206151
MB-0643	ER-/HER2-	11.111476		11.111476	10.310597	10.994763
MB-0658	ER-/HER2-	10.799249		10.799249	11.369163	9.105898
MB-0659	ER-/HER2-	8.732809		8.732809	9.855314	10.772019
MB-0664	ER-/HER2-	11.142689		11.142689	10.297466	11.050314
MB-0869	ER-/HER2-	10.808615		10.808615	10.489395	9.386074
MB-0874	ER-/HER2-	11.098426		11.098426	10.631111	10.533266
MB-0893	ER-/HER2-	13.45017		13.45017	10.290009	10.681164
MB-0906	ER-/HER2-	11.189773		11.189773	10.987813	10.143807
MB-2556	ER-/HER2-	10.639784		10.639784	10.47478	10.750216
MB-2753	ER-/HER2-	11.271497		11.271497	10.670114	10.334814
MB-2764	ER-/HER2-	11.133667		11.133667	11.077654	10.193443
MB-2827	ER-/HER2-	11.021368		11.021368	10.585144	10.444788
MB-2834	ER-/HER2-	10.747374		10.747374	10.228296	9.751725
MB-2842	ER-/HER2-	12.639091		12.639091	9.928551	9.249606
MB-2846	ER-/HER2-	10.624761		10.624761	10.251026	10.46528
MB-2849	ER-/HER2-	10.44326		10.44326	10.728044	10.510495
MB-2857	ER-/HER2-	11.340433		11.340433	10.70983	9.986185
MB-2912	ER-/HER2-	10.585505		10.585505	9.696846	10.393312
MB-2917	ER-/HER2-	11.509663		11.509663	9.938067	10.630074
MB-2929	ER-/HER2-	11.144046		11.144046	10.585666	10.045829
MB-2957	ER-/HER2-	11.552791		11.552791	10.029035	9.96933
MB-2963	ER-/HER2-	11.85599		11.85599	10.950702	10.050351
MB-2993	ER-/HER2-	10.50988		10.50988	10.490129	9.383528

MB-3006	ER-/HER2-	11.746653		11.746653	10.30841	9.136009
MB-3014	ER-/HER2-	10.330227		10.330227	10.71285	10.28566
MB-3046	ER-/HER2-	10.144549		10.144549	9.885376	10.090195
MB-3057	ER-/HER2-	11.02749		11.02749	9.881116	10.488611
MB-3058	ER-/HER2-	10.827904		10.827904	9.576476	10.919344
MB-3062	ER-/HER2-	11.488443		11.488443	8.231978	10.608193
MB-3063	ER-/HER2-	11.61187		11.61187	11.090749	10.202325
MB-3067	ER-/HER2-	11.078602		11.078602	11.691638	10.713464
MB-3123	ER-/HER2-	11.277768		11.277768	9.835181	10.560879
MB-3153	ER-/HER2-	10.436454		10.436454	10.223879	10.705165
MB-3165	ER-/HER2-	10.480448		10.480448	10.515899	10.506879
MB-3211	ER-/HER2-	11.026693		11.026693	10.718162	9.910066
MB-3271	ER-/HER2-	10.924255		10.924255	8.590884	10.551204
MB-3277	ER-/HER2-	11.183964		11.183964	11.262337	10.014486
MB-3292	ER-/HER2-	10.865805		10.865805	10.959649	10.320227
MB-3297	ER-/HER2-	10.339947		10.339947	10.221605	10.867442
MB-3363	ER-/HER2-	10.688199		10.688199	10.084621	9.576055
MB-3367	ER-/HER2-	11.512643		11.512643	11.005528	10.399087
MB-3395	ER-/HER2-	11.154144		11.154144	10.312562	10.458573
MB-3396	ER-/HER2-	11.149488		11.149488	9.830043	10.294823
MB-3453	ER-/HER2-	11.035815		11.035815	10.909235	10.48741
MB-3476	ER-/HER2-	10.210237		10.210237	11.482617	9.731953
MB-3479	ER-/HER2-	11.481876		11.481876	10.880764	9.987574
MB-3500	ER-/HER2-	10.794822		10.794822	10.386421	8.916544
MB-3502	ER-/HER2-	11.324162		11.324162	10.952986	10.235016
MB-3567	ER-/HER2-	10.722647		10.722647	9.948472	10.778496
MB-3582	ER-/HER2-	11.610352		11.610352	10.884623	10.084892
MB-3702	ER-/HER2-	9.88738		9.88738	10.193621	10.503827
MB-3706	ER-/HER2-	10.79662		10.79662	10.776987	9.834416
MB-3752	ER-/HER2-	10.801275		10.801275	10.084621	9.550583
MB-4146	ER-/HER2-	9.463186		9.463186	10.340787	10.57615
MB-4408	ER-/HER2-	10.171126		10.171126	9.870296	9.834634
MB-4621	ER-/HER2-	10.036232		10.036232	10.170401	10.148086
MB-4622	ER-/HER2-	10.7453		10.7453	9.887792	10.006823
MB-4667	ER-/HER2-	10.322458		10.322458	10.55669	9.566934
MB-4679	ER-/HER2-	9.558644		9.558644	9.953782	9.995978
MB-4694	ER-/HER2-	10.387493		10.387493	10.475517	10.323682
MB-4696	ER-/HER2-	10.841415		10.841415	10.57615	10.039767
MB-4707	ER-/HER2-	10.964114		10.964114	10.357116	10.685609
MB-4711	ER-/HER2-	10.360132		10.360132	10.618762	9.989254
MB-4714	ER-/HER2-	9.807303		9.807303	9.998216	9.252758
MB-4715	ER-/HER2-	10.240926		10.240926	10.630074	9.535026
MB-4717	ER-/HER2-	10.571619		10.571619	10.984364	10.001456

MB-4732	ER-/HER2-	10.165481		10.165481	9.491863	10.059602
MB-4733	ER-/HER2-	10.794797		10.794797	12.328106	10.246159
MB-4757	ER-/HER2-	10.254126		10.254126	9.873587	10.109503
MB-4758	ER-/HER2-	10.310631		10.310631	10.162255	10.749492
MB-4769	ER-/HER2-	10.657896		10.657896	10.46665	9.089322
MB-4770	ER-/HER2-	10.725582		10.725582	12.113253	10.853439
MB-4782	ER-/HER2-	9.084423		9.084423	10.553531	10.156708
MB-4792	ER-/HER2-	10.252971		10.252971	10.677173	9.958468
MB-4809	ER-/HER2-	10.573676		10.573676	10.332572	10.86155
MB-4828	ER-/HER2-	9.451615		9.451615	10.588242	10.068721
MB-4859	ER-/HER2-	9.985646		9.985646	10.855039	11.253395
MB-4865	ER-/HER2-	10.210496		10.210496	10.518093	11.201717
MB-4876	ER-/HER2-	11.013272		11.013272	11.332967	10.139679
MB-4880	ER-/HER2-	10.327199		10.327199	10.164934	10.280644
MB-4881	ER-/HER2-	10.383044		10.383044	11.185635	9.386512
MB-4888	ER-/HER2-	10.328172		10.328172	10.866678	10.757172
MB-4893	ER-/HER2-	10.608432		10.608432	10.087042	10.637569
MB-4911	ER-/HER2-	10.562253		10.562253	11.381911	10.472116
MB-4938	ER-/HER2-	10.635637		10.635637	10.31267	10.465635
MB-4942	ER-/HER2-	11.197149		11.197149	10.150266	10.193621
MB-4945	ER-/HER2-	9.322887		9.322887	10.492213	10.064987
MB-4974	ER-/HER2-	11.473101		11.473101	10.774559	10.286129
MB-4982	ER-/HER2-	10.095273		10.095273	10.73327	11.238632
MB-4993	ER-/HER2-	10.75482		10.75482	10.208735	10.48045
MB-5008	ER-/HER2-	10.250741		10.250741	10.856988	10.519396
MB-5041	ER-/HER2-	10.589844		10.589844	11.214849	9.892084
MB-5052	ER-/HER2-	10.766185		10.766185	10.139469	10.025902
MB-5057	ER-/HER2-	10.536184		10.536184	11.116939	10.76539
MB-5058	ER-/HER2-	10.698928		10.698928	11.335501	9.802586
MB-5065	ER-/HER2-	11.159855		11.159855	10.502135	10.523995
MB-5070	ER-/HER2-	10.905134		10.905134	9.219458	9.047628
MB-5072	ER-/HER2-	10.48741		10.48741	10.220687	10.880764
MB-5100	ER-/HER2-	10.176072		10.176072	10.983071	10.560227
MB-5115	ER-/HER2-	9.895477		9.895477	9.80593	8.942652
MB-5126	ER-/HER2-	10.363394		10.363394	10.913534	11.664224
MB-5138	ER-/HER2-	10.970198		10.970198	10.836907	10.659624
MB-5145	ER-/HER2-	10.028543		10.028543	10.533266	9.650979
MB-5148	ER-/HER2-	10.190647		10.190647	10.476738	9.882834
MB-5154	ER-/HER2-	10.020886		10.020886	10.410637	10.669429
MB-5157	ER-/HER2-	10.249839		10.249839	10.217419	10.200736
MB-5162	ER-/HER2-	12.662213		12.662213	11.831332	9.976605
MB-5173	ER-/HER2-	10.813882		10.813882	9.820064	10.033348
MB-5188	ER-/HER2-	11.250565		11.250565	10.82905	10.21116

MB-5205	ER-/HER2-	10.659295	10.659295	9.886553	10.122201
MB-5208	ER-/HER2-	10.398778	10.398778	10.71427	10.030499
MB-5209	ER-/HER2-	10.146311	10.146311	10.757216	10.597752
MB-5222	ER-/HER2-	10.687479	10.687479	10.073692	10.533962
MB-5223	ER-/HER2-	10.325296	10.325296	10.272735	10.603106
MB-5225	ER-/HER2-	11.202807	11.202807	10.371788	11.117205
MB-5232	ER-/HER2-	10.642703	10.642703	10.960089	10.811984
MB-5235	ER-/HER2-	9.735076	9.735076	11.192296	10.554601
MB-5236	ER-/HER2-	10.49377	10.49377	9.836235	10.149005
MB-5258	ER-/HER2-	9.9867	9.9867	9.569249	9.310152
MB-5294	ER-/HER2-	9.831569	9.831569	10.269779	9.68821
MB-5295	ER-/HER2-	10.293014	10.293014	11.178631	10.385423
MB-5298	ER-/HER2-	10.327199	10.327199	9.814134	9.624664
MB-5299	ER-/HER2-	9.801963	9.801963	10.545742	9.968118
MB-5311	ER-/HER2-	11.187593	11.187593	10.644094	9.663305
MB-5323	ER-/HER2-	10.462465	10.462465	9.883739	10.224875
MB-5335	ER-/HER2-	9.831362	9.831362	10.870941	11.245259
MB-5348	ER-/HER2-	10.653803	10.653803	10.053443	9.416736
MB-5350	ER-/HER2-	10.774016	10.774016	9.534605	9.864731
MB-5378	ER-/HER2-	10.398645	10.398645	10.295023	10.928108
MB-5390	ER-/HER2-	11.459299	11.459299	10.463738	9.492617
MB-5392	ER-/HER2-	11.126603	11.126603	11.022124	10.152228
MB-5408	ER-/HER2-	11.70853	11.70853	9.20946	10.317343
MB-5421	ER-/HER2-	9.853595	9.853595	10.237812	10.031701
MB-5427	ER-/HER2-	8.810958	8.810958	9.467922	9.970353
MB-5431	ER-/HER2-	10.293014	10.293014	9.786938	11.053074
MB-5440	ER-/HER2-	10.955333	10.955333	10.061312	10.51989
MB-5442	ER-/HER2-	10.684322	10.684322	9.194672	10.639973
MB-5446	ER-/HER2-	10.553992	10.553992	9.673011	10.909512
MB-5450	ER-/HER2-	10.894777	10.894777	11.159855	9.21355
MB-5453	ER-/HER2-	9.650979	9.650979	10.955333	10.342623
MB-5465	ER-/HER2-	10.533266	10.533266	10.813882	10.231122
MB-5468	ER-/HER2-	11.377655	11.377655	11.135016	11.323974
MB-5482	ER-/HER2-	10.675885	10.675885	10.829266	9.894345
MB-5511	ER-/HER2-	10.637407	10.637407	9.844617	9.895777
MB-5526	ER-/HER2-	10.32686	10.32686	12.00193	10.123136
MB-5527	ER-/HER2-	10.022761	10.022761	9.74177	11.055087
MB-5529	ER-/HER2-	10.850932	10.850932	8.85381	9.741261
MB-5531	ER-/HER2-	10.39046	10.39046	9.784068	10.239395
MB-5534	ER-/HER2-	10.499372	10.499372	9.312336	10.179469
MB-5547	ER-/HER2-	9.343514	9.343514	10.103668	9.938109
MB-5548	ER-/HER2-	10.753899	10.753899	10.269515	10.582653
MB-5549	ER-/HER2-	10.362379	10.362379	9.96759	10.643377

MB-5551	ER-/HER2-	10.406857	10.406857	8.789726	10.545637
MB-5559	ER-/HER2-	9.22096	9.22096	9.498478	10.389998
MB-5560	ER-/HER2-	11.133528	11.133528	7.748055	9.288294
MB-5565	ER-/HER2-	11.328677	11.328677	10.680713	10.366495
MB-5566	ER-/HER2-	9.695336	9.695336	10.841415	10.398645
MB-5572	ER-/HER2-	9.987552	9.987552	10.206586	10.299855
MB-5588	ER-/HER2-	9.097008	9.097008	10.225224	11.11603
MB-5602	ER-/HER2-	10.94904	10.94904	10.795348	10.508641
MB-5616	ER-/HER2-	10.927903	10.927903	10.533097	9.749271
MB-5633	ER-/HER2-	10.677962	10.677962	11.285105	9.97868
MB-5634	ER-/HER2-	9.870307	9.870307	10.195007	9.801963
MB-5651	ER-/HER2-	10.225561	10.225561	10.806226	9.947863
MB-5652	ER-/HER2-	9.602834	9.602834	10.263226	10.261234
MB-5655	ER-/HER2-	9.353814	9.353814	11.122977	9.472007
MB-6036	ER-/HER2-	11.731744	11.731744	10.845303	10.528281
MB-6052	ER-/HER2-	10.627945	10.627945	11.05089	10.30253
MB-6055	ER-/HER2-	11.602012	11.602012	10.455735	10.598514
MB-6058	ER-/HER2-	11.208063	11.208063	11.180725	9.682177
MB-6062	ER-/HER2-	11.288153	11.288153	9.495184	9.897905
MB-6085	ER-/HER2-	11.13867	11.13867	11.244862	10.805337
MB-6122	ER-/HER2-	11.576211	11.576211	9.284261	10.118699
MB-6143	ER-/HER2-	10.199443	10.199443	10.012508	10.182271
MB-6144	ER-/HER2-	10.920228	10.920228	10.548715	10.423638
MB-6152	ER-/HER2-	10.768385	10.768385	10.195222	9.234834
MB-6169	ER-/HER2-	10.461474	10.461474	9.302515	10.215849
MB-6178	ER-/HER2-	10.627945	10.627945	10.163356	10.476674
MB-6187	ER-/HER2-	9.563948	9.563948	10.075656	10.168946
MB-6188	ER-/HER2-	11.103373	11.103373	9.283509	9.351939
MB-6223	ER-/HER2-	10.57615	10.57615	9.545085	10.021795
MB-6224	ER-/HER2-	11.584475	11.584475	8.687667	10.059205
MB-6228	ER-/HER2-	10.698818	10.698818	8.494219	10.643377
MB-6237	ER-/HER2-	11.323974	11.323974	9.727588	10.382571
MB-6242	ER-/HER2-	11.664224	11.664224	10.589242	9.754423
MB-6245	ER-/HER2-	9.066309	9.066309	9.673849	9.938067
MB-6248	ER-/HER2-	11.264521	11.264521	10.458988	9.659054
MB-6305	ER-/HER2-	11.144046	11.144046	8.766415	11.177765
MB-6318	ER-/HER2-	10.290665	10.290665	9.593148	9.656502
MB-6336	ER-/HER2-	10.387976	10.387976	10.049322	9.408491
MB-7007	ER-/HER2-	11.294018	11.294018	9.793267	11.003741
MB-7008	ER-/HER2-	11.04082	11.04082	9.837925	9.718927
MB-7009	ER-/HER2-	10.160884	10.160884	9.102069	10.817394
MB-7012	ER-/HER2-	11.017725	11.017725	6.675191	10.038889
MB-7017	ER-/HER2-	10.684588	10.684588	9.766705	10.52463

MB-7023	ER-/HER2-	11.62832	11.62832	9.116332	9.880672
MB-7025	ER-/HER2-	11.526545	11.526545	10.423616	10.552947
MB-7030	ER-/HER2-	9.899449	9.899449	10.468201	9.819122
MB-7036	ER-/HER2-	11.422518	11.422518	9.818406	10.056903
MB-7038	ER-/HER2-	9.445425	9.445425	9.486446	10.312842
MB-7039	ER-/HER2-	10.592447	10.592447	9.978838	11.1206
MB-7045	ER-/HER2-	10.716583	10.716583	9.841281	10.215303
MB-7049	ER-/HER2-	9.333353	9.333353	10.048971	8.62484
MB-7052	ER-/HER2-	10.310075	10.310075	10.215303	9.888685
MB-7054	ER-/HER2-	10.28566	10.28566	9.580309	9.074855
MB-7078	ER-/HER2-	11.362399	11.362399	9.90044	8.671424
MB-7079	ER-/HER2-	10.007809	10.007809	9.818406	8.818048
MB-7081	ER-/HER2-	11.495996	11.495996	10.44742	10.068962
MB-7084	ER-/HER2-	9.071054	9.071054	9.263275	9.585405
MB-7089	ER-/HER2-	10.634402	10.634402	9.674535	9.098991
MB-7090	ER-/HER2-	10.068962	10.068962	9.50405	9.94504
MB-7104	ER-/HER2-	10.981373	10.981373	9.950289	10.068962
MB-7114	ER-/HER2-	11.13867	11.13867	9.580988	8.974021
MB-7119	ER-/HER2-	10.450171	10.450171	10.288194	10.067075
MB-7121	ER-/HER2-	10.654395	10.654395	9.787465	10.034288
MB-7145	ER-/HER2-	9.96933	9.96933	8.432587	8.967237
MB-7151	ER-/HER2-	9.987552	9.987552	10.625016	9.985646
MB-7154	ER-/HER2-	11.264521	11.264521	10.202325	9.228381
MB-7155	ER-/HER2-	10.171703	10.171703	9.404886	9.520859
MB-7158	ER-/HER2-	11.079117	11.079117	10.570396	10.486336
MB-7165	ER-/HER2-	11.584475	11.584475	10.170011	9.252758
MB-7201	ER-/HER2-	9.269746	9.269746	9.073888	9.151322
MB-7205	ER-/HER2-	11.202807	11.202807	8.830203	9.737642
MB-7207	ER-/HER2-	10.452949	10.452949	8.740936	9.627071
MB-7208	ER-/HER2-	10.444788	10.444788	10.930495	10.09775
MB-7252	ER-/HER2-	10.627945	10.627945	10.049056	8.812287
MB-7269	ER-/HER2-	10.40958	10.40958	8.169403	9.258969
MB-7270	ER-/HER2-	9.724671	9.724671	10.718405	9.186847
MB-0002	ER+/HER2- Hi	9.547659		9.438209	10.339967
MB-0008	ER+/HER2- Hi	10.999749		8.767017	9.915887
MB-0010	ER+/HER2- Hi	10.552298		9.608828	8.747291
MB-0028	ER+/HER2- Hi	9.506558		8.568954	9.723196
MB-0035	ER+/HER2- Hi	7.929349		9.338938	9.699686
MB-0060	ER+/HER2- Hi	11.526585		11.030687	9.743829
MB-0066	ER+/HER2- Hi	11.022788		9.88458	9.4224
MB-0102	ER+/HER2- Hi	10.202884		10.131211	9.544187
MB-0109	ER+/HER2- Hi	10.141794		9.485197	9.490213
MB-0114	ER+/HER2- Hi	8.865289		9.519441	10.279084

MB-0116	ER+/HER2- Hi	9.443071	8.378173	9.667544
MB-0119	ER+/HER2- Hi	10.122889	9.849363	9.162192
MB-0120	ER+/HER2- Hi	10.753348	10.774016	8.95068
MB-0123	ER+/HER2- Hi	10.110437	9.177275	10.223754
MB-0131	ER+/HER2- Hi	10.565411	10.58288	9.542333
MB-0134	ER+/HER2- Hi	11.270627	8.327717	9.636414
MB-0135	ER+/HER2- Hi	9.434573	10.220852	8.786059
MB-0140	ER+/HER2- Hi	9.234262	10.110437	8.418506
MB-0143	ER+/HER2- Hi	9.738255	10.485172	9.547333
MB-0146	ER+/HER2- Hi	9.696775	10.468868	10.42355
MB-0147	ER+/HER2- Hi	9.422678	9.513934	9.021122
MB-0151	ER+/HER2- Hi	10.336715	9.509297	10.348224
MB-0167	ER+/HER2- Hi	11.11603	9.916515	9.706549
MB-0173	ER+/HER2- Hi	8.924406	8.874066	8.869582
MB-0176	ER+/HER2- Hi	9.967956	9.034026	9.310313
MB-0193	ER+/HER2- Hi	9.874404	9.805403	10.234367
MB-0195	ER+/HER2- Hi	9.975526	10.845479	9.889431
MB-0197	ER+/HER2- Hi	11.132973	9.842528	9.375437
MB-0198	ER+/HER2- Hi	9.891977	9.474406	10.47099
MB-0202	ER+/HER2- Hi	10.618041	10.081931	9.510148
MB-0203	ER+/HER2- Hi	10.042043	8.761791	10.049322
MB-0215	ER+/HER2- Hi	9.797694	10.840018	9.52458
MB-0218	ER+/HER2- Hi	10.563777	9.772573	9.100401
MB-0221	ER+/HER2- Hi	10.347866	10.401498	9.056369
MB-0234	ER+/HER2- Hi	10.753313	10.336995	9.565558
MB-0257	ER+/HER2- Hi	9.566125	10.439118	9.04484
MB-0258	ER+/HER2- Hi	10.399223	10.383261	8.610515
MB-0261	ER+/HER2- Hi	10.277674	9.810845	8.575001
MB-0270	ER+/HER2- Hi	10.171126	10.972754	9.961235
MB-0272	ER+/HER2- Hi	9.822406	9.980922	9.157954
MB-0287	ER+/HER2- Hi	9.684751	10.285736	9.524105
MB-0306	ER+/HER2- Hi	10.289918	10.144814	9.41444
MB-0311	ER+/HER2- Hi	9.492757	10.514372	9.801045
MB-0312	ER+/HER2- Hi	10.188584	9.932538	9.019288
MB-0313	ER+/HER2- Hi	11.16494	9.935524	9.946123
MB-0319	ER+/HER2- Hi	9.957136	10.932484	10.144279
MB-0321	ER+/HER2- Hi	10.345693	10.337936	9.250626
MB-0324	ER+/HER2- Hi	10.08963	10.026678	9.707896
MB-0325	ER+/HER2- Hi	10.57486	9.057845	9.066456
MB-0328	ER+/HER2- Hi	10.699423	10.208355	10.334788
MB-0336	ER+/HER2- Hi	10.66984	11.535129	9.009112
MB-0341	ER+/HER2- Hi	11.323513	9.725262	9.509068
MB-0349	ER+/HER2- Hi	10.070066	9.305218	9.29593

MB-0356	ER+/HER2- Hi	10.612029	8.454935	9.881033
MB-0358	ER+/HER2- Hi	10.356447	10.521492	9.254827
MB-0360	ER+/HER2- Hi	9.762292	10.621526	8.869999
MB-0362	ER+/HER2- Hi	10.543753	10.425399	9.293344
MB-0363	ER+/HER2- Hi	10.112312	10.029773	9.76168
MB-0366	ER+/HER2- Hi	9.32004	10.032692	9.823756
MB-0368	ER+/HER2- Hi	9.695369	9.51855	9.900549
MB-0370	ER+/HER2- Hi	11.422731	10.826269	9.571946
MB-0374	ER+/HER2- Hi	10.933062	9.510358	9.112289
MB-0380	ER+/HER2- Hi	10.310597	10.109421	10.637407
MB-0383	ER+/HER2- Hi	11.369163	11.10891	10.566041
MB-0384	ER+/HER2- Hi	9.855314	10.08887	10.045474
MB-0385	ER+/HER2- Hi	10.297466	10.375333	10.135135
MB-0392	ER+/HER2- Hi	10.489395	8.818498	9.916428
MB-0393	ER+/HER2- Hi	10.631111	10.421483	10.722176
MB-0394	ER+/HER2- Hi	10.290009	10.622187	9.78073
MB-0398	ER+/HER2- Hi	10.987813	10.926066	9.188335
MB-0404	ER+/HER2- Hi	10.47478	10.31512	9.745686
MB-0406	ER+/HER2- Hi	10.670114	9.844972	9.755165
MB-0412	ER+/HER2- Hi	11.077654	10.770471	9.771101
MB-0413	ER+/HER2- Hi	10.585144	9.895499	9.747814
MB-0417	ER+/HER2- Hi	10.228296	10.038889	9.204714
MB-0418	ER+/HER2- Hi	9.928551	10.061259	8.935681
MB-0427	ER+/HER2- Hi	10.251026	9.19503	10.474596
MB-0428	ER+/HER2- Hi	10.728044	9.742585	9.787818
MB-0429	ER+/HER2- Hi	10.70983	10.246159	10.270399
MB-0437	ER+/HER2- Hi	9.696846	7.682237	10.124522
MB-0439	ER+/HER2- Hi	9.938067	9.588155	9.783001
MB-0440	ER+/HER2- Hi	10.585666	9.474097	9.7647
MB-0445	ER+/HER2- Hi	10.029035	9.790399	10.02664
MB-0452	ER+/HER2- Hi	10.950702	10.225744	9.967028
MB-0453	ER+/HER2- Hi	10.490129	9.738493	8.634643
MB-0455	ER+/HER2- Hi	10.30841	10.339947	11.294018
MB-0459	ER+/HER2- Hi	10.71285	10.548363	8.466168
MB-0466	ER+/HER2- Hi	9.885376	9.873933	9.599196
MB-0469	ER+/HER2- Hi	9.881116	9.913084	9.913587
MB-0471	ER+/HER2- Hi	9.576476	9.355666	9.534993
MB-0472	ER+/HER2- Hi	8.231978	10.125178	9.060598
MB-0474	ER+/HER2- Hi	11.090749	9.568203	11.103373
MB-0475	ER+/HER2- Hi	11.691638	10.092895	9.636153
MB-0483	ER+/HER2- Hi	9.835181	10.754532	9.089106
MB-0484	ER+/HER2- Hi	10.223879	9.200702	9.998519
MB-0485	ER+/HER2- Hi	10.515899	9.989353	10.436268

MB-0491	ER+/HER2- Hi	10.718162	10.387976	9.314667
MB-0492	ER+/HER2- Hi	8.590884	8.411298	10.071856
MB-0506	ER+/HER2- Hi	11.262337	10.20907	8.989206
MB-0508	ER+/HER2- Hi	10.959649	10.471543	10.425193
MB-0509	ER+/HER2- Hi	10.221605	10.634402	11.090829
MB-0510	ER+/HER2- Hi	10.084621	10.515767	9.88614
MB-0514	ER+/HER2- Hi	11.005528	10.870966	10.398778
MB-0526	ER+/HER2- Hi	10.312562	9.736541	9.846332
MB-0529	ER+/HER2- Hi	9.830043	8.987087	9.658945
MB-0532	ER+/HER2- Hi	10.909235	9.692462	8.928105
MB-0536	ER+/HER2- Hi	11.482617	10.619306	9.716232
MB-0538	ER+/HER2- Hi	10.880764	8.85373	9.78282
MB-0541	ER+/HER2- Hi	10.386421	10.628101	9.760556
MB-0542	ER+/HER2- Hi	10.952986	9.906217	10.774016
MB-0545	ER+/HER2- Hi	9.948472	9.01318	8.750457
MB-0550	ER+/HER2- Hi	10.884623	10.092151	9.904492
MB-0559	ER+/HER2- Hi	10.193621	10.288335	9.235222
MB-0569	ER+/HER2- Hi	10.776987	10.295122	10.518743
MB-0570	ER+/HER2- Hi	10.084621	9.790079	9.56811
MB-0571	ER+/HER2- Hi	10.340787	10.285076	9.876943
MB-0574	ER+/HER2- Hi	9.870296	9.99214	10.132271
MB-0576	ER+/HER2- Hi	10.170401	9.592517	10.186338
MB-0577	ER+/HER2- Hi	9.887792	8.559224	9.578638
MB-0580	ER+/HER2- Hi	10.55669	10.310075	9.558052
MB-0584	ER+/HER2- Hi	9.953782	10.184309	9.47326
MB-0585	ER+/HER2- Hi	10.475517	10.26831	9.874458
MB-0587	ER+/HER2- Hi	10.57615	9.1128	9.726104
MB-0589	ER+/HER2- Hi	10.357116	9.608338	9.926782
MB-0590	ER+/HER2- Hi	10.618762	9.791678	9.650979
MB-0591	ER+/HER2- Hi	9.998216	10.426814	10.096817
MB-0594	ER+/HER2- Hi	10.630074	11.064107	9.844643
MB-0598	ER+/HER2- Hi	10.984364	10.254551	9.390483
MB-0600	ER+/HER2- Hi	9.491863	9.799344	8.63677
MB-0601	ER+/HER2- Hi	12.328106	10.006093	9.505946
MB-0606	ER+/HER2- Hi	9.873587	9.714857	10.3052
MB-0607	ER+/HER2- Hi	10.162255	9.635692	9.612408
MB-0609	ER+/HER2- Hi	10.46665	9.885538	12.021988
MB-0611	ER+/HER2- Hi	12.113253	9.401422	9.975523
MB-0614	ER+/HER2- Hi	10.553531	9.130551	10.128601
MB-0616	ER+/HER2- Hi	10.677173	9.840572	10.301362
MB-0619	ER+/HER2- Hi	10.332572	8.951218	10.618141
MB-0628	ER+/HER2- Hi	10.588242	11.020882	9.853677
MB-0630	ER+/HER2- Hi	10.855039	11.02749	9.879117

MB-0636	ER+/HER2- Hi	10.518093	9.863299	9.987552
MB-0637	ER+/HER2- Hi	11.332967	9.487662	9.818285
MB-0650	ER+/HER2- Hi	10.164934	9.74954	9.773098
MB-0653	ER+/HER2- Hi	11.185635	10.451833	9.598782
MB-0660	ER+/HER2- Hi	10.866678	9.020286	10.972775
MB-0666	ER+/HER2- Hi	10.087042	11.170258	9.318586
MB-0877	ER+/HER2- Hi	11.381911	9.736541	9.703691
MB-0882	ER+/HER2- Hi	10.31267	10.028989	9.48742
MB-0884	ER+/HER2- Hi	10.150266	12.087213	10.041863
MB-0899	ER+/HER2- Hi	10.492213	9.963137	9.766937
MB-2613	ER+/HER2- Hi	10.774559	10.23288	9.317004
MB-2617	ER+/HER2- Hi	10.73327	10.699423	9.22678
MB-2634	ER+/HER2- Hi	10.208735	10.125178	9.247523
MB-2642	ER+/HER2- Hi	10.856988	11.288644	9.921802
MB-2686	ER+/HER2- Hi	11.214849	9.982371	8.806701
MB-2705	ER+/HER2- Hi	10.139469	8.822194	9.525949
MB-2708	ER+/HER2- Hi	11.116939	9.665475	9.305753
MB-2728	ER+/HER2- Hi	11.335501	9.783853	10.201744
MB-2730	ER+/HER2- Hi	10.502135	10.656584	9.753623
MB-2745	ER+/HER2- Hi	9.219458	9.515713	9.702063
MB-2763	ER+/HER2- Hi	10.220687	9.034289	10.080678
MB-2767	ER+/HER2- Hi	10.983071	9.36065	9.40146
MB-2769	ER+/HER2- Hi	9.80593	9.707301	9.134644
MB-2772	ER+/HER2- Hi	10.913534	11.076482	8.978969
MB-2774	ER+/HER2- Hi	10.836907	9.646727	9.670733
MB-2778	ER+/HER2- Hi	10.533266	9.587516	9.62292
MB-2779	ER+/HER2- Hi	10.476738	9.765207	9.763823
MB-2781	ER+/HER2- Hi	10.410637	9.297842	9.991486
MB-2790	ER+/HER2- Hi	10.217419	9.883105	8.726129
MB-2792	ER+/HER2- Hi	11.831332	9.801194	10.05519
MB-2793	ER+/HER2- Hi	9.820064	8.723151	10.217532
MB-2796	ER+/HER2- Hi	10.82905	9.107872	9.466998
MB-2797	ER+/HER2- Hi	9.886553	8.595234	10.564076
MB-2801	ER+/HER2- Hi	10.71427	11.294018	9.017479
MB-2803	ER+/HER2- Hi	10.757216	8.485354	9.847622
MB-2814	ER+/HER2- Hi	10.073692	9.248599	8.603569
MB-2838	ER+/HER2- Hi	10.272735	8.50507	9.754737
MB-2853	ER+/HER2- Hi	10.371788	9.708437	9.095724
MB-2858	ER+/HER2- Hi	10.960089	11.170258	10.102215
MB-2922	ER+/HER2- Hi	11.192296	9.301403	9.529577
MB-2927	ER+/HER2- Hi	9.836235	11.213632	9.382135
MB-2932	ER+/HER2- Hi	9.569249	10.065147	9.942626
MB-2951	ER+/HER2- Hi	10.269779	11.252821	9.512149

MB-2953	ER+/HER2- Hi	11.178631	11.170258	10.412285
MB-2954	ER+/HER2- Hi	9.814134	10.067075	9.660797
MB-2966	ER+/HER2- Hi	10.545742	8.416548	9.480732
MB-2999	ER+/HER2- Hi	10.644094	9.666427	10.177267
MB-3002	ER+/HER2- Hi	9.883739	9.946123	9.848468
MB-3007	ER+/HER2- Hi	10.870941	9.660797	9.365488
MB-3016	ER+/HER2- Hi	10.053443	8.528205	9.642774
MB-3021	ER+/HER2- Hi	9.534605	10.999152	9.808678
MB-3026	ER+/HER2- Hi	10.295023	10.197217	8.993981
MB-3028	ER+/HER2- Hi	10.463738	10.011495	9.753664
MB-3031	ER+/HER2- Hi	11.022124	9.978371	9.097226
MB-3050	ER+/HER2- Hi	9.20946	9.801045	9.115354
MB-3060	ER+/HER2- Hi	10.237812	8.796929	9.535465
MB-3083	ER+/HER2- Hi	9.467922	8.729173	10.327848
MB-3092	ER+/HER2- Hi	9.786938	11.079117	9.971765
MB-3102	ER+/HER2- Hi	10.061312	9.106869	9.887792
MB-3104	ER+/HER2- Hi	9.194672	10.894777	7.738643
MB-3167	ER+/HER2- Hi	9.673011	9.794878	9.1798
MB-3235	ER+/HER2- Hi	11.159855	9.876034	9.97548
MB-3253	ER+/HER2- Hi	10.955333	10.428076	10.255614
MB-3266	ER+/HER2- Hi	10.813882	10.401532	9.074854
MB-3275	ER+/HER2- Hi	11.135016	8.726129	9.521187
MB-3300	ER+/HER2- Hi	10.829266	9.404974	10.098612
MB-3301	ER+/HER2- Hi	9.844617	9.391906	9.006443
MB-3303	ER+/HER2- Hi	12.00193	9.594481	9.824895
MB-3341	ER+/HER2- Hi	9.74177	10.723907	11.128651
MB-3350	ER+/HER2- Hi	8.85381	10.329749	10.644286
MB-3357	ER+/HER2- Hi	9.784068	9.771554	9.299187
MB-3371	ER+/HER2- Hi	9.312336	9.058717	9.750208
MB-3388	ER+/HER2- Hi	10.103668	9.433496	10.322285
MB-3389	ER+/HER2- Hi	10.269515	10.090787	10.360132
MB-3402	ER+/HER2- Hi	9.96759	10.05149	9.906217
MB-3417	ER+/HER2- Hi	8.789726	10.032347	9.759308
MB-3430	ER+/HER2- Hi	9.498478	10.903102	9.937245
MB-3437	ER+/HER2- Hi	7.748055	10.787103	9.475086
MB-3459	ER+/HER2- Hi	10.680713	10.937517	9.859712
MB-3466	ER+/HER2- Hi	10.841415	10.455735	9.805741
MB-3487	ER+/HER2- Hi	10.206586	11.017725	10.23715
MB-3490	ER+/HER2- Hi	10.225224	9.397939	9.747158
MB-3492	ER+/HER2- Hi	10.795348	9.40146	10.264475
MB-3506	ER+/HER2- Hi	10.533097	10.903102	9.935524
MB-3525	ER+/HER2- Hi	11.285105	10.791061	9.702629
MB-3530	ER+/HER2- Hi	10.195007	10.116955	9.655162

MB-3536	ER+/HER2- Hi	10.806226	10.86155	10.464401
MB-3548	ER+/HER2- Hi	10.263226	10.212643	10.664681
MB-3556	ER+/HER2- Hi	11.122977	10.825536	10.667921
MB-3576	ER+/HER2- Hi	10.845303	10.295432	10.651045
MB-3600	ER+/HER2- Hi	11.05089	10.536184	10.324691
MB-3614	ER+/HER2- Hi	10.455735	9.985646	10.985642
MB-3754	ER+/HER2- Hi	11.180725	10.414855	10.691786
MB-3797	ER+/HER2- Hi	9.495184	10.452949	10.178049
MB-3824	ER+/HER2- Hi	11.244862	10.57615	10.221605
MB-3838	ER+/HER2- Hi	9.284261	10.716583	8.97295
MB-3840	ER+/HER2- Hi	10.012508	10.44742	9.863077
MB-3842	ER+/HER2- Hi	10.548715	10.877762	10.472889
MB-3852	ER+/HER2- Hi	10.195222	10.068962	11.113464
MB-4001	ER+/HER2- Hi	9.302515	9.223064	10.250741
MB-4003	ER+/HER2- Hi	10.163356	10.798615	10.327199
MB-4004	ER+/HER2- Hi	10.075656	10.015211	9.372292
MB-4011	ER+/HER2- Hi	9.283509	9.557299	9.799519
MB-4017	ER+/HER2- Hi	9.545085	10.624761	10.203914
MB-4046	ER+/HER2- Hi	8.687667	11.191686	9.765207
MB-4139	ER+/HER2- Hi	8.494219	9.646727	10.539198
MB-4148	ER+/HER2- Hi	9.727588	9.128519	9.771554
MB-4154	ER+/HER2- Hi	10.589242	10.067075	10.396062
MB-4171	ER+/HER2- Hi	9.673849	9.773098	9.487662
MB-4233	ER+/HER2- Hi	10.458988	9.88458	10.241565
MB-4236	ER+/HER2- Hi	8.766415	10.102796	10.070904
MB-4264	ER+/HER2- Hi	9.593148	9.218785	9.531217
MB-4266	ER+/HER2- Hi	10.049322	9.505328	10.250741
MB-4274	ER+/HER2- Hi	9.793267	9.833055	9.087353
MB-4281	ER+/HER2- Hi	9.837925	7.599629	9.247523
MB-4289	ER+/HER2- Hi	9.102069	9.570582	9.162534
MB-4310	ER+/HER2- Hi	6.675191	8.961307	10.009701
MB-4323	ER+/HER2- Hi	9.766705	10.143807	10.139679
MB-4421	ER+/HER2- Hi	9.116332	10.398778	10.070904
MB-4618	ER+/HER2- Hi	10.423616	9.854689	10.004105
MB-4623	ER+/HER2- Hi	10.468201	10.123136	10.192883
MB-4627	ER+/HER2- Hi	9.818406	10.372869	10.055333
MB-4630	ER+/HER2- Hi	9.486446	9.594481	9.414341
MB-4634	ER+/HER2- Hi	9.978838	10.011495	8.740168
MB-4639	ER+/HER2- Hi	9.841281	7.741961	10.44742
MB-4640	ER+/HER2- Hi	10.048971	10.55774	10.77591
MB-4642	ER+/HER2- Hi	10.215303	9.129552	9.980147
MB-4648	ER+/HER2- Hi	9.580309	9.522033	9.794878
MB-4649	ER+/HER2- Hi	9.90044	9.492617	8.490775

MB-4651	ER+/HER2- Hi	9.818406	9.683707	9.558644
MB-4654	ER+/HER2- Hi	10.44742	9.19919	10.401532
MB-4655	ER+/HER2- Hi	9.263275	10.664681	9.683707
MB-4666	ER+/HER2- Hi	9.674535	9.591889	10.385477
MB-4669	ER+/HER2- Hi	9.50405	10.269114	10.44742
MB-4671	ER+/HER2- Hi	9.950289	9.701156	10.104828
MB-4672	ER+/HER2- Hi	9.580988	9.738092	9.810548
MB-4673	ER+/HER2- Hi	10.288194	9.954827	9.574617
MB-4682	ER+/HER2- Hi	9.787465	10.171703	8.368728
MB-4685	ER+/HER2- Hi	8.432587	10.080678	10.365286
MB-4687	ER+/HER2- Hi	10.625016	9.741103	10.034288
MB-4702	ER+/HER2- Hi	10.202325	10.123136	10.310075
MB-4712	ER+/HER2- Hi	9.404886	10.461474	10.342501
MB-4723	ER+/HER2- Hi	10.570396	10.214947	9.864778
MB-4737	ER+/HER2- Hi	10.170011	8.468221	9.98942
MB-4746	ER+/HER2- Hi	9.073888	10.924427	10.357604
MB-4749	ER+/HER2- Hi	8.830203	9.940854	10.024777
MB-4750	ER+/HER2- Hi	8.740936	10.188504	10.228296
MB-4767	ER+/HER2- Hi	10.930495	10.020886	9.996726
MB-4787	ER+/HER2- Hi	10.049056	10.657896	10.119061
MB-4790	ER+/HER2- Hi	8.169403	10.108865	8.773691
MB-4791	ER+/HER2- Hi	10.718405	9.894345	9.62606
MB-4794	ER+/HER2- Hi	9.438209	9.262218	10.444788
MB-4797	ER+/HER2- Hi	8.767017	9.78858	9.765207
MB-4800	ER+/HER2- Hi	9.608828	10.611648	9.965748
MB-4801	ER+/HER2- Hi	8.568954	9.482622	9.428819
MB-4802	ER+/HER2- Hi	9.338938	9.068206	10.15232
MB-4805	ER+/HER2- Hi	11.030687	9.009178	9.807303
MB-4829	ER+/HER2- Hi	9.88458	8.771222	9.523349
MB-4834	ER+/HER2- Hi	10.131211	10.946556	9.733558
MB-4836	ER+/HER2- Hi	9.485197	10.71285	9.159409
MB-4839	ER+/HER2- Hi	9.519441	10.573184	10.024777
MB-4849	ER+/HER2- Hi	8.378173	9.563948	9.635893
MB-4851	ER+/HER2- Hi	9.849363	8.748895	8.813915
MB-4862	ER+/HER2- Hi	10.774016	9.88614	9.105881
MB-4894	ER+/HER2- Hi	9.177275	9.05311	9.092173
MB-4900	ER+/HER2- Hi	10.58288	9.507845	10.627945
MB-4906	ER+/HER2- Hi	8.327717	9.020211	9.452844
MB-4912	ER+/HER2- Hi	10.220852	10.036232	10.412285
MB-4933	ER+/HER2- Hi	10.110437	11.036171	9.314667
MB-4934	ER+/HER2- Hi	10.485172	9.98197	10.436454
MB-4937	ER+/HER2- Hi	10.468868	10.684588	9.61381
MB-4944	ER+/HER2- Hi	9.513934	10.104828	9.926782

MB-4956	ER+/HER2- Hi	9.509297	10.417476	9.066309
MB-4965	ER+/HER2- Hi	9.916515	11.329883	9.225241
MB-4969	ER+/HER2- Hi	8.874066		10.049582
MB-4970	ER+/HER2- Hi	9.034026		9.186847
MB-4986	ER+/HER2- Hi	9.805403		9.833055
MB-4991	ER+/HER2- Hi	10.845479		9.98197
MB-4994	ER+/HER2- Hi	9.842528		10.108865
MB-4998	ER+/HER2- Hi	9.474406		10.4983
MB-5001	ER+/HER2- Hi	10.081931		8.154238
MB-5014	ER+/HER2- Hi	8.761791		10.108865
MB-5017	ER+/HER2- Hi	10.840018		9.107872
MB-5018	ER+/HER2- Hi	9.772573		9.926782
MB-5033	ER+/HER2- Hi	10.401498		9.316855
MB-5035	ER+/HER2- Hi	10.336995		10.481299
MB-5039	ER+/HER2- Hi	10.439118		10.614937
MB-5040	ER+/HER2- Hi	10.383261		9.99301
MB-5044	ER+/HER2- Hi	9.810845		10.742071
MB-5045	ER+/HER2- Hi	10.972754		10.545305
MB-5048	ER+/HER2- Hi	9.980922		
MB-5049	ER+/HER2- Hi	10.285736		
MB-5060	ER+/HER2- Hi	10.144814		
MB-5061	ER+/HER2- Hi	10.514372		
MB-5074	ER+/HER2- Hi	9.932538		
MB-5081	ER+/HER2- Hi	9.935524		
MB-5093	ER+/HER2- Hi	10.932484		
MB-5097	ER+/HER2- Hi	10.337936		
MB-5098	ER+/HER2- Hi	10.026678		
MB-5101	ER+/HER2- Hi	9.057845		
MB-5105	ER+/HER2- Hi	10.208355		
MB-5107	ER+/HER2- Hi	11.535129		
MB-5116	ER+/HER2- Hi	9.725262		
MB-5117	ER+/HER2- Hi	9.305218		
MB-5121	ER+/HER2- Hi	8.454935		
MB-5124	ER+/HER2- Hi	10.521492		
MB-5127	ER+/HER2- Hi	10.621526		
MB-5130	ER+/HER2- Hi	10.425399		
MB-5139	ER+/HER2- Hi	10.029773		
MB-5144	ER+/HER2- Hi	10.032692		
MB-5152	ER+/HER2- Hi	9.51855		
MB-5160	ER+/HER2- Hi	10.826269		
MB-5167	ER+/HER2- Hi	9.510358		
MB-5169	ER+/HER2- Hi	10.109421		
MB-5176	ER+/HER2- Hi	11.10891		

MB-5177	ER+/HER2- Hi	10.08887
MB-5186	ER+/HER2- Hi	10.375333
MB-5193	ER+/HER2- Hi	8.818498
MB-5196	ER+/HER2- Hi	10.421483
MB-5197	ER+/HER2- Hi	10.622187
MB-5200	ER+/HER2- Hi	10.926066
MB-5211	ER+/HER2- Hi	10.31512
MB-5215	ER+/HER2- Hi	9.844972
MB-5218	ER+/HER2- Hi	10.770471
MB-5226	ER+/HER2- Hi	9.895499
MB-5227	ER+/HER2- Hi	10.038889
MB-5228	ER+/HER2- Hi	10.061259
MB-5233	ER+/HER2- Hi	9.19503
MB-5239	ER+/HER2- Hi	9.742585
MB-5243	ER+/HER2- Hi	10.246159
MB-5244	ER+/HER2- Hi	7.682237
MB-5256	ER+/HER2- Hi	9.588155
MB-5260	ER+/HER2- Hi	9.474097
MB-5261	ER+/HER2- Hi	9.790399
MB-5266	ER+/HER2- Hi	10.225744
MB-5270	ER+/HER2- Hi	9.738493
MB-5271	ER+/HER2- Hi	10.339947
MB-5272	ER+/HER2- Hi	10.548363
MB-5273	ER+/HER2- Hi	9.873933
MB-5275	ER+/HER2- Hi	9.913084
MB-5279	ER+/HER2- Hi	9.355666
MB-5280	ER+/HER2- Hi	10.125178
MB-5288	ER+/HER2- Hi	9.568203
MB-5291	ER+/HER2- Hi	10.092895
MB-5292	ER+/HER2- Hi	10.754532
MB-5305	ER+/HER2- Hi	9.200702
MB-5306	ER+/HER2- Hi	9.989353
MB-5313	ER+/HER2- Hi	10.387976
MB-5317	ER+/HER2- Hi	8.411298
MB-5322	ER+/HER2- Hi	10.20907
MB-5328	ER+/HER2- Hi	10.471543
MB-5332	ER+/HER2- Hi	10.634402
MB-5334	ER+/HER2- Hi	10.515767
MB-5338	ER+/HER2- Hi	10.870966
MB-5339	ER+/HER2- Hi	9.736541
MB-5341	ER+/HER2- Hi	8.987087
MB-5347	ER+/HER2- Hi	9.692462
MB-5361	ER+/HER2- Hi	10.619306

MB-5370	ER+/HER2- Hi	8.85373
MB-5384	ER+/HER2- Hi	10.628101
MB-5386	ER+/HER2- Hi	9.906217
MB-5389	ER+/HER2- Hi	9.01318
MB-5396	ER+/HER2- Hi	10.092151
MB-5397	ER+/HER2- Hi	10.288335
MB-5402	ER+/HER2- Hi	10.295122
MB-5404	ER+/HER2- Hi	9.790079
MB-5405	ER+/HER2- Hi	10.285076
MB-5432	ER+/HER2- Hi	9.99214
MB-5433	ER+/HER2- Hi	9.592517
MB-5434	ER+/HER2- Hi	8.559224
MB-5435	ER+/HER2- Hi	10.310075
MB-5447	ER+/HER2- Hi	10.184309
MB-5454	ER+/HER2- Hi	10.26831
MB-5463	ER+/HER2- Hi	9.1128
MB-5470	ER+/HER2- Hi	9.608338
MB-5471	ER+/HER2- Hi	9.791678
MB-5477	ER+/HER2- Hi	10.426814
MB-5481	ER+/HER2- Hi	11.064107
MB-5485	ER+/HER2- Hi	10.254551
MB-5486	ER+/HER2- Hi	9.799344
MB-5491	ER+/HER2- Hi	10.006093
MB-5493	ER+/HER2- Hi	9.714857
MB-5497	ER+/HER2- Hi	9.635692
MB-5502	ER+/HER2- Hi	9.885538
MB-5505	ER+/HER2- Hi	9.401422
MB-5513	ER+/HER2- Hi	9.130551
MB-5518	ER+/HER2- Hi	9.840572
MB-5519	ER+/HER2- Hi	8.951218
MB-5520	ER+/HER2- Hi	11.020882
MB-5521	ER+/HER2- Hi	11.02749
MB-5525	ER+/HER2- Hi	9.863299
MB-5530	ER+/HER2- Hi	9.487662
MB-5532	ER+/HER2- Hi	9.74954
MB-5540	ER+/HER2- Hi	10.451833
MB-5550	ER+/HER2- Hi	9.020286
MB-5552	ER+/HER2- Hi	11.170258
MB-5553	ER+/HER2- Hi	9.736541
MB-5554	ER+/HER2- Hi	10.028989
MB-5556	ER+/HER2- Hi	12.087213
MB-5562	ER+/HER2- Hi	9.963137
MB-5575	ER+/HER2- Hi	10.23288

MB-5576	ER+/HER2- Hi	10.699423
MB-5577	ER+/HER2- Hi	10.125178
MB-5584	ER+/HER2- Hi	11.288644
MB-5590	ER+/HER2- Hi	9.982371
MB-5592	ER+/HER2- Hi	8.822194
MB-5604	ER+/HER2- Hi	9.665475
MB-5605	ER+/HER2- Hi	9.783853
MB-5613	ER+/HER2- Hi	10.656584
MB-5622	ER+/HER2- Hi	9.515713
MB-5623	ER+/HER2- Hi	9.034289
MB-5628	ER+/HER2- Hi	9.36065
MB-5629	ER+/HER2- Hi	9.707301
MB-5632	ER+/HER2- Hi	11.076482
MB-5636	ER+/HER2- Hi	9.646727
MB-5638	ER+/HER2- Hi	9.587516
MB-5641	ER+/HER2- Hi	9.765207
MB-5646	ER+/HER2- Hi	9.297842
MB-5647	ER+/HER2- Hi	9.883105
MB-5653	ER+/HER2- Hi	9.801194
MB-5654	ER+/HER2- Hi	8.723151
MB-6007	ER+/HER2- Hi	9.107872
MB-6008	ER+/HER2- Hi	8.595234
MB-6010	ER+/HER2- Hi	11.294018
MB-6011	ER+/HER2- Hi	8.485354
MB-6022	ER+/HER2- Hi	9.248599
MB-6023	ER+/HER2- Hi	8.50507
MB-6026	ER+/HER2- Hi	9.708437
MB-6039	ER+/HER2- Hi	11.170258
MB-6044	ER+/HER2- Hi	9.301403
MB-6047	ER+/HER2- Hi	11.213632
MB-6053	ER+/HER2- Hi	10.065147
MB-6059	ER+/HER2- Hi	11.252821
MB-6060	ER+/HER2- Hi	11.170258
MB-6071	ER+/HER2- Hi	10.067075
MB-6075	ER+/HER2- Hi	8.416548
MB-6077	ER+/HER2- Hi	9.666427
MB-6079	ER+/HER2- Hi	9.946123
MB-6080	ER+/HER2- Hi	9.660797
MB-6097	ER+/HER2- Hi	8.528205
MB-6105	ER+/HER2- Hi	10.999152
MB-6114	ER+/HER2- Hi	10.197217
MB-6124	ER+/HER2- Hi	10.011495
MB-6133	ER+/HER2- Hi	9.978371

MB-6135	ER+/HER2- Hi	9.801045
MB-6141	ER+/HER2- Hi	8.796929
MB-6145	ER+/HER2- Hi	8.729173
MB-6146	ER+/HER2- Hi	11.079117
MB-6149	ER+/HER2- Hi	9.106869
MB-6150	ER+/HER2- Hi	10.894777
MB-6154	ER+/HER2- Hi	9.794878
MB-6163	ER+/HER2- Hi	9.876034
MB-6164	ER+/HER2- Hi	10.428076
MB-6179	ER+/HER2- Hi	10.401532
MB-6181	ER+/HER2- Hi	8.726129
MB-6183	ER+/HER2- Hi	9.404974
MB-6184	ER+/HER2- Hi	9.391906
MB-6189	ER+/HER2- Hi	9.594481
MB-6192	ER+/HER2- Hi	10.723907
MB-6200	ER+/HER2- Hi	10.329749
MB-6201	ER+/HER2- Hi	9.771554
MB-6208	ER+/HER2- Hi	9.058717
MB-6217	ER+/HER2- Hi	9.433496
MB-6226	ER+/HER2- Hi	10.090787
MB-6239	ER+/HER2- Hi	10.05149
MB-6257	ER+/HER2- Hi	10.032347
MB-6302	ER+/HER2- Hi	10.903102
MB-6306	ER+/HER2- Hi	10.787103
MB-6312	ER+/HER2- Hi	10.937517
MB-6317	ER+/HER2- Hi	10.455735
MB-6322	ER+/HER2- Hi	11.017725
MB-6327	ER+/HER2- Hi	9.397939
MB-6328	ER+/HER2- Hi	9.40146
MB-6329	ER+/HER2- Hi	10.903102
MB-6344	ER+/HER2- Hi	10.791061
MB-6346	ER+/HER2- Hi	10.116955
MB-6359	ER+/HER2- Hi	10.86155
MB-7000	ER+/HER2- Hi	10.212643
MB-7002	ER+/HER2- Hi	10.825536
MB-7011	ER+/HER2- Hi	10.295432
MB-7014	ER+/HER2- Hi	10.536184
MB-7015	ER+/HER2- Hi	9.985646
MB-7018	ER+/HER2- Hi	10.414855
MB-7022	ER+/HER2- Hi	10.452949
MB-7024	ER+/HER2- Hi	10.57615
MB-7028	ER+/HER2- Hi	10.716583
MB-7042	ER+/HER2- Hi	10.44742

MB-7046	ER+/HER2- Hi	10.877762
MB-7048	ER+/HER2- Hi	10.068962
MB-7050	ER+/HER2- Hi	9.223064
MB-7051	ER+/HER2- Hi	10.798615
MB-7053	ER+/HER2- Hi	10.015211
MB-7065	ER+/HER2- Hi	9.557299
MB-7070	ER+/HER2- Hi	10.624761
MB-7071	ER+/HER2- Hi	11.191686
MB-7072	ER+/HER2- Hi	9.646727
MB-7075	ER+/HER2- Hi	9.128519
MB-7083	ER+/HER2- Hi	10.067075
MB-7086	ER+/HER2- Hi	9.773098
MB-7092	ER+/HER2- Hi	9.88458
MB-7094	ER+/HER2- Hi	10.102796
MB-7095	ER+/HER2- Hi	9.218785
MB-7097	ER+/HER2- Hi	9.505328
MB-7099	ER+/HER2- Hi	9.833055
MB-7100	ER+/HER2- Hi	7.599629
MB-7102	ER+/HER2- Hi	9.570582
MB-7107	ER+/HER2- Hi	8.961307
MB-7109	ER+/HER2- Hi	10.143807
MB-7111	ER+/HER2- Hi	10.398778
MB-7112	ER+/HER2- Hi	9.854689
MB-7116	ER+/HER2- Hi	10.123136
MB-7122	ER+/HER2- Hi	10.372869
MB-7124	ER+/HER2- Hi	9.594481
MB-7127	ER+/HER2- Hi	10.011495
MB-7131	ER+/HER2- Hi	7.741961
MB-7133	ER+/HER2- Hi	10.55774
MB-7138	ER+/HER2- Hi	9.129552
MB-7140	ER+/HER2- Hi	9.522033
MB-7142	ER+/HER2- Hi	9.492617
MB-7144	ER+/HER2- Hi	9.683707
MB-7147	ER+/HER2- Hi	9.19919
MB-7149	ER+/HER2- Hi	10.664681
MB-7150	ER+/HER2- Hi	9.591889
MB-7152	ER+/HER2- Hi	10.269114
MB-7153	ER+/HER2- Hi	9.701156
MB-7157	ER+/HER2- Hi	9.738092
MB-7160	ER+/HER2- Hi	9.954827
MB-7161	ER+/HER2- Hi	10.171703
MB-7171	ER+/HER2- Hi	10.080678
MB-7172	ER+/HER2- Hi	9.741103

MB-7173	ER+/HER2- Hi	10.123136
MB-7174	ER+/HER2- Hi	10.461474
MB-7176	ER+/HER2- Hi	10.214947
MB-7181	ER+/HER2- Hi	8.468221
MB-7182	ER+/HER2- Hi	10.924427
MB-7186	ER+/HER2- Hi	9.940854
MB-7193	ER+/HER2- Hi	10.188504
MB-7194	ER+/HER2- Hi	10.020886
MB-7196	ER+/HER2- Hi	10.657896
MB-7197	ER+/HER2- Hi	10.108865
MB-7198	ER+/HER2- Hi	9.894345
MB-7199	ER+/HER2- Hi	9.262218
MB-7200	ER+/HER2- Hi	9.78858
MB-7226	ER+/HER2- Hi	10.611648
MB-7227	ER+/HER2- Hi	9.482622
MB-7229	ER+/HER2- Hi	9.068206
MB-7230	ER+/HER2- Hi	9.009178
MB-7234	ER+/HER2- Hi	8.771222
MB-7237	ER+/HER2- Hi	10.946556
MB-7238	ER+/HER2- Hi	10.71285
MB-7253	ER+/HER2- Hi	10.573184
MB-7254	ER+/HER2- Hi	9.563948
MB-7262	ER+/HER2- Hi	8.748895
MB-7263	ER+/HER2- Hi	9.88614
MB-7268	ER+/HER2- Hi	9.05311
MB-7280	ER+/HER2- Hi	9.507845
MB-7283	ER+/HER2- Hi	9.020211
MB-7286	ER+/HER2- Hi	10.036232
MB-7287	ER+/HER2- Hi	11.036171
MB-7288	ER+/HER2- Hi	9.98197
MB-7292	ER+/HER2- Hi	10.684588
MB-7294	ER+/HER2- Hi	10.104828
MB-7298	ER+/HER2- Hi	10.417476
MB-7299	ER+/HER2- Hi	11.329883
MB-0036	ER+/HER2- Lc	10.570197
MB-0050	ER+/HER2- Lc	9.662606
MB-0101	ER+/HER2- Lc	10.669429
MB-0106	ER+/HER2- Lc	9.566815
MB-0111	ER+/HER2- Lc	9.559906
MB-0112	ER+/HER2- Lc	9.837235
MB-0117	ER+/HER2- Lc	10.163683
MB-0121	ER+/HER2- Lc	9.89235
MB-0122	ER+/HER2- Lc	10.049783

MB-0124	ER+/HER2- Lc	10.180187
MB-0125	ER+/HER2- Lc	9.813701
MB-0126	ER+/HER2- Lc	9.837119
MB-0127	ER+/HER2- Lc	10.455735
MB-0128	ER+/HER2- Lc	10.560879
MB-0133	ER+/HER2- Lc	10.006762
MB-0136	ER+/HER2- Lc	11.250565
MB-0138	ER+/HER2- Lc	9.785267
MB-0139	ER+/HER2- Lc	9.329582
MB-0142	ER+/HER2- Lc	10.285775
MB-0144	ER+/HER2- Lc	9.742329
MB-0145	ER+/HER2- Lc	9.316393
MB-0154	ER+/HER2- Lc	10.617633
MB-0155	ER+/HER2- Lc	10.770026
MB-0162	ER+/HER2- Lc	9.590881
MB-0166	ER+/HER2- Lc	10.092974
MB-0168	ER+/HER2- Lc	10.920228
MB-0170	ER+/HER2- Lc	10.551519
MB-0171	ER+/HER2- Lc	10.093199
MB-0172	ER+/HER2- Lc	10.112902
MB-0175	ER+/HER2- Lc	10.554601
MB-0177	ER+/HER2- Lc	10.495371
MB-0180	ER+/HER2- Lc	10.749492
MB-0181	ER+/HER2- Lc	9.732535
MB-0184	ER+/HER2- Lc	9.43
MB-0189	ER+/HER2- Lc	10.508641
MB-0192	ER+/HER2- Lc	10.398645
MB-0194	ER+/HER2- Lc	10.345083
MB-0199	ER+/HER2- Lc	10.313197
MB-0204	ER+/HER2- Lc	10.227911
MB-0205	ER+/HER2- Lc	9.326726
MB-0207	ER+/HER2- Lc	10.417907
MB-0211	ER+/HER2- Lc	11.682904
MB-0222	ER+/HER2- Lc	10.406776
MB-0223	ER+/HER2- Lc	10.608847
MB-0224	ER+/HER2- Lc	10.488611
MB-0226	ER+/HER2- Lc	9.78858
MB-0227	ER+/HER2- Lc	10.219419
MB-0228	ER+/HER2- Lc	9.907967
MB-0229	ER+/HER2- Lc	10.11634
MB-0231	ER+/HER2- Lc	9.427589
MB-0232	ER+/HER2- Lc	9.825336
MB-0233	ER+/HER2- Lc	10.608432

MB-0235	ER+/HER2- Lc	10.865556
MB-0239	ER+/HER2- Lc	11.254058
MB-0242	ER+/HER2- Lc	10.760923
MB-0243	ER+/HER2- Lc	10.437545
MB-0245	ER+/HER2- Lc	9.938955
MB-0247	ER+/HER2- Lc	10.561095
MB-0248	ER+/HER2- Lc	8.699085
MB-0253	ER+/HER2- Lc	9.186847
MB-0260	ER+/HER2- Lc	9.897777
MB-0263	ER+/HER2- Lc	10.206151
MB-0264	ER+/HER2- Lc	10.994763
MB-0273	ER+/HER2- Lc	9.105898
MB-0275	ER+/HER2- Lc	10.772019
MB-0279	ER+/HER2- Lc	11.050314
MB-0280	ER+/HER2- Lc	9.386074
MB-0282	ER+/HER2- Lc	10.533266
MB-0288	ER+/HER2- Lc	10.681164
MB-0290	ER+/HER2- Lc	10.143807
MB-0295	ER+/HER2- Lc	10.750216
MB-0301	ER+/HER2- Lc	10.334814
MB-0302	ER+/HER2- Lc	10.193443
MB-0305	ER+/HER2- Lc	10.444788
MB-0308	ER+/HER2- Lc	9.751725
MB-0309	ER+/HER2- Lc	9.249606
MB-0310	ER+/HER2- Lc	10.46528
MB-0315	ER+/HER2- Lc	10.510495
MB-0317	ER+/HER2- Lc	9.986185
MB-0320	ER+/HER2- Lc	10.393312
MB-0322	ER+/HER2- Lc	10.630074
MB-0339	ER+/HER2- Lc	10.045829
MB-0342	ER+/HER2- Lc	9.96933
MB-0344	ER+/HER2- Lc	10.050351
MB-0345	ER+/HER2- Lc	9.383528
MB-0348	ER+/HER2- Lc	9.136009
MB-0353	ER+/HER2- Lc	10.28566
MB-0359	ER+/HER2- Lc	10.090195
MB-0364	ER+/HER2- Lc	10.488611
MB-0377	ER+/HER2- Lc	10.919344
MB-0379	ER+/HER2- Lc	10.608193
MB-0382	ER+/HER2- Lc	10.202325
MB-0386	ER+/HER2- Lc	10.713464
MB-0388	ER+/HER2- Lc	10.560879
MB-0397	ER+/HER2- Lc	10.705165

MB-0402	ER+/HER2- Lc	10.506879
MB-0410	ER+/HER2- Lc	9.910066
MB-0411	ER+/HER2- Lc	10.551204
MB-0419	ER+/HER2- Lc	10.014486
MB-0422	ER+/HER2- Lc	10.320227
MB-0425	ER+/HER2- Lc	10.867442
MB-0426	ER+/HER2- Lc	9.576055
MB-0431	ER+/HER2- Lc	10.399087
MB-0442	ER+/HER2- Lc	10.458573
MB-0443	ER+/HER2- Lc	10.294823
MB-0444	ER+/HER2- Lc	10.48741
MB-0449	ER+/HER2- Lc	9.731953
MB-0451	ER+/HER2- Lc	9.987574
MB-0454	ER+/HER2- Lc	8.916544
MB-0458	ER+/HER2- Lc	10.235016
MB-0460	ER+/HER2- Lc	10.778496
MB-0463	ER+/HER2- Lc	10.084892
MB-0478	ER+/HER2- Lc	10.503827
MB-0480	ER+/HER2- Lc	9.834416
MB-0486	ER+/HER2- Lc	9.550583
MB-0487	ER+/HER2- Lc	10.57615
MB-0490	ER+/HER2- Lc	9.834634
MB-0496	ER+/HER2- Lc	10.148086
MB-0497	ER+/HER2- Lc	10.006823
MB-0501	ER+/HER2- Lc	9.566934
MB-0503	ER+/HER2- Lc	9.995978
MB-0504	ER+/HER2- Lc	10.323682
MB-0505	ER+/HER2- Lc	10.039767
MB-0507	ER+/HER2- Lc	10.685609
MB-0511	ER+/HER2- Lc	9.989254
MB-0513	ER+/HER2- Lc	9.252758
MB-0517	ER+/HER2- Lc	9.535026
MB-0519	ER+/HER2- Lc	10.001456
MB-0521	ER+/HER2- Lc	10.059602
MB-0524	ER+/HER2- Lc	10.246159
MB-0527	ER+/HER2- Lc	10.109503
MB-0528	ER+/HER2- Lc	10.749492
MB-0535	ER+/HER2- Lc	9.089322
MB-0537	ER+/HER2- Lc	10.853439
MB-0544	ER+/HER2- Lc	10.156708
MB-0551	ER+/HER2- Lc	9.958468
MB-0554	ER+/HER2- Lc	10.86155
MB-0568	ER+/HER2- Lc	10.068721

MB-0573	ER+/HER2- Lc	11.253395
MB-0575	ER+/HER2- Lc	11.201717
MB-0578	ER+/HER2- Lc	10.139679
MB-0579	ER+/HER2- Lc	10.280644
MB-0583	ER+/HER2- Lc	9.386512
MB-0586	ER+/HER2- Lc	10.757172
MB-0588	ER+/HER2- Lc	10.637569
MB-0596	ER+/HER2- Lc	10.472116
MB-0597	ER+/HER2- Lc	10.465635
MB-0599	ER+/HER2- Lc	10.193621
MB-0603	ER+/HER2- Lc	10.064987
MB-0605	ER+/HER2- Lc	10.286129
MB-0610	ER+/HER2- Lc	11.238632
MB-0618	ER+/HER2- Lc	10.48045
MB-0621	ER+/HER2- Lc	10.519396
MB-0624	ER+/HER2- Lc	9.892084
MB-0631	ER+/HER2- Lc	10.025902
MB-0632	ER+/HER2- Lc	10.76539
MB-0640	ER+/HER2- Lc	9.802586
MB-0641	ER+/HER2- Lc	10.523995
MB-0642	ER+/HER2- Lc	9.047628
MB-0644	ER+/HER2- Lc	10.880764
MB-0649	ER+/HER2- Lc	10.560227
MB-0654	ER+/HER2- Lc	8.942652
MB-0655	ER+/HER2- Lc	11.664224
MB-0657	ER+/HER2- Lc	10.659624
MB-0661	ER+/HER2- Lc	9.650979
MB-0872	ER+/HER2- Lc	9.882834
MB-0880	ER+/HER2- Lc	10.669429
MB-0891	ER+/HER2- Lc	10.200736
MB-0897	ER+/HER2- Lc	9.976605
MB-0904	ER+/HER2- Lc	10.033348
MB-2536	ER+/HER2- Lc	10.21116
MB-2564	ER+/HER2- Lc	10.122201
MB-2610	ER+/HER2- Lc	10.030499
MB-2614	ER+/HER2- Lc	10.597752
MB-2616	ER+/HER2- Lc	10.533962
MB-2624	ER+/HER2- Lc	10.603106
MB-2669	ER+/HER2- Lc	11.117205
MB-2711	ER+/HER2- Lc	10.811984
MB-2712	ER+/HER2- Lc	10.554601
MB-2725	ER+/HER2- Lc	10.149005
MB-2744	ER+/HER2- Lc	9.310152

MB-2747	ER+/HER2- Lc	9.68821
MB-2749	ER+/HER2- Lc	10.385423
MB-2750	ER+/HER2- Lc	9.624664
MB-2752	ER+/HER2- Lc	9.968118
MB-2754	ER+/HER2- Lc	9.663305
MB-2760	ER+/HER2- Lc	10.224875
MB-2765	ER+/HER2- Lc	11.245259
MB-2770	ER+/HER2- Lc	9.416736
MB-2791	ER+/HER2- Lc	9.864731
MB-2795	ER+/HER2- Lc	10.928108
MB-2815	ER+/HER2- Lc	9.492617
MB-2819	ER+/HER2- Lc	10.152228
MB-2823	ER+/HER2- Lc	10.317343
MB-2835	ER+/HER2- Lc	10.031701
MB-2840	ER+/HER2- Lc	9.970353
MB-2843	ER+/HER2- Lc	11.053074
MB-2845	ER+/HER2- Lc	10.51989
MB-2848	ER+/HER2- Lc	10.639973
MB-2851	ER+/HER2- Lc	10.909512
MB-2854	ER+/HER2- Lc	9.21355
MB-2863	ER+/HER2- Lc	10.342623
MB-2867	ER+/HER2- Lc	10.231122
MB-2896	ER+/HER2- Lc	11.323974
MB-2900	ER+/HER2- Lc	9.894345
MB-2916	ER+/HER2- Lc	9.895777
MB-2919	ER+/HER2- Lc	10.123136
MB-2931	ER+/HER2- Lc	11.055087
MB-2933	ER+/HER2- Lc	9.741261
MB-2944	ER+/HER2- Lc	10.239395
MB-2947	ER+/HER2- Lc	10.179469
MB-2960	ER+/HER2- Lc	9.938109
MB-2969	ER+/HER2- Lc	10.582653
MB-2970	ER+/HER2- Lc	10.643377
MB-2971	ER+/HER2- Lc	10.545637
MB-2977	ER+/HER2- Lc	10.389998
MB-2990	ER+/HER2- Lc	9.288294
MB-2996	ER+/HER2- Lc	10.366495
MB-3005	ER+/HER2- Lc	10.398645
MB-3008	ER+/HER2- Lc	10.299855
MB-3013	ER+/HER2- Lc	11.11603
MB-3032	ER+/HER2- Lc	10.508641
MB-3033	ER+/HER2- Lc	9.749271
MB-3035	ER+/HER2- Lc	9.97868

MB-3037	ER+/HER2- Lc	9.801963
MB-3049	ER+/HER2- Lc	9.947863
MB-3064	ER+/HER2- Lc	10.261234
MB-3079	ER+/HER2- Lc	9.472007
MB-3085	ER+/HER2- Lc	10.528281
MB-3105	ER+/HER2- Lc	10.30253
MB-3110	ER+/HER2- Lc	10.598514
MB-3121	ER+/HER2- Lc	9.682177
MB-3171	ER+/HER2- Lc	9.897905
MB-3222	ER+/HER2- Lc	10.805337
MB-3228	ER+/HER2- Lc	10.118699
MB-3252	ER+/HER2- Lc	10.182271
MB-3254	ER+/HER2- Lc	10.423638
MB-3295	ER+/HER2- Lc	9.234834
MB-3298	ER+/HER2- Lc	10.215849
MB-3328	ER+/HER2- Lc	10.476674
MB-3351	ER+/HER2- Lc	10.168946
MB-3365	ER+/HER2- Lc	9.351939
MB-3378	ER+/HER2- Lc	10.021795
MB-3381	ER+/HER2- Lc	10.059205
MB-3403	ER+/HER2- Lc	10.643377
MB-3412	ER+/HER2- Lc	10.382571
MB-3429	ER+/HER2- Lc	9.754423
MB-3439	ER+/HER2- Lc	9.938067
MB-3450	ER+/HER2- Lc	9.659054
MB-3452	ER+/HER2- Lc	11.177765
MB-3462	ER+/HER2- Lc	9.656502
MB-3510	ER+/HER2- Lc	9.408491
MB-3526	ER+/HER2- Lc	11.003741
MB-3545	ER+/HER2- Lc	9.718927
MB-3707	ER+/HER2- Lc	10.817394
MB-3711	ER+/HER2- Lc	10.038889
MB-3748	ER+/HER2- Lc	10.52463
MB-3781	ER+/HER2- Lc	9.880672
MB-3850	ER+/HER2- Lc	10.552947
MB-3854	ER+/HER2- Lc	9.819122
MB-3865	ER+/HER2- Lc	10.056903
MB-3871	ER+/HER2- Lc	10.312842
MB-3874	ER+/HER2- Lc	11.1206
MB-4000	ER+/HER2- Lc	10.215303
MB-4012	ER+/HER2- Lc	8.62484
MB-4018	ER+/HER2- Lc	9.888685
MB-4120	ER+/HER2- Lc	9.074855

MB-4140	ER+/HER2- Lc	8.671424
MB-4141	ER+/HER2- Lc	8.818048
MB-4145	ER+/HER2- Lc	10.068962
MB-4173	ER+/HER2- Lc	9.585405
MB-4212	ER+/HER2- Lc	9.098991
MB-4230	ER+/HER2- Lc	9.94504
MB-4283	ER+/HER2- Lc	10.068962
MB-4293	ER+/HER2- Lc	8.974021
MB-4342	ER+/HER2- Lc	10.067075
MB-4343	ER+/HER2- Lc	10.034288
MB-4357	ER+/HER2- Lc	8.967237
MB-4529	ER+/HER2- Lc	9.985646
MB-4599	ER+/HER2- Lc	9.228381
MB-4616	ER+/HER2- Lc	9.520859
MB-4633	ER+/HER2- Lc	10.486336
MB-4641	ER+/HER2- Lc	9.252758
MB-4653	ER+/HER2- Lc	9.151322
MB-4661	ER+/HER2- Lc	9.737642
MB-4665	ER+/HER2- Lc	9.627071
MB-4670	ER+/HER2- Lc	10.09775
MB-4674	ER+/HER2- Lc	8.812287
MB-4675	ER+/HER2- Lc	9.258969
MB-4681	ER+/HER2- Lc	9.186847
MB-4686	ER+/HER2- Lc	10.339967
MB-4691	ER+/HER2- Lc	9.915887
MB-4692	ER+/HER2- Lc	8.747291
MB-4695	ER+/HER2- Lc	9.723196
MB-4697	ER+/HER2- Lc	9.699686
MB-4698	ER+/HER2- Lc	9.743829
MB-4701	ER+/HER2- Lc	9.4224
MB-4704	ER+/HER2- Lc	9.544187
MB-4705	ER+/HER2- Lc	9.490213
MB-4706	ER+/HER2- Lc	10.279084
MB-4708	ER+/HER2- Lc	9.667544
MB-4709	ER+/HER2- Lc	9.162192
MB-4710	ER+/HER2- Lc	8.95068
MB-4716	ER+/HER2- Lc	10.223754
MB-4718	ER+/HER2- Lc	9.542333
MB-4719	ER+/HER2- Lc	9.636414
MB-4721	ER+/HER2- Lc	8.786059
MB-4722	ER+/HER2- Lc	8.418506
MB-4730	ER+/HER2- Lc	9.547333
MB-4735	ER+/HER2- Lc	10.42355

MB-4738	ER+/HER2- Lc	9.021122
MB-4739	ER+/HER2- Lc	10.348224
MB-4741	ER+/HER2- Lc	9.706549
MB-4742	ER+/HER2- Lc	8.869582
MB-4743	ER+/HER2- Lc	9.310313
MB-4752	ER+/HER2- Lc	10.234367
MB-4760	ER+/HER2- Lc	9.889431
MB-4762	ER+/HER2- Lc	9.375437
MB-4764	ER+/HER2- Lc	10.47099
MB-4771	ER+/HER2- Lc	9.510148
MB-4778	ER+/HER2- Lc	10.049322
MB-4779	ER+/HER2- Lc	9.52458
MB-4784	ER+/HER2- Lc	9.100401
MB-4785	ER+/HER2- Lc	9.056369
MB-4806	ER+/HER2- Lc	9.565558
MB-4814	ER+/HER2- Lc	9.04484
MB-4818	ER+/HER2- Lc	8.610515
MB-4820	ER+/HER2- Lc	8.575001
MB-4822	ER+/HER2- Lc	9.961235
MB-4825	ER+/HER2- Lc	9.157954
MB-4827	ER+/HER2- Lc	9.524105
MB-4832	ER+/HER2- Lc	9.41444
MB-4843	ER+/HER2- Lc	9.801045
MB-4845	ER+/HER2- Lc	9.019288
MB-4853	ER+/HER2- Lc	9.946123
MB-4855	ER+/HER2- Lc	10.144279
MB-4860	ER+/HER2- Lc	9.250626
MB-4867	ER+/HER2- Lc	9.707896
MB-4869	ER+/HER2- Lc	9.066456
MB-4870	ER+/HER2- Lc	10.334788
MB-4872	ER+/HER2- Lc	9.009112
MB-4882	ER+/HER2- Lc	9.509068
MB-4883	ER+/HER2- Lc	9.29593
MB-4887	ER+/HER2- Lc	9.881033
MB-4897	ER+/HER2- Lc	9.254827
MB-4898	ER+/HER2- Lc	8.869999
MB-4899	ER+/HER2- Lc	9.293344
MB-4941	ER+/HER2- Lc	9.76168
MB-4950	ER+/HER2- Lc	9.823756
MB-4959	ER+/HER2- Lc	9.900549
MB-4961	ER+/HER2- Lc	9.571946
MB-4962	ER+/HER2- Lc	9.112289
MB-4966	ER+/HER2- Lc	10.637407

MB-4967	ER+/HER2- Lc	10.566041
MB-4968	ER+/HER2- Lc	10.045474
MB-4977	ER+/HER2- Lc	10.135135
MB-4978	ER+/HER2- Lc	9.916428
MB-4981	ER+/HER2- Lc	10.722176
MB-4987	ER+/HER2- Lc	9.78073
MB-4992	ER+/HER2- Lc	9.188335
MB-4996	ER+/HER2- Lc	9.745686
MB-4999	ER+/HER2- Lc	9.755165
MB-5004	ER+/HER2- Lc	9.771101
MB-5011	ER+/HER2- Lc	9.747814
MB-5013	ER+/HER2- Lc	9.204714
MB-5015	ER+/HER2- Lc	8.935681
MB-5020	ER+/HER2- Lc	10.474596
MB-5027	ER+/HER2- Lc	9.787818
MB-5043	ER+/HER2- Lc	10.270399
MB-5050	ER+/HER2- Lc	10.124522
MB-5053	ER+/HER2- Lc	9.783001
MB-5059	ER+/HER2- Lc	9.7647
MB-5064	ER+/HER2- Lc	10.02664
MB-5066	ER+/HER2- Lc	9.967028
MB-5068	ER+/HER2- Lc	8.634643
MB-5073	ER+/HER2- Lc	11.294018
MB-5079	ER+/HER2- Lc	8.466168
MB-5084	ER+/HER2- Lc	9.599196
MB-5088	ER+/HER2- Lc	9.913587
MB-5092	ER+/HER2- Lc	9.534993
MB-5110	ER+/HER2- Lc	9.060598
MB-5113	ER+/HER2- Lc	11.103373
MB-5118	ER+/HER2- Lc	9.636153
MB-5119	ER+/HER2- Lc	9.089106
MB-5122	ER+/HER2- Lc	9.998519
MB-5123	ER+/HER2- Lc	10.436268
MB-5134	ER+/HER2- Lc	9.314667
MB-5143	ER+/HER2- Lc	10.071856
MB-5150	ER+/HER2- Lc	8.989206
MB-5161	ER+/HER2- Lc	10.425193
MB-5163	ER+/HER2- Lc	11.090829
MB-5171	ER+/HER2- Lc	9.88614
MB-5175	ER+/HER2- Lc	10.398778
MB-5178	ER+/HER2- Lc	9.846332
MB-5179	ER+/HER2- Lc	9.658945
MB-5182	ER+/HER2- Lc	8.928105

MB-5183	ER+/HER2- Lc	9.716232
MB-5184	ER+/HER2- Lc	9.78282
MB-5185	ER+/HER2- Lc	9.760556
MB-5189	ER+/HER2- Lc	10.774016
MB-5201	ER+/HER2- Lc	8.750457
MB-5204	ER+/HER2- Lc	9.904492
MB-5206	ER+/HER2- Lc	9.235222
MB-5214	ER+/HER2- Lc	10.518743
MB-5221	ER+/HER2- Lc	9.56811
MB-5224	ER+/HER2- Lc	9.876943
MB-5230	ER+/HER2- Lc	10.132271
MB-5240	ER+/HER2- Lc	10.186338
MB-5251	ER+/HER2- Lc	9.578638
MB-5253	ER+/HER2- Lc	9.558052
MB-5264	ER+/HER2- Lc	9.47326
MB-5267	ER+/HER2- Lc	9.874458
MB-5268	ER+/HER2- Lc	9.726104
MB-5277	ER+/HER2- Lc	9.926782
MB-5278	ER+/HER2- Lc	9.650979
MB-5281	ER+/HER2- Lc	10.096817
MB-5284	ER+/HER2- Lc	9.844643
MB-5287	ER+/HER2- Lc	9.390483
MB-5290	ER+/HER2- Lc	8.63677
MB-5293	ER+/HER2- Lc	9.505946
MB-5300	ER+/HER2- Lc	10.3052
MB-5308	ER+/HER2- Lc	9.612408
MB-5310	ER+/HER2- Lc	12.021988
MB-5324	ER+/HER2- Lc	9.975523
MB-5329	ER+/HER2- Lc	10.128601
MB-5330	ER+/HER2- Lc	10.301362
MB-5345	ER+/HER2- Lc	10.618141
MB-5358	ER+/HER2- Lc	9.853677
MB-5360	ER+/HER2- Lc	9.879117
MB-5364	ER+/HER2- Lc	9.987552
MB-5365	ER+/HER2- Lc	9.818285
MB-5368	ER+/HER2- Lc	9.773098
MB-5369	ER+/HER2- Lc	9.598782
MB-5373	ER+/HER2- Lc	10.972775
MB-5377	ER+/HER2- Lc	9.318586
MB-5382	ER+/HER2- Lc	9.703691
MB-5383	ER+/HER2- Lc	9.48742
MB-5388	ER+/HER2- Lc	10.041863
MB-5393	ER+/HER2- Lc	9.766937

MB-5395	ER+/HER2- Lc	9.317004
MB-5398	ER+/HER2- Lc	9.22678
MB-5399	ER+/HER2- Lc	9.247523
MB-5401	ER+/HER2- Lc	9.921802
MB-5403	ER+/HER2- Lc	8.806701
MB-5407	ER+/HER2- Lc	9.525949
MB-5410	ER+/HER2- Lc	9.305753
MB-5412	ER+/HER2- Lc	10.201744
MB-5422	ER+/HER2- Lc	9.753623
MB-5424	ER+/HER2- Lc	9.702063
MB-5425	ER+/HER2- Lc	10.080678
MB-5428	ER+/HER2- Lc	9.40146
MB-5429	ER+/HER2- Lc	9.134644
MB-5441	ER+/HER2- Lc	8.978969
MB-5444	ER+/HER2- Lc	9.670733
MB-5451	ER+/HER2- Lc	9.62292
MB-5455	ER+/HER2- Lc	9.763823
MB-5457	ER+/HER2- Lc	9.991486
MB-5464	ER+/HER2- Lc	8.726129
MB-5472	ER+/HER2- Lc	10.05519
MB-5473	ER+/HER2- Lc	10.217532
MB-5475	ER+/HER2- Lc	9.466998
MB-5478	ER+/HER2- Lc	10.564076
MB-5484	ER+/HER2- Lc	9.017479
MB-5489	ER+/HER2- Lc	9.847622
MB-5490	ER+/HER2- Lc	8.603569
MB-5492	ER+/HER2- Lc	9.754737
MB-5495	ER+/HER2- Lc	9.095724
MB-5499	ER+/HER2- Lc	10.102215
MB-5510	ER+/HER2- Lc	9.529577
MB-5514	ER+/HER2- Lc	9.382135
MB-5533	ER+/HER2- Lc	9.942626
MB-5541	ER+/HER2- Lc	9.512149
MB-5543	ER+/HER2- Lc	10.412285
MB-5563	ER+/HER2- Lc	9.660797
MB-5567	ER+/HER2- Lc	9.480732
MB-5571	ER+/HER2- Lc	10.177267
MB-5579	ER+/HER2- Lc	9.848468
MB-5580	ER+/HER2- Lc	9.365488
MB-5582	ER+/HER2- Lc	9.642774
MB-5583	ER+/HER2- Lc	9.808678
MB-5589	ER+/HER2- Lc	8.993981
MB-5591	ER+/HER2- Lc	9.753664

MB-5596	ER+/HER2- Lc	9.097226
MB-5597	ER+/HER2- Lc	9.115354
MB-5599	ER+/HER2- Lc	9.535465
MB-5601	ER+/HER2- Lc	10.327848
MB-5603	ER+/HER2- Lc	9.971765
MB-5614	ER+/HER2- Lc	9.887792
MB-5617	ER+/HER2- Lc	7.738643
MB-5620	ER+/HER2- Lc	9.1798
MB-5626	ER+/HER2- Lc	9.97548
MB-5635	ER+/HER2- Lc	10.255614
MB-5640	ER+/HER2- Lc	9.074854
MB-5642	ER+/HER2- Lc	9.521187
MB-5645	ER+/HER2- Lc	10.098612
MB-5656	ER+/HER2- Lc	9.006443
MB-6001	ER+/HER2- Lc	9.824895
MB-6006	ER+/HER2- Lc	11.128651
MB-6014	ER+/HER2- Lc	10.644286
MB-6016	ER+/HER2- Lc	9.299187
MB-6017	ER+/HER2- Lc	9.750208
MB-6018	ER+/HER2- Lc	10.322285
MB-6019	ER+/HER2- Lc	10.360132
MB-6021	ER+/HER2- Lc	9.906217
MB-6024	ER+/HER2- Lc	9.759308
MB-6029	ER+/HER2- Lc	9.937245
MB-6030	ER+/HER2- Lc	9.475086
MB-6042	ER+/HER2- Lc	9.859712
MB-6050	ER+/HER2- Lc	9.805741
MB-6051	ER+/HER2- Lc	10.23715
MB-6065	ER+/HER2- Lc	9.747158
MB-6069	ER+/HER2- Lc	10.264475
MB-6082	ER+/HER2- Lc	9.935524
MB-6083	ER+/HER2- Lc	9.702629
MB-6092	ER+/HER2- Lc	9.655162
MB-6103	ER+/HER2- Lc	10.464401
MB-6107	ER+/HER2- Lc	10.664681
MB-6108	ER+/HER2- Lc	10.667921
MB-6118	ER+/HER2- Lc	10.651045
MB-6125	ER+/HER2- Lc	10.324691
MB-6138	ER+/HER2- Lc	10.985642
MB-6147	ER+/HER2- Lc	10.691786
MB-6167	ER+/HER2- Lc	10.178049
MB-6168	ER+/HER2- Lc	10.221605
MB-6171	ER+/HER2- Lc	8.97295

MB-6185	ER+/HER2- Lc	9.863077
MB-6190	ER+/HER2- Lc	10.472889
MB-6194	ER+/HER2- Lc	11.113464
MB-6195	ER+/HER2- Lc	10.250741
MB-6204	ER+/HER2- Lc	10.327199
MB-6207	ER+/HER2- Lc	9.372292
MB-6211	ER+/HER2- Lc	9.799519
MB-6212	ER+/HER2- Lc	10.203914
MB-6214	ER+/HER2- Lc	9.765207
MB-6218	ER+/HER2- Lc	10.539198
MB-6225	ER+/HER2- Lc	9.771554
MB-6229	ER+/HER2- Lc	10.396062
MB-6231	ER+/HER2- Lc	9.487662
MB-6232	ER+/HER2- Lc	10.241565
MB-6233	ER+/HER2- Lc	10.070904
MB-6234	ER+/HER2- Lc	9.531217
MB-6238	ER+/HER2- Lc	10.250741
MB-6253	ER+/HER2- Lc	9.087353
MB-6254	ER+/HER2- Lc	9.247523
MB-6256	ER+/HER2- Lc	9.162534
MB-6308	ER+/HER2- Lc	10.009701
MB-6319	ER+/HER2- Lc	10.139679
MB-7003	ER+/HER2- Lc	10.070904
MB-7004	ER+/HER2- Lc	10.004105
MB-7005	ER+/HER2- Lc	10.192883
MB-7006	ER+/HER2- Lc	10.055333
MB-7010	ER+/HER2- Lc	9.414341
MB-7013	ER+/HER2- Lc	8.740168
MB-7019	ER+/HER2- Lc	10.44742
MB-7026	ER+/HER2- Lc	10.77591
MB-7029	ER+/HER2- Lc	9.980147
MB-7032	ER+/HER2- Lc	9.794878
MB-7034	ER+/HER2- Lc	8.490775
MB-7037	ER+/HER2- Lc	9.558644
MB-7040	ER+/HER2- Lc	10.401532
MB-7041	ER+/HER2- Lc	9.683707
MB-7043	ER+/HER2- Lc	10.385477
MB-7044	ER+/HER2- Lc	10.44742
MB-7056	ER+/HER2- Lc	10.104828
MB-7057	ER+/HER2- Lc	9.810548
MB-7058	ER+/HER2- Lc	9.574617
MB-7060	ER+/HER2- Lc	8.368728
MB-7061	ER+/HER2- Lc	10.365286

MB-7062	ER+/HER2- Lc	10.034288
MB-7063	ER+/HER2- Lc	10.310075
MB-7066	ER+/HER2- Lc	10.342501
MB-7074	ER+/HER2- Lc	9.864778
MB-7076	ER+/HER2- Lc	9.98942
MB-7077	ER+/HER2- Lc	10.357604
MB-7080	ER+/HER2- Lc	10.024777
MB-7085	ER+/HER2- Lc	10.228296
MB-7091	ER+/HER2- Lc	9.996726
MB-7093	ER+/HER2- Lc	10.119061
MB-7101	ER+/HER2- Lc	8.773691
MB-7106	ER+/HER2- Lc	9.62606
MB-7113	ER+/HER2- Lc	10.444788
MB-7118	ER+/HER2- Lc	9.765207
MB-7123	ER+/HER2- Lc	9.965748
MB-7132	ER+/HER2- Lc	9.428819
MB-7137	ER+/HER2- Lc	10.15232
MB-7141	ER+/HER2- Lc	9.807303
MB-7162	ER+/HER2- Lc	9.523349
MB-7163	ER+/HER2- Lc	9.733558
MB-7164	ER+/HER2- Lc	9.159409
MB-7167	ER+/HER2- Lc	10.024777
MB-7168	ER+/HER2- Lc	9.635893
MB-7185	ER+/HER2- Lc	8.813915
MB-7195	ER+/HER2- Lc	9.105881
MB-7212	ER+/HER2- Lc	9.092173
MB-7215	ER+/HER2- Lc	10.627945
MB-7216	ER+/HER2- Lc	9.452844
MB-7217	ER+/HER2- Lc	10.412285
MB-7218	ER+/HER2- Lc	9.314667
MB-7219	ER+/HER2- Lc	10.436454
MB-7220	ER+/HER2- Lc	9.61381
MB-7228	ER+/HER2- Lc	9.926782
MB-7231	ER+/HER2- Lc	9.066309
MB-7232	ER+/HER2- Lc	9.225241
MB-7233	ER+/HER2- Lc	10.049582
MB-7235	ER+/HER2- Lc	9.186847
MB-7236	ER+/HER2- Lc	9.833055
MB-7241	ER+/HER2- Lc	9.98197
MB-7243	ER+/HER2- Lc	10.108865
MB-7244	ER+/HER2- Lc	10.4983
MB-7249	ER+/HER2- Lc	8.154238
MB-7264	ER+/HER2- Lc	10.108865

MB-7276	ER+/HER2- Lc	9.107872
MB-7277	ER+/HER2- Lc	9.926782
MB-7278	ER+/HER2- Lc	9.316855
MB-7284	ER+/HER2- Lc	10.481299
MB-7285	ER+/HER2- Lc	10.614937
MB-7289	ER+/HER2- Lc	9.99301
MB-7293	ER+/HER2- Lc	10.742071
MB-7295	ER+/HER2- Lc	10.545305
MB-0113	HER2+	9.836235
MB-0129	HER2+	10.518743
MB-0130	HER2+	9.91812
MB-0148	HER2+	11.554759
MB-0152	HER2+	10.917124
MB-0165	HER2+	10.065828
MB-0201	HER2+	10.396565
MB-0220	HER2+	11.131192
MB-0225	HER2+	9.743075
MB-0230	HER2+	10.190647
MB-0236	HER2+	10.476674
MB-0291	HER2+	11.536425
MB-0294	HER2+	10.739265
MB-0307	HER2+	10.570396
MB-0314	HER2+	10.865805
MB-0346	HER2+	10.85482
MB-0361	HER2+	10.255614
MB-0371	HER2+	10.748513
MB-0373	HER2+	10.812001
MB-0378	HER2+	11.157669
MB-0381	HER2+	11.721069
MB-0389	HER2+	10.357106
MB-0395	HER2+	10.89947
MB-0421	HER2+	10.481299
MB-0434	HER2+	10.219533
MB-0438	HER2+	10.763251
MB-0462	HER2+	11.355797
MB-0465	HER2+	11.0589
MB-0467	HER2+	10.357947
MB-0479	HER2+	10.154484
MB-0482	HER2+	10.716826
MB-0552	HER2+	10.853439
MB-0593	HER2+	9.849539
MB-0615	HER2+	9.771554
MB-0656	HER2+	11.22653

MB-0662	HER2+	10.98416
MB-0663	HER2+	10.579241
MB-0895	HER2+	10.54845
MB-2513	HER2+	11.484326
MB-2517	HER2+	10.716583
MB-2626	HER2+	10.276219
MB-2632	HER2+	11.067877
MB-2735	HER2+	10.590635
MB-2742	HER2+	12.007138
MB-2758	HER2+	10.865556
MB-2786	HER2+	10.79883
MB-2844	HER2+	11.489996
MB-2847	HER2+	10.553531
MB-2895	HER2+	10.825536
MB-2923	HER2+	10.119061
MB-2964	HER2+	10.44913
MB-2983	HER2+	11.064107
MB-2984	HER2+	11.05767
MB-2994	HER2+	10.138829
MB-3025	HER2+	10.484264
MB-3103	HER2+	10.40136
MB-3122	HER2+	10.232351
MB-3272	HER2+	10.534123
MB-3329	HER2+	10.110127
MB-3355	HER2+	10.583186
MB-3360	HER2+	10.564816
MB-3361	HER2+	9.959144
MB-3379	HER2+	10.521737
MB-3382	HER2+	11.130313
MB-3386	HER2+	10.62811
MB-3435	HER2+	11.457901
MB-3467	HER2+	10.560164
MB-3470	HER2+	10.910782
MB-3488	HER2+	10.168665
MB-3497	HER2+	10.770103
MB-3528	HER2+	11.123457
MB-3606	HER2+	10.801815
MB-3866	HER2+	11.091739
MB-3978	HER2+	10.951151
MB-4127	HER2+	9.543852
MB-4270	HER2+	9.815312
MB-4276	HER2+	10.657109
MB-4643	HER2+	10.890474

MB-4644	HER2+	9.021118
MB-4663	HER2+	10.015211
MB-4688	HER2+	10.22631
MB-4724	HER2+	11.331098
MB-4725	HER2+	10.742071
MB-4729	HER2+	9.509063
MB-4731	HER2+	10.206151
MB-4745	HER2+	10.309681
MB-4763	HER2+	9.569711
MB-4796	HER2+	10.515873
MB-4846	HER2+	10.985642
MB-4858	HER2+	9.812333
MB-4866	HER2+	9.079124
MB-4871	HER2+	9.998182
MB-4878	HER2+	10.024885
MB-4879	HER2+	9.751725
MB-4886	HER2+	10.169088
MB-4896	HER2+	9.621949
MB-4904	HER2+	10.154484
MB-4908	HER2+	10.542541
MB-4929	HER2+	8.701007
MB-4930	HER2+	10.003627
MB-4935	HER2+	10.818993
MB-4952	HER2+	11.114969
MB-5019	HER2+	10.181646
MB-5054	HER2+	10.484117
MB-5062	HER2+	11.325723
MB-5063	HER2+	10.398778
MB-5078	HER2+	10.29736
MB-5114	HER2+	11.346656
MB-5120	HER2+	11.218548
MB-5147	HER2+	10.006242
MB-5164	HER2+	9.813701
MB-5166	HER2+	9.401212
MB-5172	HER2+	10.675784
MB-5174	HER2+	10.000402
MB-5195	HER2+	10.600954
MB-5199	HER2+	10.192883
MB-5212	HER2+	9.759401
MB-5213	HER2+	11.446284
MB-5229	HER2+	10.957949
MB-5231	HER2+	10.372869
MB-5238	HER2+	9.965944

MB-5259	HER2+	11.010692
MB-5296	HER2+	10.048606
MB-5312	HER2+	10.633807
MB-5315	HER2+	10.752245
MB-5318	HER2+	10.889101
MB-5327	HER2+	10.675459
MB-5331	HER2+	9.183848
MB-5351	HER2+	9.757383
MB-5366	HER2+	10.637609
MB-5381	HER2+	11.720234
MB-5409	HER2+	10.52826
MB-5411	HER2+	10.262935
MB-5414	HER2+	10.406637
MB-5417	HER2+	10.167445
MB-5418	HER2+	9.621658
MB-5420	HER2+	9.637249
MB-5426	HER2+	10.455735
MB-5458	HER2+	9.552107
MB-5459	HER2+	10.015119
MB-5474	HER2+	11.181745
MB-5483	HER2+	10.986615
MB-5498	HER2+	10.467398
MB-5535	HER2+	10.335827
MB-5558	HER2+	9.074854
MB-5593	HER2+	9.616473
MB-6048	HER2+	11.036171
MB-6049	HER2+	10.757172
MB-6063	HER2+	9.846332
MB-6113	HER2+	10.806226
MB-6116	HER2+	11.123457
MB-6131	HER2+	10.276219
MB-6156	HER2+	11.180725
MB-6157	HER2+	10.915953
MB-6160	HER2+	10.536184
MB-6182	HER2+	10.355109
MB-6246	HER2+	11.045562
MB-6314	HER2+	10.226054
MB-6330	HER2+	9.791678
MB-6334	HER2+	10.154484
MB-6337	HER2+	9.567897
MB-6358	HER2+	10.77591
MB-6363	HER2+	10.915953
MB-7001	HER2+	10.221605

MB-7020	HER2+	10.869591
MB-7027	HER2+	11.022124
MB-7035	HER2+	10.404188
MB-7059	HER2+	10.246133
MB-7067	HER2+	10.946556
MB-7068	HER2+	11.08883
MB-7069	HER2+	11.045562
MB-7073	HER2+	10.533266
MB-7082	HER2+	10.469987
MB-7088	HER2+	10.87385
MB-7115	HER2+	10.428076
MB-7128	HER2+	10.560879
MB-7135	HER2+	8.471587
MB-7143	HER2+	10.009701
MB-7187	HER2+	11.395064
MB-7250	HER2+	10.783331
MB-7251	HER2+	10.4983
MB-7256	HER2+	9.688088
MB-7260	HER2+	10.241565
MB-7273	HER2+	10.920228
MB-7275	HER2+	10.817736
MB-7279	HER2+	10.439118
MB-7281	HER2+	11.808207
MB-7291	HER2+	10.674698

HER2+
9.836235
10.518743
9.91812
11.554759
10.917124
10.065828
10.396565
11.131192
9.743075
10.190647
10.476674
11.536425
10.739265
10.570396
10.865805
10.85482
10.255614
10.748513
10.812001
11.157669
11.721069
10.357106
10.89947
10.481299
10.219533
10.763251
11.355797
11.0589
10.357947
10.154484
10.716826
10.853439
9.849539
9.771554
11.22653
10.98416
10.579241
10.54845
11.484326
10.716583
10.276219
11.067877

10.590635
12.007138
10.865556
10.79883
11.489996
10.553531
10.825536
10.119061
10.44913
11.064107
11.05767
10.138829
10.484264
10.40136
10.232351
10.534123
10.110127
10.583186
10.564816
9.959144
10.521737
11.130313
10.62811
11.457901
10.560164
10.910782
10.168665
10.770103
11.123457
10.801815
11.091739
10.951151
9.543852
9.815312
10.657109
10.890474
9.021118
10.015211
10.22631
11.331098
10.742071
9.509063
10.206151

10.309681
9.569711
10.515873
10.985642
9.812333
9.079124
9.998182
10.024885
9.751725
10.169088
9.621949
10.154484
10.542541
8.701007
10.003627
10.818993
11.114969
10.181646
10.484117
11.325723
10.398778
10.29736
11.346656
11.218548
10.006242
9.813701
9.401212
10.675784
10.000402
10.600954
10.192883
9.759401
11.446284
10.957949
10.372869
9.965944
11.010692
10.048606
10.633807
10.752245
10.889101
10.675459
9.183848

9.757383
10.637609
11.720234
10.52826
10.262935
10.406637
10.167445
9.621658
9.637249
10.455735
9.552107
10.015119
11.181745
10.986615
10.467398
10.335827
9.074854
9.616473
11.036171
10.757172
9.846332
10.806226
11.123457
10.276219
11.180725
10.915953
10.536184
10.355109
11.045562
10.226054
9.791678
10.154484
9.567897
10.77591
10.915953
10.221605
10.869591
11.022124
10.404188
10.246133
10.946556
11.08883
11.045562

10.533266
10.469987
10.87385
10.428076
10.560879
8.471587
10.009701
11.395064
10.783331
10.4983
9.688088
10.241565
10.920228
10.817736
10.439118
11.808207
10.674698

Anova: Single Factor

SUMMARY

Tukey treatment group	Groups	Count	Sum	Average	Variance
A	ER-/HER2-	290	3104.47942	10.7051014	0.57250063
B	ER+/HER- Hig	603	6076.02413	10.0763252	0.54532875
C	ER+/HER2- Lc	619	6155.69451	9.94457917	0.35830329
D	HER2+	188	1973.64483	10.4981108	0.35173811

ANOVA

Source of Variati	*SS*	*df*	*MS*	*F*	*P-value*
Between Gro	139.766154	3	46.5887181	101.17775	2.9823E-60
Within Group	780.947052	1696	0.46046406		
Total	920.713207	1699			

treatments	Tukey HSD Q statistic	Tukey HSD p-value	inferfence
A vs B	18.3377	0.0010053	** $p<0.01$
A vs C	22.2737	0.0010053	** $p<0.01$
A vs D	4.6071	0.006293	** $p<0.01$
B vs C	4.7987	0.0039394	** $p<0.01$
B vs D	10.5235	0.0010053	** $p<0.01$
C vs D	13.8531	0.0010053	** $p<0.01$

F crit
2.61014911

Sample Id	3-Gene classi	CUX1: mRNA expression (R	ER-/HER2-	ER+/HER- Hig	ER+/HER2- Lc
MB-0000	ER-/HER2-	6.806361	6.806361	7.716212	6.931262
MB-0100	ER-/HER2-	8.179472	8.179472	7.172573	6.774453
MB-0115	ER-/HER2-	7.066109	7.066109	7.080922	7.196093
MB-0149	ER-/HER2-	7.546003	7.546003	6.906297	7.324199
MB-0150	ER-/HER2-	7.508865	7.508865	7.799011	7.145237
MB-0157	ER-/HER2-	7.690211	7.690211	7.925118	6.841047
MB-0164	ER-/HER2-	7.726481	7.726481	7.538038	7.71145
MB-0174	ER-/HER2-	8.047802	8.047802	7.091365	6.73756
MB-0179	ER-/HER2-	7.410808	7.410808	7.912455	7.017889
MB-0206	ER-/HER2-	6.990305	6.990305	7.212006	7.608808
MB-0209	ER-/HER2-	7.441557	7.441557	7.048093	7.453552
MB-0214	ER-/HER2-	8.059992	8.059992	7.225232	7.370213
MB-0238	ER-/HER2-	7.377472	7.377472	6.512082	7.596713
MB-0249	ER-/HER2-	7.470481	7.470481	6.835758	7.57992
MB-0259	ER-/HER2-	8.465487	8.465487	7.109417	7.252236
MB-0265	ER-/HER2-	6.957803	6.957803	7.434772	7.379545
MB-0269	ER-/HER2-	7.591419	7.591419	7.541629	7.680957
MB-0278	ER-/HER2-	7.761472	7.761472	6.327295	7.288411
MB-0284	ER-/HER2-	7.869641	7.869641	7.965306	7.296535
MB-0285	ER-/HER2-	7.046663	7.046663	7.89807	7.906936
MB-0286	ER-/HER2-	7.194131	7.194131	7.538969	7.360992
MB-0289	ER-/HER2-	7.50258	7.50258	7.778228	7.003357
MB-0292	ER-/HER2-	7.627693	7.627693	7.554356	7.422553
MB-0293	ER-/HER2-	7.395034	7.395034	7.711101	7.2912
MB-0303	ER-/HER2-	7.737429	7.737429	7.36352	7.625829
MB-0316	ER-/HER2-	7.413356	7.413356	7.566647	7.498508
MB-0318	ER-/HER2-	8.644046	8.644046	7.278049	6.914093
MB-0340	ER-/HER2-	8.146683	8.146683	6.93273	7.304259
MB-0352	ER-/HER2-	7.812345	7.812345	7.269788	7.234934
MB-0354	ER-/HER2-	6.950591	6.950591	7.098351	7.743428
MB-0365	ER-/HER2-	7.670217	7.670217	7.079512	7.062087
MB-0372	ER-/HER2-	7.720885	7.720885	7.145187	7.855265
MB-0375	ER-/HER2-	6.769449	6.769449	7.601986	7.168911
MB-0396	ER-/HER2-	8.448073	8.448073	7.068848	7.055838
MB-0399	ER-/HER2-	7.399922	7.399922	7.490105	7.617278
MB-0400	ER-/HER2-	8.08727	8.08727	6.812535	6.918569
MB-0401	ER-/HER2-	8.065032	8.065032	7.364654	7.353478
MB-0414	ER-/HER2-	9.11078	9.11078	6.686828	7.127757
MB-0420	ER-/HER2-	7.950424	7.950424	6.705479	7.272283
MB-0424	ER-/HER2-	6.677859	6.677859	7.044099	7.075136

MB-0436	ER-/HER2-	7.490105		7.490105	7.109542	7.080089
MB-0446	ER-/HER2-	7.156705		7.156705	7.162324	7.43758
MB-0464	ER-/HER2-	7.488216		7.488216	7.722806	7.156941
MB-0476	ER-/HER2-	7.418324		7.418324	7.045967	7.707437
MB-0481	ER-/HER2-	7.005963		7.005963	6.943495	7.026796
MB-0488	ER-/HER2-	6.909796		6.909796	6.721924	7.490105
MB-0489	ER-/HER2-	7.950519		7.950519	7.134587	6.888481
MB-0494	ER-/HER2-	7.494027		7.494027	7.379266	7.064948
MB-0495	ER-/HER2-	7.457202		7.457202	7.761783	7.147766
MB-0500	ER-/HER2-	7.606278		7.606278	7.376044	7.31091
MB-0502	ER-/HER2-	7.734912		7.734912	7.356844	6.826292
MB-0516	ER-/HER2-	7.808889		7.808889	7.295405	7.27916
MB-0525	ER-/HER2-	7.450632		7.450632	6.739426	7.089083
MB-0540	ER-/HER2-	7.770574		7.770574	7.316642	6.852541
MB-0558	ER-/HER2-	7.812124		7.812124	7.084345	6.804085
MB-0581	ER-/HER2-	7.339509		7.339509	6.850091	6.883628
MB-0582	ER-/HER2-	6.519632		6.519632	7.257938	7.053883
MB-0608	ER-/HER2-	8.51463		8.51463	7.005793	7.039864
MB-0613	ER-/HER2-	7.924309		7.924309	7.413018	7.186006
MB-0617	ER-/HER2-	7.151103		7.151103	6.813402	7.078815
MB-0620	ER-/HER2-	6.71542		6.71542	7.406483	6.907464
MB-0627	ER-/HER2-	7.552121		7.552121	7.923486	6.896998
MB-0643	ER-/HER2-	7.282647		7.282647	6.866491	6.537326
MB-0658	ER-/HER2-	7.529053		7.529053	7.867488	6.762964
MB-0659	ER-/HER2-	6.820944		6.820944	7.455339	7.096081
MB-0664	ER-/HER2-	7.586421		7.586421	8.294131	6.983997
MB-0869	ER-/HER2-	7.908636		7.908636	7.613565	7.545538
MB-0874	ER-/HER2-	6.864567		6.864567	7.726851	7.171604
MB-0893	ER-/HER2-	7.223126		7.223126	7.141805	7.306566
MB-0906	ER-/HER2-	7.093253		7.093253	7.763476	7.134677
MB-2556	ER-/HER2-	7.028613		7.028613	7.359389	6.631249
MB-2753	ER-/HER2-	7.458931		7.458931	7.853239	7.104024
MB-2764	ER-/HER2-	7.084557		7.084557	7.235563	6.698238
MB-2827	ER-/HER2-	7.43508		7.43508	7.100053	7.071496
MB-2834	ER-/HER2-	6.697111		6.697111	7.343252	7.157811
MB-2842	ER-/HER2-	7.974846		7.974846	7.634186	7.187189
MB-2846	ER-/HER2-	7.31221		7.31221	6.687546	7.012857
MB-2849	ER-/HER2-	6.663914		6.663914	7.087432	7.359214
MB-2857	ER-/HER2-	6.429269		6.429269	7.07911	7.09964
MB-2912	ER-/HER2-	7.364163		7.364163	7.2557	6.991073
MB-2917	ER-/HER2-	7.243687		7.243687	6.982149	7.071679

MB-2929	ER-/HER2-	6.959776		6.959776	7.417058	6.530286
MB-2957	ER-/HER2-	7.671357		7.671357	7.47304	7.356204
MB-2963	ER-/HER2-	6.848119		6.848119	6.762397	6.794915
MB-2993	ER-/HER2-	7.325508		7.325508	7.452057	7.427801
MB-3006	ER-/HER2-	7.226807		7.226807	7.319248	6.792229
MB-3014	ER-/HER2-	7.624348		7.624348	7.568561	7.626883
MB-3046	ER-/HER2-	6.910691		6.910691	7.964961	7.301611
MB-3057	ER-/HER2-	7.754311		7.754311	7.394567	6.669404
MB-3058	ER-/HER2-	7.28942		7.28942	8.424466	6.894176
MB-3062	ER-/HER2-	7.207344		7.207344	7.197867	6.935089
MB-3063	ER-/HER2-	7.542936		7.542936	7.314763	7.190469
MB-3067	ER-/HER2-	7.19432		7.19432	7.527287	7.980899
MB-3123	ER-/HER2-	7.699941		7.699941	7.627967	7.527768
MB-3153	ER-/HER2-	7.686533		7.686533	7.565705	7.781698
MB-3165	ER-/HER2-	7.196963		7.196963	6.443113	7.317509
MB-3211	ER-/HER2-	7.383893		7.383893	6.72865	7.114047
MB-3271	ER-/HER2-	6.966566		6.966566	7.379843	6.965915
MB-3277	ER-/HER2-	7.260143		7.260143	7.522119	6.564386
MB-3292	ER-/HER2-	7.279499		7.279499	7.097326	7.458341
MB-3297	ER-/HER2-	6.522626		6.522626	7.50402	7.013192
MB-3363	ER-/HER2-	8.140274		8.140274	7.028674	7.203893
MB-3367	ER-/HER2-	7.361433		7.361433	7.157614	6.969529
MB-3395	ER-/HER2-	7.408412		7.408412	7.097326	7.030661
MB-3396	ER-/HER2-	7.471327		7.471327	6.962495	7.04081
MB-3453	ER-/HER2-	7.137013		7.137013	6.355907	7.615327
MB-3476	ER-/HER2-	6.903765		6.903765	6.674008	7.254902
MB-3479	ER-/HER2-	6.696305		6.696305	6.949631	6.484965
MB-3500	ER-/HER2-	7.078815		7.078815	7.525153	7.198678
MB-3502	ER-/HER2-	6.356015		6.356015	7.21146	7.368707
MB-3567	ER-/HER2-	7.364152		7.364152	7.290234	7.410245
MB-3582	ER-/HER2-	7.299598		7.299598	7.884257	7.426194
MB-3702	ER-/HER2-	7.066343		7.066343	7.116559	6.902475
MB-3706	ER-/HER2-	6.690242		6.690242	7.214746	7.224624
MB-3752	ER-/HER2-	7.420735		7.420735	7.420187	7.333877
MB-4146	ER-/HER2-	7.798859		7.798859	7.264132	7.195297
MB-4408	ER-/HER2-	7.770363		7.770363	7.138406	7.039568
MB-4621	ER-/HER2-	6.398941		6.398941	7.501548	6.955905
MB-4622	ER-/HER2-	6.83918		6.83918	7.43163	7.678382
MB-4667	ER-/HER2-	7.413373		7.413373	7.442261	6.721739
MB-4679	ER-/HER2-	7.759963		7.759963	7.13591	6.775334
MB-4694	ER-/HER2-	8.231941		8.231941	7.726364	7.233991

MB-4696	ER-/HER2-	6.962188	6.962188	7.180852	7.464942
MB-4707	ER-/HER2-	8.60926	8.60926	7.271994	7.460688
MB-4711	ER-/HER2-	7.399026	7.399026	7.354303	7.584761
MB-4714	ER-/HER2-	7.440334	7.440334	7.564699	6.991007
MB-4715	ER-/HER2-	7.388449	7.388449	7.403375	7.577402
MB-4717	ER-/HER2-	7.926798	7.926798	7.419846	7.233764
MB-4732	ER-/HER2-	7.056159	7.056159	8.172225	6.808849
MB-4733	ER-/HER2-	7.641612	7.641612	7.718358	7.669558
MB-4757	ER-/HER2-	8.229299	8.229299	7.594275	7.101531
MB-4758	ER-/HER2-	7.931655	7.931655	7.640861	6.938664
MB-4769	ER-/HER2-	7.249176	7.249176	7.526057	7.017244
MB-4770	ER-/HER2-	8.311829	8.311829	7.30089	7.72144
MB-4782	ER-/HER2-	7.340555	7.340555	7.43581	6.927027
MB-4792	ER-/HER2-	8.041666	8.041666	7.668966	7.185909
MB-4809	ER-/HER2-	7.686675	7.686675	7.642943	7.091972
MB-4828	ER-/HER2-	7.589484	7.589484	7.550625	6.849486
MB-4859	ER-/HER2-	7.826649	7.826649	7.270727	7.654473
MB-4865	ER-/HER2-	7.77931	7.77931	6.956472	7.136683
MB-4876	ER-/HER2-	7.562435	7.562435	7.593289	6.885385
MB-4880	ER-/HER2-	7.504936	7.504936	6.905262	6.694071
MB-4881	ER-/HER2-	8.047263	8.047263	7.125339	7.253215
MB-4888	ER-/HER2-	7.286761	7.286761	7.447353	7.370922
MB-4893	ER-/HER2-	7.494786	7.494786	7.666998	6.931327
MB-4911	ER-/HER2-	7.486838	7.486838	6.937595	7.305533
MB-4938	ER-/HER2-	7.578885	7.578885	7.152123	6.93308
MB-4942	ER-/HER2-	8.184773	8.184773	6.647041	7.940554
MB-4945	ER-/HER2-	8.197518	8.197518	7.347003	6.233423
MB-4974	ER-/HER2-	8.599738	8.599738	7.334181	6.73274
MB-4982	ER-/HER2-	7.423215	7.423215	6.778096	6.940022
MB-4993	ER-/HER2-	7.887798	7.887798	7.031619	7.049302
MB-5008	ER-/HER2-	7.675146	7.675146	7.263227	7.390797
MB-5041	ER-/HER2-	7.70952	7.70952	6.807714	7.168207
MB-5052	ER-/HER2-	7.265666	7.265666	6.699702	7.21865
MB-5057	ER-/HER2-	7.221735	7.221735	6.809308	7.363282
MB-5058	ER-/HER2-	8.019536	8.019536	7.114165	7.216288
MB-5065	ER-/HER2-	8.176248	8.176248	6.83567	7.178386
MB-5070	ER-/HER2-	7.543415	7.543415	6.796344	7.133393
MB-5072	ER-/HER2-	7.272404	7.272404	7.978699	7.156433
MB-5100	ER-/HER2-	7.580345	7.580345	7.79367	7.195269
MB-5115	ER-/HER2-	7.952999	7.952999	7.338656	6.951748
MB-5126	ER-/HER2-	7.234954	7.234954	7.250092	6.952149

MB-5138	ER-/HER2-	7.899758		7.899758	7.288209	7.083114
MB-5145	ER-/HER2-	7.684443		7.684443	7.0571	6.568932
MB-5148	ER-/HER2-	7.901313		7.901313	7.434957	7.146876
MB-5154	ER-/HER2-	7.933353		7.933353	6.956195	7.236407
MB-5157	ER-/HER2-	7.564372		7.564372	6.986046	7.264837
MB-5162	ER-/HER2-	7.796222		7.796222	7.08498	7.358879
MB-5173	ER-/HER2-	8.340484		8.340484	7.225964	7.489524
MB-5188	ER-/HER2-	7.227992		7.227992	6.97303	6.679755
MB-5205	ER-/HER2-	7.450632		7.450632	7.549164	6.435078
MB-5208	ER-/HER2-	7.628832		7.628832	7.050591	6.686564
MB-5209	ER-/HER2-	7.581672		7.581672	7.089697	6.574655
MB-5222	ER-/HER2-	7.420703		7.420703	6.950426	6.770344
MB-5223	ER-/HER2-	7.22995		7.22995	6.682535	6.958482
MB-5225	ER-/HER2-	7.224729		7.224729	7.465471	7.053609
MB-5232	ER-/HER2-	7.192958		7.192958	7.057879	6.843063
MB-5235	ER-/HER2-	7.110213		7.110213	7.387361	6.711829
MB-5236	ER-/HER2-	7.050591		7.050591	6.909404	7.148144
MB-5258	ER-/HER2-	7.419821		7.419821	6.953344	7.193981
MB-5294	ER-/HER2-	7.859818		7.859818	6.428697	7.198213
MB-5295	ER-/HER2-	7.979225		7.979225	6.506324	7.009545
MB-5298	ER-/HER2-	7.661419		7.661419	6.586511	7.633162
MB-5299	ER-/HER2-	7.635039		7.635039	6.361094	7.482432
MB-5311	ER-/HER2-	7.829086		7.829086	7.75306	6.86182
MB-5323	ER-/HER2-	7.681293		7.681293	7.361421	6.951542
MB-5335	ER-/HER2-	7.357497		7.357497	7.20662	6.74306
MB-5348	ER-/HER2-	7.635715		7.635715	6.570809	7.021679
MB-5350	ER-/HER2-	7.434791		7.434791	6.922338	7.067661
MB-5378	ER-/HER2-	7.664102		7.664102	7.377302	6.82517
MB-5390	ER-/HER2-	7.768901		7.768901	6.971954	7.20002
MB-5392	ER-/HER2-	7.16774		7.16774	7.497559	6.63404
MB-5408	ER-/HER2-	7.465074		7.465074	7.119797	6.84008
MB-5421	ER-/HER2-	8.042436		8.042436	7.772566	6.974823
MB-5427	ER-/HER2-	9.881328		9.881328	7.62933	6.841797
MB-5431	ER-/HER2-	7.724859		7.724859	6.491419	6.431235
MB-5440	ER-/HER2-	7.656987		7.656987	7.280171	6.470194
MB-5442	ER-/HER2-	6.872579		6.872579	8.224743	6.510734
MB-5446	ER-/HER2-	7.907051		7.907051	7.597535	6.774516
MB-5450	ER-/HER2-	7.092793		7.092793	8.205299	7.219968
MB-5453	ER-/HER2-	7.204286		7.204286	7.948874	7.081133
MB-5465	ER-/HER2-	7.633726		7.633726	7.130907	6.82803
MB-5468	ER-/HER2-	7.980928		7.980928	7.173129	6.937218

MB-5482	ER-/HER2-	7.898693		7.898693	6.684667	6.884624
MB-5511	ER-/HER2-	7.027246		7.027246	7.209049	6.956472
MB-5526	ER-/HER2-	7.750047		7.750047	6.809002	7.039568
MB-5527	ER-/HER2-	8.07878		8.07878	7.233337	6.44405
MB-5529	ER-/HER2-	8.079843		8.079843	7.298028	6.875285
MB-5531	ER-/HER2-	7.935344		7.935344	7.145715	6.862673
MB-5534	ER-/HER2-	7.937225		7.937225	7.346185	6.648073
MB-5547	ER-/HER2-	7.759453		7.759453	8.048848	6.536718
MB-5548	ER-/HER2-	7.241322		7.241322	9.0864	6.681799
MB-5549	ER-/HER2-	8.063337		8.063337	7.300028	6.774859
MB-5551	ER-/HER2-	7.949734		7.949734	7.615934	6.63716
MB-5559	ER-/HER2-	7.57992		7.57992	7.828624	6.70398
MB-5560	ER-/HER2-	7.117366		7.117366	7.46186	7.126752
MB-5565	ER-/HER2-	7.126105		7.126105	7.21433	7.126586
MB-5566	ER-/HER2-	7.295257		7.295257	7.258264	6.479476
MB-5572	ER-/HER2-	7.661419		7.661419	7.416144	7.469726
MB-5588	ER-/HER2-	7.115624		7.115624	6.874219	6.583376
MB-5602	ER-/HER2-	8.512691		8.512691	6.923363	6.503815
MB-5616	ER-/HER2-	7.822312		7.822312	7.592273	7.124724
MB-5633	ER-/HER2-	7.681096		7.681096	6.954943	7.535157
MB-5634	ER-/HER2-	7.170588		7.170588	6.751203	7.022926
MB-5651	ER-/HER2-	7.605814		7.605814	6.970851	6.97954
MB-5652	ER-/HER2-	7.411268		7.411268	7.230103	7.07641
MB-5655	ER-/HER2-	8.364888		8.364888	8.125551	7.011975
MB-6036	ER-/HER2-	8.308784		8.308784	7.112686	7.013018
MB-6052	ER-/HER2-	7.2324		7.2324	7.66028	7.595259
MB-6055	ER-/HER2-	7.336965		7.336965	7.548845	7.483461
MB-6058	ER-/HER2-	7.280037		7.280037	6.88692	7.019562
MB-6062	ER-/HER2-	7.485945		7.485945	6.694148	6.817704
MB-6085	ER-/HER2-	7.633244		7.633244	7.270654	6.538706
MB-6122	ER-/HER2-	8.085648		8.085648	7.050106	7.130927
MB-6143	ER-/HER2-	8.894197		8.894197	7.710134	7.131324
MB-6144	ER-/HER2-	7.706659		7.706659	7.117522	6.82287
MB-6152	ER-/HER2-	7.84583		7.84583	7.740439	7.132986
MB-6169	ER-/HER2-	7.924265		7.924265	7.71802	6.708765
MB-6178	ER-/HER2-	7.610723		7.610723	7.248977	6.789392
MB-6187	ER-/HER2-	7.392781		7.392781	7.54879	7.89844
MB-6188	ER-/HER2-	7.573773		7.573773	7.394747	7.654236
MB-6223	ER-/HER2-	7.694337		7.694337	8.225117	7.069323
MB-6224	ER-/HER2-	7.311327		7.311327	7.503046	7.741473
MB-6228	ER-/HER2-	7.825562		7.825562	6.639013	7.511412

MB-6237	ER-/HER2-	7.714534		7.714534	6.860048	7.460029
MB-6242	ER-/HER2-	6.864567		6.864567	7.43082	7.279183
MB-6245	ER-/HER2-	8.462743		8.462743	6.771219	7.450437
MB-6248	ER-/HER2-	7.564746		7.564746	7.166599	6.982545
MB-6305	ER-/HER2-	7.200443		7.200443	7.708616	7.222928
MB-6318	ER-/HER2-	7.31221		7.31221	6.978793	7.229006
MB-6336	ER-/HER2-	7.765013		7.765013	8.293522	7.175392
MB-7007	ER-/HER2-	7.442613		7.442613	7.686007	6.819542
MB-7008	ER-/HER2-	7.364242		7.364242	7.413478	6.937497
MB-7009	ER-/HER2-	8.296164		8.296164	7.60357	7.150305
MB-7012	ER-/HER2-	7.861422		7.861422	8.173646	6.864793
MB-7017	ER-/HER2-	7.230715		7.230715	7.093191	6.805233
MB-7023	ER-/HER2-	7.311774		7.311774	7.427866	7.19432
MB-7025	ER-/HER2-	7.331663		7.331663	8.205326	7.022218
MB-7030	ER-/HER2-	7.556796		7.556796	7.938446	7.439348
MB-7036	ER-/HER2-	6.962998		6.962998	7.736524	6.986224
MB-7038	ER-/HER2-	7.716491		7.716491	7.892319	7.535487
MB-7039	ER-/HER2-	7.571901		7.571901	7.567458	7.955341
MB-7045	ER-/HER2-	7.201295		7.201295	7.960245	7.450496
MB-7049	ER-/HER2-	8.148408		8.148408	7.685734	7.779824
MB-7052	ER-/HER2-	6.957384		6.957384	7.98148	6.933289
MB-7054	ER-/HER2-	7.905575		7.905575	7.131622	7.541629
MB-7078	ER-/HER2-	7.236649		7.236649	7.848058	7.713472
MB-7079	ER-/HER2-	7.161547		7.161547	7.241511	7.562896
MB-7081	ER-/HER2-	7.010774		7.010774	7.914089	7.50402
MB-7084	ER-/HER2-	6.810937		6.810937	7.177084	6.949774
MB-7089	ER-/HER2-	7.449831		7.449831	7.115859	7.665343
MB-7090	ER-/HER2-	7.196139		7.196139	7.265706	7.658739
MB-7104	ER-/HER2-	7.745419		7.745419	7.951849	7.278703
MB-7114	ER-/HER2-	6.513975		6.513975	8.165663	7.336925
MB-7119	ER-/HER2-	7.964042		7.964042	8.208015	7.79672
MB-7121	ER-/HER2-	7.0112		7.0112	7.445196	7.862439
MB-7145	ER-/HER2-	6.816925		6.816925	7.652446	8.019562
MB-7151	ER-/HER2-	7.062903		7.062903	7.599671	7.200875
MB-7154	ER-/HER2-	8.61802		8.61802	6.465022	7.061671
MB-7155	ER-/HER2-	7.122697		7.122697	7.500173	7.710902
MB-7158	ER-/HER2-	6.983997		6.983997	8.47531	7.790056
MB-7165	ER-/HER2-	6.402056		6.402056	8.003509	7.759356
MB-7201	ER-/HER2-	6.653797		6.653797	7.704173	8.249279
MB-7205	ER-/HER2-	7.514677		7.514677	7.699551	7.762341
MB-7207	ER-/HER2-	7.028674		7.028674	8.109152	7.373334

MB-7208	ER-/HER2-	6.753395	6.753395	7.726743	6.938568
MB-7252	ER-/HER2-	6.704625	6.704625	7.850091	6.495719
MB-7269	ER-/HER2-	7.347707	7.347707	7.799833	6.876517
MB-7270	ER-/HER2-	7.467237	7.467237	6.672085	7.537104
MB-0002	ER+/HER2- Hi	7.716212		7.649722	7.707053
MB-0008	ER+/HER2- Hi	7.172573		6.908406	7.453646
MB-0010	ER+/HER2- Hi	7.080922		8.00559	7.699696
MB-0028	ER+/HER2- Hi	6.906297		7.428168	7.315788
MB-0035	ER+/HER2- Hi	7.799011		7.610225	7.156118
MB-0060	ER+/HER2- Hi	7.925118		7.811517	7.193622
MB-0066	ER+/HER2- Hi	7.538038		8.073727	7.051848
MB-0102	ER+/HER2- Hi	7.091365		7.851715	7.725257
MB-0109	ER+/HER2- Hi	7.912455		8.743363	7.670217
MB-0114	ER+/HER2- Hi	7.212006		7.686533	6.795259
MB-0116	ER+/HER2- Hi	7.048093		7.366726	7.454117
MB-0119	ER+/HER2- Hi	7.225232		7.532396	7.588488
MB-0120	ER+/HER2- Hi	6.512082		7.491279	7.412514
MB-0123	ER+/HER2- Hi	6.835758		7.357357	7.178033
MB-0131	ER+/HER2- Hi	7.109417		7.907051	7.555681
MB-0134	ER+/HER2- Hi	7.434772		7.905952	7.060161
MB-0135	ER+/HER2- Hi	7.541629		7.216318	7.530514
MB-0140	ER+/HER2- Hi	6.327295		7.970726	7.265706
MB-0143	ER+/HER2- Hi	7.965306		7.943161	7.108693
MB-0146	ER+/HER2- Hi	7.89807		7.864795	7.897158
MB-0147	ER+/HER2- Hi	7.538969		7.567023	7.823506
MB-0151	ER+/HER2- Hi	7.778228		7.667007	7.133393
MB-0167	ER+/HER2- Hi	7.554356		7.885904	7.465533
MB-0173	ER+/HER2- Hi	7.711101		6.704392	7.310023
MB-0176	ER+/HER2- Hi	7.36352		6.623339	7.691346
MB-0193	ER+/HER2- Hi	7.566647		8.058974	8.546868
MB-0195	ER+/HER2- Hi	7.278049		7.274533	7.781876
MB-0197	ER+/HER2- Hi	6.93273		6.893485	7.691164
MB-0198	ER+/HER2- Hi	7.269788		7.416567	7.31711
MB-0202	ER+/HER2- Hi	7.098351		7.561319	7.474774
MB-0203	ER+/HER2- Hi	7.079512		7.241367	7.495652
MB-0215	ER+/HER2- Hi	7.145187		7.114461	7.541287
MB-0218	ER+/HER2- Hi	7.601986		8.001133	8.129807
MB-0221	ER+/HER2- Hi	7.068848		7.71993	7.588391
MB-0234	ER+/HER2- Hi	7.490105		7.417167	6.945346
MB-0257	ER+/HER2- Hi	6.812535		7.532913	8.323382
MB-0258	ER+/HER2- Hi	7.364654		7.951399	7.214767

MB-0261	ER+/HER2- Hi	6.686828	7.392567	7.785486
MB-0270	ER+/HER2- Hi	6.705479	6.809316	7.658852
MB-0272	ER+/HER2- Hi	7.044099	8.398016	7.102795
MB-0287	ER+/HER2- Hi	7.109542	8.279438	7.350541
MB-0306	ER+/HER2- Hi	7.162324	7.448852	7.652492
MB-0311	ER+/HER2- Hi	7.722806	7.811201	7.83128
MB-0312	ER+/HER2- Hi	7.045967	7.229218	7.736386
MB-0313	ER+/HER2- Hi	6.943495	7.409416	7.180852
MB-0319	ER+/HER2- Hi	6.721924	7.576929	7.130138
MB-0321	ER+/HER2- Hi	7.134587	7.65767	7.878063
MB-0324	ER+/HER2- Hi	7.379266	7.980881	7.228855
MB-0325	ER+/HER2- Hi	7.761783	7.033871	7.359048
MB-0328	ER+/HER2- Hi	7.376044	7.468159	7.519334
MB-0336	ER+/HER2- Hi	7.356844	7.096732	7.87292
MB-0341	ER+/HER2- Hi	7.295405	7.737039	8.34753
MB-0349	ER+/HER2- Hi	6.739426	6.951727	7.662924
MB-0356	ER+/HER2- Hi	7.316642	7.368859	8.045639
MB-0358	ER+/HER2- Hi	7.084345	8.094446	7.575199
MB-0360	ER+/HER2- Hi	6.850091	7.413931	7.295151
MB-0362	ER+/HER2- Hi	7.257938	7.892851	7.506069
MB-0363	ER+/HER2- Hi	7.005793	8.259751	7.891384
MB-0366	ER+/HER2- Hi	7.413018	7.380081	7.716981
MB-0368	ER+/HER2- Hi	6.813402	7.880416	7.309463
MB-0370	ER+/HER2- Hi	7.406483	7.445198	7.311293
MB-0374	ER+/HER2- Hi	7.923486	7.572372	7.676548
MB-0380	ER+/HER2- Hi	6.866491	7.799698	7.790479
MB-0383	ER+/HER2- Hi	7.867488	7.158461	7.38483
MB-0384	ER+/HER2- Hi	7.455339	7.497559	6.687311
MB-0385	ER+/HER2- Hi	8.294131	7.229006	7.323473
MB-0392	ER+/HER2- Hi	7.613565	7.649811	7.287888
MB-0393	ER+/HER2- Hi	7.726851	7.471084	7.432938
MB-0394	ER+/HER2- Hi	7.141805	8.439731	7.309569
MB-0398	ER+/HER2- Hi	7.763476	7.542604	7.56951
MB-0404	ER+/HER2- Hi	7.359389	7.144447	6.99444
MB-0406	ER+/HER2- Hi	7.853239	7.983816	7.470442
MB-0412	ER+/HER2- Hi	7.235563	7.41562	7.399305
MB-0413	ER+/HER2- Hi	7.100053	7.750848	7.176658
MB-0417	ER+/HER2- Hi	7.343252	7.895051	6.883131
MB-0418	ER+/HER2- Hi	7.634186	7.582185	6.935372
MB-0427	ER+/HER2- Hi	6.687546	7.885393	7.445428
MB-0428	ER+/HER2- Hi	7.087432	6.755583	7.604758

MB-0429	ER+/HER2- Hi	7.07911		8.691893	7.023354
MB-0437	ER+/HER2- Hi	7.2557		8.091253	7.807029
MB-0439	ER+/HER2- Hi	6.982149		7.13225	7.212076
MB-0440	ER+/HER2- Hi	7.417058		8.081611	7.167328
MB-0445	ER+/HER2- Hi	7.47304		6.756908	7.379103
MB-0452	ER+/HER2- Hi	6.762397		7.946164	7.098212
MB-0453	ER+/HER2- Hi	7.452057		7.138381	7.632299
MB-0455	ER+/HER2- Hi	7.319248		7.72144	7.483194
MB-0459	ER+/HER2- Hi	7.568561		7.169438	6.876517
MB-0466	ER+/HER2- Hi	7.964961		7.437184	7.336885
MB-0469	ER+/HER2- Hi	7.394567		7.150276	7.883133
MB-0471	ER+/HER2- Hi	8.424466		7.334231	7.193981
MB-0472	ER+/HER2- Hi	7.197867		7.841148	7.766011
MB-0474	ER+/HER2- Hi	7.314763		7.578358	7.858793
MB-0475	ER+/HER2- Hi	7.527287		8.361707	7.643842
MB-0483	ER+/HER2- Hi	7.627967		7.881012	7.623158
MB-0484	ER+/HER2- Hi	7.565705		6.934152	7.110608
MB-0485	ER+/HER2- Hi	6.443113		7.929393	6.807725
MB-0491	ER+/HER2- Hi	6.72865		7.610225	7.855662
MB-0492	ER+/HER2- Hi	7.379843		6.832479	7.499326
MB-0506	ER+/HER2- Hi	7.522119		7.541605	7.658963
MB-0508	ER+/HER2- Hi	7.097326		7.627962	7.165676
MB-0509	ER+/HER2- Hi	7.50402		7.393211	7.680534
MB-0510	ER+/HER2- Hi	7.028674		7.325068	7.259564
MB-0514	ER+/HER2- Hi	7.157614		7.936133	7.938701
MB-0526	ER+/HER2- Hi	7.097326		7.075557	7.546488
MB-0529	ER+/HER2- Hi	6.962495		8.20497	7.275358
MB-0532	ER+/HER2- Hi	6.355907		7.375189	7.392249
MB-0536	ER+/HER2- Hi	6.674008		7.66408	7.261445
MB-0538	ER+/HER2- Hi	6.949631		7.333632	7.95357
MB-0541	ER+/HER2- Hi	7.525153		7.460939	7.670111
MB-0542	ER+/HER2- Hi	7.21146		7.573773	7.985337
MB-0545	ER+/HER2- Hi	7.290234		6.923491	7.873388
MB-0550	ER+/HER2- Hi	7.884257		7.661691	7.217941
MB-0559	ER+/HER2- Hi	7.116559		7.584073	7.359574
MB-0569	ER+/HER2- Hi	7.214746		7.21611	7.793692
MB-0570	ER+/HER2- Hi	7.420187		7.422775	7.382025
MB-0571	ER+/HER2- Hi	7.264132		7.822106	7.279233
MB-0574	ER+/HER2- Hi	7.138406		7.542165	7.337105
MB-0576	ER+/HER2- Hi	7.501548		8.374817	7.024764
MB-0577	ER+/HER2- Hi	7.43163		7.317509	7.288315

MB-0580	ER+/HER2- Hi	7.442261	7.200443	6.719613
MB-0584	ER+/HER2- Hi	7.13591	8.046706	6.807428
MB-0585	ER+/HER2- Hi	7.726364	7.151637	7.34097
MB-0587	ER+/HER2- Hi	7.180852	6.990406	7.389015
MB-0589	ER+/HER2- Hi	7.271994	7.496627	7.89652
MB-0590	ER+/HER2- Hi	7.354303	7.448012	7.692906
MB-0591	ER+/HER2- Hi	7.564699	7.575377	7.759807
MB-0594	ER+/HER2- Hi	7.403375	7.961646	7.072999
MB-0598	ER+/HER2- Hi	7.419846	7.306923	7.237495
MB-0600	ER+/HER2- Hi	8.172225	8.123825	6.974383
MB-0601	ER+/HER2- Hi	7.718358	7.390874	7.697077
MB-0606	ER+/HER2- Hi	7.594275	8.351828	7.65103
MB-0607	ER+/HER2- Hi	7.640861	7.645975	7.13892
MB-0609	ER+/HER2- Hi	7.526057	8.072976	7.235324
MB-0611	ER+/HER2- Hi	7.30089	7.951307	7.11717
MB-0614	ER+/HER2- Hi	7.43581	7.612621	7.484739
MB-0616	ER+/HER2- Hi	7.668966	7.679816	7.499048
MB-0619	ER+/HER2- Hi	7.642943	7.56965	7.420636
MB-0628	ER+/HER2- Hi	7.550625	7.567129	6.87989
MB-0630	ER+/HER2- Hi	7.270727	7.95582	6.660783
MB-0636	ER+/HER2- Hi	6.956472	7.32559	7.004781
MB-0637	ER+/HER2- Hi	7.593289	7.834407	7.439674
MB-0650	ER+/HER2- Hi	6.905262	8.084787	7.163673
MB-0653	ER+/HER2- Hi	7.125339	7.567993	7.415708
MB-0660	ER+/HER2- Hi	7.447353	7.383855	6.97161
MB-0666	ER+/HER2- Hi	7.666998	7.460833	7.557849
MB-0877	ER+/HER2- Hi	6.937595	7.895471	7.021639
MB-0882	ER+/HER2- Hi	7.152123	7.945222	6.993505
MB-0884	ER+/HER2- Hi	6.647041	7.989111	6.527903
MB-0899	ER+/HER2- Hi	7.347003	7.097787	7.238627
MB-2613	ER+/HER2- Hi	7.334181	7.886488	6.870745
MB-2617	ER+/HER2- Hi	6.778096	7.547266	7.123107
MB-2634	ER+/HER2- Hi	7.031619	7.382086	7.670217
MB-2642	ER+/HER2- Hi	7.263227	7.335111	7.586581
MB-2686	ER+/HER2- Hi	6.807714	7.541627	7.118185
MB-2705	ER+/HER2- Hi	6.699702	7.66971	7.474975
MB-2708	ER+/HER2- Hi	6.809308	8.065573	7.547441
MB-2728	ER+/HER2- Hi	7.114165	7.647303	7.690237
MB-2730	ER+/HER2- Hi	6.83567	7.245394	8.148776
MB-2745	ER+/HER2- Hi	6.796344	7.095884	7.862035
MB-2763	ER+/HER2- Hi	7.978699	7.838277	7.674681

MB-2767	ER+/HER2- Hi	7.79367	7.43163	7.657481
MB-2769	ER+/HER2- Hi	7.338656	7.235474	7.471674
MB-2772	ER+/HER2- Hi	7.250092	7.744001	7.253933
MB-2774	ER+/HER2- Hi	7.288209	7.316661	7.521681
MB-2778	ER+/HER2- Hi	7.0571	7.753341	6.925131
MB-2779	ER+/HER2- Hi	7.434957	7.483639	7.186584
MB-2781	ER+/HER2- Hi	6.956195	7.275293	7.371988
MB-2790	ER+/HER2- Hi	6.986046	7.709041	7.492879
MB-2792	ER+/HER2- Hi	7.08498	8.301518	7.553813
MB-2793	ER+/HER2- Hi	7.225964	7.283934	7.466755
MB-2796	ER+/HER2- Hi	6.97303	7.080921	6.922544
MB-2797	ER+/HER2- Hi	7.549164	7.26613	7.540608
MB-2801	ER+/HER2- Hi	7.050591	7.560571	6.706813
MB-2803	ER+/HER2- Hi	7.089697	8.052868	7.71295
MB-2814	ER+/HER2- Hi	6.950426	7.214994	7.685232
MB-2838	ER+/HER2- Hi	6.682535	6.860766	7.374513
MB-2853	ER+/HER2- Hi	7.465471	7.566647	7.945656
MB-2858	ER+/HER2- Hi	7.057879	7.578009	7.882657
MB-2922	ER+/HER2- Hi	7.387361	7.363335	7.208281
MB-2927	ER+/HER2- Hi	6.909404	8.199083	7.258642
MB-2932	ER+/HER2- Hi	6.953344	7.720476	7.345054
MB-2951	ER+/HER2- Hi	6.428697	7.633726	7.151199
MB-2953	ER+/HER2- Hi	6.506324	7.52966	7.699202
MB-2954	ER+/HER2- Hi	6.586511	7.476342	7.827173
MB-2966	ER+/HER2- Hi	6.361094	8.069828	7.519171
MB-2999	ER+/HER2- Hi	7.75306	7.211157	7.189823
MB-3002	ER+/HER2- Hi	7.361421	7.587537	7.431378
MB-3007	ER+/HER2- Hi	7.20662	7.507252	7.640432
MB-3016	ER+/HER2- Hi	6.570809	6.999224	7.811454
MB-3021	ER+/HER2- Hi	6.922338	7.3696	7.259988
MB-3026	ER+/HER2- Hi	7.377302	7.57992	7.600473
MB-3028	ER+/HER2- Hi	6.971954	7.941368	7.893489
MB-3031	ER+/HER2- Hi	7.497559	7.208212	7.336601
MB-3050	ER+/HER2- Hi	7.119797	7.577534	7.285545
MB-3060	ER+/HER2- Hi	7.772566	7.515154	7.755574
MB-3083	ER+/HER2- Hi	7.62933	8.200882	7.589506
MB-3092	ER+/HER2- Hi	6.491419	7.56102	7.682944
MB-3102	ER+/HER2- Hi	7.280171	8.07709	7.411268
MB-3104	ER+/HER2- Hi	8.224743	8.290472	7.210128
MB-3167	ER+/HER2- Hi	7.597535	7.302215	7.101087
MB-3235	ER+/HER2- Hi	8.205299	7.469995	7.546115

MB-3253	ER+/HER2- Hi	7.948874	7.288743	7.471735
MB-3266	ER+/HER2- Hi	7.130907	7.604937	7.50402
MB-3275	ER+/HER2- Hi	7.173129	6.746778	7.831921
MB-3300	ER+/HER2- Hi	6.684667	7.280482	7.661314
MB-3301	ER+/HER2- Hi	7.209049	7.192729	7.901313
MB-3303	ER+/HER2- Hi	6.809002	7.355773	7.521681
MB-3341	ER+/HER2- Hi	7.233337	8.81889	7.421092
MB-3350	ER+/HER2- Hi	7.298028	7.566169	7.040784
MB-3357	ER+/HER2- Hi	7.145715	7.791616	7.775632
MB-3371	ER+/HER2- Hi	7.346185	7.175825	7.63855
MB-3388	ER+/HER2- Hi	8.048848	7.895995	6.994825
MB-3389	ER+/HER2- Hi	9.0864	6.799567	7.641001
MB-3402	ER+/HER2- Hi	7.300028	7.605424	7.02008
MB-3417	ER+/HER2- Hi	7.615934	8.088452	7.510925
MB-3430	ER+/HER2- Hi	7.828624	7.265706	7.293535
MB-3437	ER+/HER2- Hi	7.46186	7.151524	7.343252
MB-3459	ER+/HER2- Hi	7.21433	7.237945	7.489604
MB-3466	ER+/HER2- Hi	7.258264	7.351246	7.926861
MB-3487	ER+/HER2- Hi	7.416144	7.990767	7.314065
MB-3490	ER+/HER2- Hi	6.874219	8.537321	7.403533
MB-3492	ER+/HER2- Hi	6.923363	7.13426	7.052626
MB-3506	ER+/HER2- Hi	7.592273	7.660887	7.328591
MB-3525	ER+/HER2- Hi	6.954943	7.309149	7.765512
MB-3530	ER+/HER2- Hi	6.751203	7.618367	6.596639
MB-3536	ER+/HER2- Hi	6.970851	7.874912	7.462227
MB-3548	ER+/HER2- Hi	7.230103	7.872295	7.544569
MB-3556	ER+/HER2- Hi	8.125551	6.913334	7.596713
MB-3576	ER+/HER2- Hi	7.112686	6.719444	7.340555
MB-3600	ER+/HER2- Hi	7.66028	7.001605	7.391482
MB-3614	ER+/HER2- Hi	7.548845	7.846866	7.973299
MB-3754	ER+/HER2- Hi	6.88692	6.592496	7.747434
MB-3797	ER+/HER2- Hi	6.694148	7.320624	7.395477
MB-3824	ER+/HER2- Hi	7.270654	7.238376	7.398109
MB-3838	ER+/HER2- Hi	7.050106	6.855755	7.521681
MB-3840	ER+/HER2- Hi	7.710134	6.705706	7.836462
MB-3842	ER+/HER2- Hi	7.117522	7.52264	8.264613
MB-3852	ER+/HER2- Hi	7.740439	7.814172	7.515154
MB-4001	ER+/HER2- Hi	7.71802	7.57992	7.601555
MB-4003	ER+/HER2- Hi	7.248977	8.091861	7.657481
MB-4004	ER+/HER2- Hi	7.54879	6.758905	7.820392
MB-4011	ER+/HER2- Hi	7.394747	7.359764	7.428894

MB-4017	ER+/HER2- Hi	8.225117	7.231557	7.496171
MB-4046	ER+/HER2- Hi	7.503046	7.128816	7.456273
MB-4139	ER+/HER2- Hi	6.639013	6.983205	7.72292
MB-4148	ER+/HER2- Hi	6.860048	7.485945	7.539906
MB-4154	ER+/HER2- Hi	7.43082	7.389261	7.48139
MB-4171	ER+/HER2- Hi	6.771219	6.800736	7.84425
MB-4233	ER+/HER2- Hi	7.166599	7.189308	7.988028
MB-4236	ER+/HER2- Hi	7.708616	7.551683	7.745419
MB-4264	ER+/HER2- Hi	6.978793	7.083769	7.593792
MB-4266	ER+/HER2- Hi	8.293522	7.493368	7.054675
MB-4274	ER+/HER2- Hi	7.686007	6.766269	7.589946
MB-4281	ER+/HER2- Hi	7.413478	7.31221	7.260007
MB-4289	ER+/HER2- Hi	7.60357	7.033082	7.524017
MB-4310	ER+/HER2- Hi	8.173646	7.423823	7.286584
MB-4323	ER+/HER2- Hi	7.093191	6.721995	7.559636
MB-4421	ER+/HER2- Hi	7.427866	7.066144	7.158636
MB-4618	ER+/HER2- Hi	8.205326	6.976418	6.943497
MB-4623	ER+/HER2- Hi	7.938446	7.696757	6.859587
MB-4627	ER+/HER2- Hi	7.736524	7.712541	7.105653
MB-4630	ER+/HER2- Hi	7.892319	7.273884	6.780055
MB-4634	ER+/HER2- Hi	7.567458	7.336965	7.167025
MB-4639	ER+/HER2- Hi	7.960245	7.373147	6.963369
MB-4640	ER+/HER2- Hi	7.685734	7.034707	7.05795
MB-4642	ER+/HER2- Hi	7.98148	6.964531	7.457668
MB-4648	ER+/HER2- Hi	7.131622	7.034707	7.014853
MB-4649	ER+/HER2- Hi	7.848058	8.006793	6.708241
MB-4651	ER+/HER2- Hi	7.241511	7.220911	6.6429
MB-4654	ER+/HER2- Hi	7.914089	7.555888	7.113933
MB-4655	ER+/HER2- Hi	7.177084	7.352162	7.215417
MB-4666	ER+/HER2- Hi	7.115859	7.136789	7.246168
MB-4669	ER+/HER2- Hi	7.265706	6.670218	7.421092
MB-4671	ER+/HER2- Hi	7.951849	7.361976	7.480019
MB-4672	ER+/HER2- Hi	8.165663	7.245304	6.950554
MB-4673	ER+/HER2- Hi	8.208015	7.054675	6.676248
MB-4682	ER+/HER2- Hi	7.445196	7.668256	7.305699
MB-4685	ER+/HER2- Hi	7.652446	8.551398	7.320192
MB-4687	ER+/HER2- Hi	7.599671	6.611195	6.936829
MB-4702	ER+/HER2- Hi	6.465022	7.144407	6.996418
MB-4712	ER+/HER2- Hi	7.500173	7.186769	7.422457
MB-4723	ER+/HER2- Hi	8.47531	7.013241	7.169519
MB-4737	ER+/HER2- Hi	8.003509	7.140995	7.393211

MB-4746	ER+/HER2- Hi	7.704173	7.490105	7.068213
MB-4749	ER+/HER2- Hi	7.699551	8.137396	6.584549
MB-4750	ER+/HER2- Hi	8.109152	6.951751	6.543367
MB-4767	ER+/HER2- Hi	7.726743	7.387914	7.614985
MB-4787	ER+/HER2- Hi	7.850091	7.210735	6.812792
MB-4790	ER+/HER2- Hi	7.799833	7.031487	7.231134
MB-4791	ER+/HER2- Hi	6.672085	7.463609	7.040369
MB-4794	ER+/HER2- Hi	7.649722	7.274318	6.758905
MB-4797	ER+/HER2- Hi	6.908406	7.136789	7.127166
MB-4800	ER+/HER2- Hi	8.00559	7.692411	7.286584
MB-4801	ER+/HER2- Hi	7.428168	7.276925	6.856521
MB-4802	ER+/HER2- Hi	7.610225	7.88912	6.923887
MB-4805	ER+/HER2- Hi	7.811517	7.517946	7.041582
MB-4829	ER+/HER2- Hi	8.073727	7.646353	6.932112
MB-4834	ER+/HER2- Hi	7.851715	6.605952	7.380274
MB-4836	ER+/HER2- Hi	8.743363	7.762986	7.081334
MB-4839	ER+/HER2- Hi	7.686533	7.731395	6.88075
MB-4849	ER+/HER2- Hi	7.366726	7.140592	6.883094
MB-4851	ER+/HER2- Hi	7.532396	7.564746	7.077166
MB-4862	ER+/HER2- Hi	7.491279	6.794313	7.074328
MB-4894	ER+/HER2- Hi	7.357357	7.541311	6.86155
MB-4900	ER+/HER2- Hi	7.907051	7.062903	6.912935
MB-4906	ER+/HER2- Hi	7.905952	7.84425	7.338318
MB-4912	ER+/HER2- Hi	7.216318	7.303078	6.725213
MB-4933	ER+/HER2- Hi	7.970726	6.872299	7.031487
MB-4934	ER+/HER2- Hi	7.943161	7.421557	7.050548
MB-4937	ER+/HER2- Hi	7.864795	7.475436	7.107751
MB-4944	ER+/HER2- Hi	7.567023	6.676953	6.85
MB-4956	ER+/HER2- Hi	7.667007	7.483194	7.030252
MB-4965	ER+/HER2- Hi	7.885904	6.587989	7.018855
MB-4969	ER+/HER2- Hi	6.704392		6.474864
MB-4970	ER+/HER2- Hi	6.623339		7.293535
MB-4986	ER+/HER2- Hi	8.058974		6.775941
MB-4991	ER+/HER2- Hi	7.274533		6.407379
MB-4994	ER+/HER2- Hi	6.893485		7.183373
MB-4998	ER+/HER2- Hi	7.416567		6.940732
MB-5001	ER+/HER2- Hi	7.561319		7.245723
MB-5014	ER+/HER2- Hi	7.241367		7.318836
MB-5017	ER+/HER2- Hi	7.114461		7.437116
MB-5018	ER+/HER2- Hi	8.001133		6.470648
MB-5033	ER+/HER2- Hi	7.71993		6.755221

MB-5035	ER+/HER2- Hi	7.417167	7.374062
MB-5039	ER+/HER2- Hi	7.532913	6.930172
MB-5040	ER+/HER2- Hi	7.951399	7.226912
MB-5044	ER+/HER2- Hi	7.392567	7.143147
MB-5045	ER+/HER2- Hi	6.809316	6.765162
MB-5048	ER+/HER2- Hi	8.398016	
MB-5049	ER+/HER2- Hi	8.279438	
MB-5060	ER+/HER2- Hi	7.448852	
MB-5061	ER+/HER2- Hi	7.811201	
MB-5074	ER+/HER2- Hi	7.229218	
MB-5081	ER+/HER2- Hi	7.409416	
MB-5093	ER+/HER2- Hi	7.576929	
MB-5097	ER+/HER2- Hi	7.65767	
MB-5098	ER+/HER2- Hi	7.980881	
MB-5101	ER+/HER2- Hi	7.033871	
MB-5105	ER+/HER2- Hi	7.468159	
MB-5107	ER+/HER2- Hi	7.096732	
MB-5116	ER+/HER2- Hi	7.737039	
MB-5117	ER+/HER2- Hi	6.951727	
MB-5121	ER+/HER2- Hi	7.368859	
MB-5124	ER+/HER2- Hi	8.094446	
MB-5127	ER+/HER2- Hi	7.413931	
MB-5130	ER+/HER2- Hi	7.892851	
MB-5139	ER+/HER2- Hi	8.259751	
MB-5144	ER+/HER2- Hi	7.380081	
MB-5152	ER+/HER2- Hi	7.880416	
MB-5160	ER+/HER2- Hi	7.445198	
MB-5167	ER+/HER2- Hi	7.572372	
MB-5169	ER+/HER2- Hi	7.799698	
MB-5176	ER+/HER2- Hi	7.158461	
MB-5177	ER+/HER2- Hi	7.497559	
MB-5186	ER+/HER2- Hi	7.229006	
MB-5193	ER+/HER2- Hi	7.649811	
MB-5196	ER+/HER2- Hi	7.471084	
MB-5197	ER+/HER2- Hi	8.439731	
MB-5200	ER+/HER2- Hi	7.542604	
MB-5211	ER+/HER2- Hi	7.144447	
MB-5215	ER+/HER2- Hi	7.983816	
MB-5218	ER+/HER2- Hi	7.41562	
MB-5226	ER+/HER2- Hi	7.750848	
MB-5227	ER+/HER2- Hi	7.895051	

MB-5228	ER+/HER2- Hi	7.582185
MB-5233	ER+/HER2- Hi	7.885393
MB-5239	ER+/HER2- Hi	6.755583
MB-5243	ER+/HER2- Hi	8.691893
MB-5244	ER+/HER2- Hi	8.091253
MB-5256	ER+/HER2- Hi	7.13225
MB-5260	ER+/HER2- Hi	8.081611
MB-5261	ER+/HER2- Hi	6.756908
MB-5266	ER+/HER2- Hi	7.946164
MB-5270	ER+/HER2- Hi	7.138381
MB-5271	ER+/HER2- Hi	7.72144
MB-5272	ER+/HER2- Hi	7.169438
MB-5273	ER+/HER2- Hi	7.437184
MB-5275	ER+/HER2- Hi	7.150276
MB-5279	ER+/HER2- Hi	7.334231
MB-5280	ER+/HER2- Hi	7.841148
MB-5288	ER+/HER2- Hi	7.578358
MB-5291	ER+/HER2- Hi	8.361707
MB-5292	ER+/HER2- Hi	7.881012
MB-5305	ER+/HER2- Hi	6.934152
MB-5306	ER+/HER2- Hi	7.929393
MB-5313	ER+/HER2- Hi	7.610225
MB-5317	ER+/HER2- Hi	6.832479
MB-5322	ER+/HER2- Hi	7.541605
MB-5328	ER+/HER2- Hi	7.627962
MB-5332	ER+/HER2- Hi	7.393211
MB-5334	ER+/HER2- Hi	7.325068
MB-5338	ER+/HER2- Hi	7.936133
MB-5339	ER+/HER2- Hi	7.075557
MB-5341	ER+/HER2- Hi	8.20497
MB-5347	ER+/HER2- Hi	7.375189
MB-5361	ER+/HER2- Hi	7.66408
MB-5370	ER+/HER2- Hi	7.333632
MB-5384	ER+/HER2- Hi	7.460939
MB-5386	ER+/HER2- Hi	7.573773
MB-5389	ER+/HER2- Hi	6.923491
MB-5396	ER+/HER2- Hi	7.661691
MB-5397	ER+/HER2- Hi	7.584073
MB-5402	ER+/HER2- Hi	7.21611
MB-5404	ER+/HER2- Hi	7.422775
MB-5405	ER+/HER2- Hi	7.822106

MB-5432	ER+/HER2- Hi	7.542165
MB-5433	ER+/HER2- Hi	8.374817
MB-5434	ER+/HER2- Hi	7.317509
MB-5435	ER+/HER2- Hi	7.200443
MB-5447	ER+/HER2- Hi	8.046706
MB-5454	ER+/HER2- Hi	7.151637
MB-5463	ER+/HER2- Hi	6.990406
MB-5470	ER+/HER2- Hi	7.496627
MB-5471	ER+/HER2- Hi	7.448012
MB-5477	ER+/HER2- Hi	7.575377
MB-5481	ER+/HER2- Hi	7.961646
MB-5485	ER+/HER2- Hi	7.306923
MB-5486	ER+/HER2- Hi	8.123825
MB-5491	ER+/HER2- Hi	7.390874
MB-5493	ER+/HER2- Hi	8.351828
MB-5497	ER+/HER2- Hi	7.645975
MB-5502	ER+/HER2- Hi	8.072976
MB-5505	ER+/HER2- Hi	7.951307
MB-5513	ER+/HER2- Hi	7.612621
MB-5518	ER+/HER2- Hi	7.679816
MB-5519	ER+/HER2- Hi	7.56965
MB-5520	ER+/HER2- Hi	7.567129
MB-5521	ER+/HER2- Hi	7.95582
MB-5525	ER+/HER2- Hi	7.32559
MB-5530	ER+/HER2- Hi	7.834407
MB-5532	ER+/HER2- Hi	8.084787
MB-5540	ER+/HER2- Hi	7.567993
MB-5550	ER+/HER2- Hi	7.383855
MB-5552	ER+/HER2- Hi	7.460833
MB-5553	ER+/HER2- Hi	7.895471
MB-5554	ER+/HER2- Hi	7.945222
MB-5556	ER+/HER2- Hi	7.989111
MB-5562	ER+/HER2- Hi	7.097787
MB-5575	ER+/HER2- Hi	7.886488
MB-5576	ER+/HER2- Hi	7.547266
MB-5577	ER+/HER2- Hi	7.382086
MB-5584	ER+/HER2- Hi	7.335111
MB-5590	ER+/HER2- Hi	7.541627
MB-5592	ER+/HER2- Hi	7.66971
MB-5604	ER+/HER2- Hi	8.065573
MB-5605	ER+/HER2- Hi	7.647303

MB-5613	ER+/HER2- Hi	7.245394
MB-5622	ER+/HER2- Hi	7.095884
MB-5623	ER+/HER2- Hi	7.838277
MB-5628	ER+/HER2- Hi	7.43163
MB-5629	ER+/HER2- Hi	7.235474
MB-5632	ER+/HER2- Hi	7.744001
MB-5636	ER+/HER2- Hi	7.316661
MB-5638	ER+/HER2- Hi	7.753341
MB-5641	ER+/HER2- Hi	7.483639
MB-5646	ER+/HER2- Hi	7.275293
MB-5647	ER+/HER2- Hi	7.709041
MB-5653	ER+/HER2- Hi	8.301518
MB-5654	ER+/HER2- Hi	7.283934
MB-6007	ER+/HER2- Hi	7.080921
MB-6008	ER+/HER2- Hi	7.26613
MB-6010	ER+/HER2- Hi	7.560571
MB-6011	ER+/HER2- Hi	8.052868
MB-6022	ER+/HER2- Hi	7.214994
MB-6023	ER+/HER2- Hi	6.860766
MB-6026	ER+/HER2- Hi	7.566647
MB-6039	ER+/HER2- Hi	7.578009
MB-6044	ER+/HER2- Hi	7.363335
MB-6047	ER+/HER2- Hi	8.199083
MB-6053	ER+/HER2- Hi	7.720476
MB-6059	ER+/HER2- Hi	7.633726
MB-6060	ER+/HER2- Hi	7.52966
MB-6071	ER+/HER2- Hi	7.476342
MB-6075	ER+/HER2- Hi	8.069828
MB-6077	ER+/HER2- Hi	7.211157
MB-6079	ER+/HER2- Hi	7.587537
MB-6080	ER+/HER2- Hi	7.507252
MB-6097	ER+/HER2- Hi	6.999224
MB-6105	ER+/HER2- Hi	7.3696
MB-6114	ER+/HER2- Hi	7.57992
MB-6124	ER+/HER2- Hi	7.941368
MB-6133	ER+/HER2- Hi	7.208212
MB-6135	ER+/HER2- Hi	7.577534
MB-6141	ER+/HER2- Hi	7.515154
MB-6145	ER+/HER2- Hi	8.200882
MB-6146	ER+/HER2- Hi	7.56102
MB-6149	ER+/HER2- Hi	8.07709

MB-6150	ER+/HER2- Hi	8.290472
MB-6154	ER+/HER2- Hi	7.302215
MB-6163	ER+/HER2- Hi	7.469995
MB-6164	ER+/HER2- Hi	7.288743
MB-6179	ER+/HER2- Hi	7.604937
MB-6181	ER+/HER2- Hi	6.746778
MB-6183	ER+/HER2- Hi	7.280482
MB-6184	ER+/HER2- Hi	7.192729
MB-6189	ER+/HER2- Hi	7.355773
MB-6192	ER+/HER2- Hi	8.81889
MB-6200	ER+/HER2- Hi	7.566169
MB-6201	ER+/HER2- Hi	7.791616
MB-6208	ER+/HER2- Hi	7.175825
MB-6217	ER+/HER2- Hi	7.895995
MB-6226	ER+/HER2- Hi	6.799567
MB-6239	ER+/HER2- Hi	7.605424
MB-6257	ER+/HER2- Hi	8.088452
MB-6302	ER+/HER2- Hi	7.265706
MB-6306	ER+/HER2- Hi	7.151524
MB-6312	ER+/HER2- Hi	7.237945
MB-6317	ER+/HER2- Hi	7.351246
MB-6322	ER+/HER2- Hi	7.990767
MB-6327	ER+/HER2- Hi	8.537321
MB-6328	ER+/HER2- Hi	7.13426
MB-6329	ER+/HER2- Hi	7.660887
MB-6344	ER+/HER2- Hi	7.309149
MB-6346	ER+/HER2- Hi	7.618367
MB-6359	ER+/HER2- Hi	7.874912
MB-7000	ER+/HER2- Hi	7.872295
MB-7002	ER+/HER2- Hi	6.913334
MB-7011	ER+/HER2- Hi	6.719444
MB-7014	ER+/HER2- Hi	7.001605
MB-7015	ER+/HER2- Hi	7.846866
MB-7018	ER+/HER2- Hi	6.592496
MB-7022	ER+/HER2- Hi	7.320624
MB-7024	ER+/HER2- Hi	7.238376
MB-7028	ER+/HER2- Hi	6.855755
MB-7042	ER+/HER2- Hi	6.705706
MB-7046	ER+/HER2- Hi	7.52264
MB-7048	ER+/HER2- Hi	7.814172
MB-7050	ER+/HER2- Hi	7.57992

MB-7051	ER+/HER2- Hi	8.091861
MB-7053	ER+/HER2- Hi	6.758905
MB-7065	ER+/HER2- Hi	7.359764
MB-7070	ER+/HER2- Hi	7.231557
MB-7071	ER+/HER2- Hi	7.128816
MB-7072	ER+/HER2- Hi	6.983205
MB-7075	ER+/HER2- Hi	7.485945
MB-7083	ER+/HER2- Hi	7.389261
MB-7086	ER+/HER2- Hi	6.800736
MB-7092	ER+/HER2- Hi	7.189308
MB-7094	ER+/HER2- Hi	7.551683
MB-7095	ER+/HER2- Hi	7.083769
MB-7097	ER+/HER2- Hi	7.493368
MB-7099	ER+/HER2- Hi	6.766269
MB-7100	ER+/HER2- Hi	7.31221
MB-7102	ER+/HER2- Hi	7.033082
MB-7107	ER+/HER2- Hi	7.423823
MB-7109	ER+/HER2- Hi	6.721995
MB-7111	ER+/HER2- Hi	7.066144
MB-7112	ER+/HER2- Hi	6.976418
MB-7116	ER+/HER2- Hi	7.696757
MB-7122	ER+/HER2- Hi	7.712541
MB-7124	ER+/HER2- Hi	7.273884
MB-7127	ER+/HER2- Hi	7.336965
MB-7131	ER+/HER2- Hi	7.373147
MB-7133	ER+/HER2- Hi	7.034707
MB-7138	ER+/HER2- Hi	6.964531
MB-7140	ER+/HER2- Hi	7.034707
MB-7142	ER+/HER2- Hi	8.006793
MB-7144	ER+/HER2- Hi	7.220911
MB-7147	ER+/HER2- Hi	7.555888
MB-7149	ER+/HER2- Hi	7.352162
MB-7150	ER+/HER2- Hi	7.136789
MB-7152	ER+/HER2- Hi	6.670218
MB-7153	ER+/HER2- Hi	7.361976
MB-7157	ER+/HER2- Hi	7.245304
MB-7160	ER+/HER2- Hi	7.054675
MB-7161	ER+/HER2- Hi	7.668256
MB-7171	ER+/HER2- Hi	8.551398
MB-7172	ER+/HER2- Hi	6.611195
MB-7173	ER+/HER2- Hi	7.144407

MB-7174	ER+/HER2- Hi	7.186769
MB-7176	ER+/HER2- Hi	7.013241
MB-7181	ER+/HER2- Hi	7.140995
MB-7182	ER+/HER2- Hi	7.490105
MB-7186	ER+/HER2- Hi	8.137396
MB-7193	ER+/HER2- Hi	6.951751
MB-7194	ER+/HER2- Hi	7.387914
MB-7196	ER+/HER2- Hi	7.210735
MB-7197	ER+/HER2- Hi	7.031487
MB-7198	ER+/HER2- Hi	7.463609
MB-7199	ER+/HER2- Hi	7.274318
MB-7200	ER+/HER2- Hi	7.136789
MB-7226	ER+/HER2- Hi	7.692411
MB-7227	ER+/HER2- Hi	7.276925
MB-7229	ER+/HER2- Hi	7.88912
MB-7230	ER+/HER2- Hi	7.517946
MB-7234	ER+/HER2- Hi	7.646353
MB-7237	ER+/HER2- Hi	6.605952
MB-7238	ER+/HER2- Hi	7.762986
MB-7253	ER+/HER2- Hi	7.731395
MB-7254	ER+/HER2- Hi	7.140592
MB-7262	ER+/HER2- Hi	7.564746
MB-7263	ER+/HER2- Hi	6.794313
MB-7268	ER+/HER2- Hi	7.541311
MB-7280	ER+/HER2- Hi	7.062903
MB-7283	ER+/HER2- Hi	7.84425
MB-7286	ER+/HER2- Hi	7.303078
MB-7287	ER+/HER2- Hi	6.872299
MB-7288	ER+/HER2- Hi	7.421557
MB-7292	ER+/HER2- Hi	7.475436
MB-7294	ER+/HER2- Hi	6.676953
MB-7298	ER+/HER2- Hi	7.483194
MB-7299	ER+/HER2- Hi	6.587989
MB-0036	ER+/HER2- Lc	6.931262
MB-0050	ER+/HER2- Lc	6.774453
MB-0101	ER+/HER2- Lc	7.196093
MB-0106	ER+/HER2- Lc	7.324199
MB-0111	ER+/HER2- Lc	7.145237
MB-0112	ER+/HER2- Lc	6.841047
MB-0117	ER+/HER2- Lc	7.71145
MB-0121	ER+/HER2- Lc	6.73756

MB-0122	ER+/HER2- Lc	7.017889
MB-0124	ER+/HER2- Lc	7.608808
MB-0125	ER+/HER2- Lc	7.453552
MB-0126	ER+/HER2- Lc	7.370213
MB-0127	ER+/HER2- Lc	7.596713
MB-0128	ER+/HER2- Lc	7.57992
MB-0133	ER+/HER2- Lc	7.252236
MB-0136	ER+/HER2- Lc	7.379545
MB-0138	ER+/HER2- Lc	7.680957
MB-0139	ER+/HER2- Lc	7.288411
MB-0142	ER+/HER2- Lc	7.296535
MB-0144	ER+/HER2- Lc	7.906936
MB-0145	ER+/HER2- Lc	7.360992
MB-0154	ER+/HER2- Lc	7.003357
MB-0155	ER+/HER2- Lc	7.422553
MB-0162	ER+/HER2- Lc	7.2912
MB-0166	ER+/HER2- Lc	7.625829
MB-0168	ER+/HER2- Lc	7.498508
MB-0170	ER+/HER2- Lc	6.914093
MB-0171	ER+/HER2- Lc	7.304259
MB-0172	ER+/HER2- Lc	7.234934
MB-0175	ER+/HER2- Lc	7.743428
MB-0177	ER+/HER2- Lc	7.062087
MB-0180	ER+/HER2- Lc	7.855265
MB-0181	ER+/HER2- Lc	7.168911
MB-0184	ER+/HER2- Lc	7.055838
MB-0189	ER+/HER2- Lc	7.617278
MB-0192	ER+/HER2- Lc	6.918569
MB-0194	ER+/HER2- Lc	7.353478
MB-0199	ER+/HER2- Lc	7.127757
MB-0204	ER+/HER2- Lc	7.272283
MB-0205	ER+/HER2- Lc	7.075136
MB-0207	ER+/HER2- Lc	7.080089
MB-0211	ER+/HER2- Lc	7.43758
MB-0222	ER+/HER2- Lc	7.156941
MB-0223	ER+/HER2- Lc	7.707437
MB-0224	ER+/HER2- Lc	7.026796
MB-0226	ER+/HER2- Lc	7.490105
MB-0227	ER+/HER2- Lc	6.888481
MB-0228	ER+/HER2- Lc	7.064948
MB-0229	ER+/HER2- Lc	7.147766

MB-0231	ER+/HER2- Lc	7.31091
MB-0232	ER+/HER2- Lc	6.826292
MB-0233	ER+/HER2- Lc	7.27916
MB-0235	ER+/HER2- Lc	7.089083
MB-0239	ER+/HER2- Lc	6.852541
MB-0242	ER+/HER2- Lc	6.804085
MB-0243	ER+/HER2- Lc	6.883628
MB-0245	ER+/HER2- Lc	7.053883
MB-0247	ER+/HER2- Lc	7.039864
MB-0248	ER+/HER2- Lc	7.186006
MB-0253	ER+/HER2- Lc	7.078815
MB-0260	ER+/HER2- Lc	6.907464
MB-0263	ER+/HER2- Lc	6.896998
MB-0264	ER+/HER2- Lc	6.537326
MB-0273	ER+/HER2- Lc	6.762964
MB-0275	ER+/HER2- Lc	7.096081
MB-0279	ER+/HER2- Lc	6.983997
MB-0280	ER+/HER2- Lc	7.545538
MB-0282	ER+/HER2- Lc	7.171604
MB-0288	ER+/HER2- Lc	7.306566
MB-0290	ER+/HER2- Lc	7.134677
MB-0295	ER+/HER2- Lc	6.631249
MB-0301	ER+/HER2- Lc	7.104024
MB-0302	ER+/HER2- Lc	6.698238
MB-0305	ER+/HER2- Lc	7.071496
MB-0308	ER+/HER2- Lc	7.157811
MB-0309	ER+/HER2- Lc	7.187189
MB-0310	ER+/HER2- Lc	7.012857
MB-0315	ER+/HER2- Lc	7.359214
MB-0317	ER+/HER2- Lc	7.09964
MB-0320	ER+/HER2- Lc	6.991073
MB-0322	ER+/HER2- Lc	7.071679
MB-0339	ER+/HER2- Lc	6.530286
MB-0342	ER+/HER2- Lc	7.356204
MB-0344	ER+/HER2- Lc	6.794915
MB-0345	ER+/HER2- Lc	7.427801
MB-0348	ER+/HER2- Lc	6.792229
MB-0353	ER+/HER2- Lc	7.626883
MB-0359	ER+/HER2- Lc	7.301611
MB-0364	ER+/HER2- Lc	6.669404
MB-0377	ER+/HER2- Lc	6.894176

MB-0379	ER+/HER2- Lc	6.935089
MB-0382	ER+/HER2- Lc	7.190469
MB-0386	ER+/HER2- Lc	7.980899
MB-0388	ER+/HER2- Lc	7.527768
MB-0397	ER+/HER2- Lc	7.781698
MB-0402	ER+/HER2- Lc	7.317509
MB-0410	ER+/HER2- Lc	7.114047
MB-0411	ER+/HER2- Lc	6.965915
MB-0419	ER+/HER2- Lc	6.564386
MB-0422	ER+/HER2- Lc	7.458341
MB-0425	ER+/HER2- Lc	7.013192
MB-0426	ER+/HER2- Lc	7.203893
MB-0431	ER+/HER2- Lc	6.969529
MB-0442	ER+/HER2- Lc	7.030661
MB-0443	ER+/HER2- Lc	7.04081
MB-0444	ER+/HER2- Lc	7.615327
MB-0449	ER+/HER2- Lc	7.254902
MB-0451	ER+/HER2- Lc	6.484965
MB-0454	ER+/HER2- Lc	7.198678
MB-0458	ER+/HER2- Lc	7.368707
MB-0460	ER+/HER2- Lc	7.410245
MB-0463	ER+/HER2- Lc	7.426194
MB-0478	ER+/HER2- Lc	6.902475
MB-0480	ER+/HER2- Lc	7.224624
MB-0486	ER+/HER2- Lc	7.333877
MB-0487	ER+/HER2- Lc	7.195297
MB-0490	ER+/HER2- Lc	7.039568
MB-0496	ER+/HER2- Lc	6.955905
MB-0497	ER+/HER2- Lc	7.678382
MB-0501	ER+/HER2- Lc	6.721739
MB-0503	ER+/HER2- Lc	6.775334
MB-0504	ER+/HER2- Lc	7.233991
MB-0505	ER+/HER2- Lc	7.464942
MB-0507	ER+/HER2- Lc	7.460688
MB-0511	ER+/HER2- Lc	7.584761
MB-0513	ER+/HER2- Lc	6.991007
MB-0517	ER+/HER2- Lc	7.577402
MB-0519	ER+/HER2- Lc	7.233764
MB-0521	ER+/HER2- Lc	6.808849
MB-0524	ER+/HER2- Lc	7.669558
MB-0527	ER+/HER2- Lc	7.101531

MB-0528	ER+/HER2- Lc	6.938664
MB-0535	ER+/HER2- Lc	7.017244
MB-0537	ER+/HER2- Lc	7.72144
MB-0544	ER+/HER2- Lc	6.927027
MB-0551	ER+/HER2- Lc	7.185909
MB-0554	ER+/HER2- Lc	7.091972
MB-0568	ER+/HER2- Lc	6.849486
MB-0573	ER+/HER2- Lc	7.654473
MB-0575	ER+/HER2- Lc	7.136683
MB-0578	ER+/HER2- Lc	6.885385
MB-0579	ER+/HER2- Lc	6.694071
MB-0583	ER+/HER2- Lc	7.253215
MB-0586	ER+/HER2- Lc	7.370922
MB-0588	ER+/HER2- Lc	6.931327
MB-0596	ER+/HER2- Lc	7.305533
MB-0597	ER+/HER2- Lc	6.93308
MB-0599	ER+/HER2- Lc	7.940554
MB-0603	ER+/HER2- Lc	6.233423
MB-0605	ER+/HER2- Lc	6.73274
MB-0610	ER+/HER2- Lc	6.940022
MB-0618	ER+/HER2- Lc	7.049302
MB-0621	ER+/HER2- Lc	7.390797
MB-0624	ER+/HER2- Lc	7.168207
MB-0631	ER+/HER2- Lc	7.21865
MB-0632	ER+/HER2- Lc	7.363282
MB-0640	ER+/HER2- Lc	7.216288
MB-0641	ER+/HER2- Lc	7.178386
MB-0642	ER+/HER2- Lc	7.133393
MB-0644	ER+/HER2- Lc	7.156433
MB-0649	ER+/HER2- Lc	7.195269
MB-0654	ER+/HER2- Lc	6.951748
MB-0655	ER+/HER2- Lc	6.952149
MB-0657	ER+/HER2- Lc	7.083114
MB-0661	ER+/HER2- Lc	6.568932
MB-0872	ER+/HER2- Lc	7.146876
MB-0880	ER+/HER2- Lc	7.236407
MB-0891	ER+/HER2- Lc	7.264837
MB-0897	ER+/HER2- Lc	7.358879
MB-0904	ER+/HER2- Lc	7.489524
MB-2536	ER+/HER2- Lc	6.679755
MB-2564	ER+/HER2- Lc	6.435078

MB-2610	ER+/HER2- Lc	6.686564
MB-2614	ER+/HER2- Lc	6.574655
MB-2616	ER+/HER2- Lc	6.770344
MB-2624	ER+/HER2- Lc	6.958482
MB-2669	ER+/HER2- Lc	7.053609
MB-2711	ER+/HER2- Lc	6.843063
MB-2712	ER+/HER2- Lc	6.711829
MB-2725	ER+/HER2- Lc	7.148144
MB-2744	ER+/HER2- Lc	7.193981
MB-2747	ER+/HER2- Lc	7.198213
MB-2749	ER+/HER2- Lc	7.009545
MB-2750	ER+/HER2- Lc	7.633162
MB-2752	ER+/HER2- Lc	7.482432
MB-2754	ER+/HER2- Lc	6.86182
MB-2760	ER+/HER2- Lc	6.951542
MB-2765	ER+/HER2- Lc	6.74306
MB-2770	ER+/HER2- Lc	7.021679
MB-2791	ER+/HER2- Lc	7.067661
MB-2795	ER+/HER2- Lc	6.82517
MB-2815	ER+/HER2- Lc	7.20002
MB-2819	ER+/HER2- Lc	6.63404
MB-2823	ER+/HER2- Lc	6.84008
MB-2835	ER+/HER2- Lc	6.974823
MB-2840	ER+/HER2- Lc	6.841797
MB-2843	ER+/HER2- Lc	6.431235
MB-2845	ER+/HER2- Lc	6.470194
MB-2848	ER+/HER2- Lc	6.510734
MB-2851	ER+/HER2- Lc	6.774516
MB-2854	ER+/HER2- Lc	7.219968
MB-2863	ER+/HER2- Lc	7.081133
MB-2867	ER+/HER2- Lc	6.82803
MB-2896	ER+/HER2- Lc	6.937218
MB-2900	ER+/HER2- Lc	6.884624
MB-2916	ER+/HER2- Lc	6.956472
MB-2919	ER+/HER2- Lc	7.039568
MB-2931	ER+/HER2- Lc	6.44405
MB-2933	ER+/HER2- Lc	6.875285
MB-2944	ER+/HER2- Lc	6.862673
MB-2947	ER+/HER2- Lc	6.648073
MB-2960	ER+/HER2- Lc	6.536718
MB-2969	ER+/HER2- Lc	6.681799

MB-2970	ER+/HER2- Lc	6.774859
MB-2971	ER+/HER2- Lc	6.63716
MB-2977	ER+/HER2- Lc	6.70398
MB-2990	ER+/HER2- Lc	7.126752
MB-2996	ER+/HER2- Lc	7.126586
MB-3005	ER+/HER2- Lc	6.479476
MB-3008	ER+/HER2- Lc	7.469726
MB-3013	ER+/HER2- Lc	6.583376
MB-3032	ER+/HER2- Lc	6.503815
MB-3033	ER+/HER2- Lc	7.124724
MB-3035	ER+/HER2- Lc	7.535157
MB-3037	ER+/HER2- Lc	7.022926
MB-3049	ER+/HER2- Lc	6.97954
MB-3064	ER+/HER2- Lc	7.07641
MB-3079	ER+/HER2- Lc	7.011975
MB-3085	ER+/HER2- Lc	7.013018
MB-3105	ER+/HER2- Lc	7.595259
MB-3110	ER+/HER2- Lc	7.483461
MB-3121	ER+/HER2- Lc	7.019562
MB-3171	ER+/HER2- Lc	6.817704
MB-3222	ER+/HER2- Lc	6.538706
MB-3228	ER+/HER2- Lc	7.130927
MB-3252	ER+/HER2- Lc	7.131324
MB-3254	ER+/HER2- Lc	6.82287
MB-3295	ER+/HER2- Lc	7.132986
MB-3298	ER+/HER2- Lc	6.708765
MB-3328	ER+/HER2- Lc	6.789392
MB-3351	ER+/HER2- Lc	7.89844
MB-3365	ER+/HER2- Lc	7.654236
MB-3378	ER+/HER2- Lc	7.069323
MB-3381	ER+/HER2- Lc	7.741473
MB-3403	ER+/HER2- Lc	7.511412
MB-3412	ER+/HER2- Lc	7.460029
MB-3429	ER+/HER2- Lc	7.279183
MB-3439	ER+/HER2- Lc	7.450437
MB-3450	ER+/HER2- Lc	6.982545
MB-3452	ER+/HER2- Lc	7.222928
MB-3462	ER+/HER2- Lc	7.229006
MB-3510	ER+/HER2- Lc	7.175392
MB-3526	ER+/HER2- Lc	6.819542
MB-3545	ER+/HER2- Lc	6.937497

MB-3707	ER+/HER2- Lc	7.150305
MB-3711	ER+/HER2- Lc	6.864793
MB-3748	ER+/HER2- Lc	6.805233
MB-3781	ER+/HER2- Lc	7.19432
MB-3850	ER+/HER2- Lc	7.022218
MB-3854	ER+/HER2- Lc	7.439348
MB-3865	ER+/HER2- Lc	6.986224
MB-3871	ER+/HER2- Lc	7.535487
MB-3874	ER+/HER2- Lc	7.955341
MB-4000	ER+/HER2- Lc	7.450496
MB-4012	ER+/HER2- Lc	7.779824
MB-4018	ER+/HER2- Lc	6.933289
MB-4120	ER+/HER2- Lc	7.541629
MB-4140	ER+/HER2- Lc	7.713472
MB-4141	ER+/HER2- Lc	7.562896
MB-4145	ER+/HER2- Lc	7.50402
MB-4173	ER+/HER2- Lc	6.949774
MB-4212	ER+/HER2- Lc	7.665343
MB-4230	ER+/HER2- Lc	7.658739
MB-4283	ER+/HER2- Lc	7.278703
MB-4293	ER+/HER2- Lc	7.336925
MB-4342	ER+/HER2- Lc	7.79672
MB-4343	ER+/HER2- Lc	7.862439
MB-4357	ER+/HER2- Lc	8.019562
MB-4529	ER+/HER2- Lc	7.200875
MB-4599	ER+/HER2- Lc	7.061671
MB-4616	ER+/HER2- Lc	7.710902
MB-4633	ER+/HER2- Lc	7.790056
MB-4641	ER+/HER2- Lc	7.759356
MB-4653	ER+/HER2- Lc	8.249279
MB-4661	ER+/HER2- Lc	7.762341
MB-4665	ER+/HER2- Lc	7.373334
MB-4670	ER+/HER2- Lc	6.938568
MB-4674	ER+/HER2- Lc	6.495719
MB-4675	ER+/HER2- Lc	6.876517
MB-4681	ER+/HER2- Lc	7.537104
MB-4686	ER+/HER2- Lc	7.707053
MB-4691	ER+/HER2- Lc	7.453646
MB-4692	ER+/HER2- Lc	7.699696
MB-4695	ER+/HER2- Lc	7.315788
MB-4697	ER+/HER2- Lc	7.156118

MB-4698	ER+/HER2- Lc	7.193622
MB-4701	ER+/HER2- Lc	7.051848
MB-4704	ER+/HER2- Lc	7.725257
MB-4705	ER+/HER2- Lc	7.670217
MB-4706	ER+/HER2- Lc	6.795259
MB-4708	ER+/HER2- Lc	7.454117
MB-4709	ER+/HER2- Lc	7.588488
MB-4710	ER+/HER2- Lc	7.412514
MB-4716	ER+/HER2- Lc	7.178033
MB-4718	ER+/HER2- Lc	7.555681
MB-4719	ER+/HER2- Lc	7.060161
MB-4721	ER+/HER2- Lc	7.530514
MB-4722	ER+/HER2- Lc	7.265706
MB-4730	ER+/HER2- Lc	7.108693
MB-4735	ER+/HER2- Lc	7.897158
MB-4738	ER+/HER2- Lc	7.823506
MB-4739	ER+/HER2- Lc	7.133393
MB-4741	ER+/HER2- Lc	7.465533
MB-4742	ER+/HER2- Lc	7.310023
MB-4743	ER+/HER2- Lc	7.691346
MB-4752	ER+/HER2- Lc	8.546868
MB-4760	ER+/HER2- Lc	7.781876
MB-4762	ER+/HER2- Lc	7.691164
MB-4764	ER+/HER2- Lc	7.31711
MB-4771	ER+/HER2- Lc	7.474774
MB-4778	ER+/HER2- Lc	7.495652
MB-4779	ER+/HER2- Lc	7.541287
MB-4784	ER+/HER2- Lc	8.129807
MB-4785	ER+/HER2- Lc	7.588391
MB-4806	ER+/HER2- Lc	6.945346
MB-4814	ER+/HER2- Lc	8.323382
MB-4818	ER+/HER2- Lc	7.214767
MB-4820	ER+/HER2- Lc	7.785486
MB-4822	ER+/HER2- Lc	7.658852
MB-4825	ER+/HER2- Lc	7.102795
MB-4827	ER+/HER2- Lc	7.350541
MB-4832	ER+/HER2- Lc	7.652492
MB-4843	ER+/HER2- Lc	7.83128
MB-4845	ER+/HER2- Lc	7.736386
MB-4853	ER+/HER2- Lc	7.180852
MB-4855	ER+/HER2- Lc	7.130138

MB-4860	ER+/HER2- Lc	7.878063
MB-4867	ER+/HER2- Lc	7.228855
MB-4869	ER+/HER2- Lc	7.359048
MB-4870	ER+/HER2- Lc	7.519334
MB-4872	ER+/HER2- Lc	7.87292
MB-4882	ER+/HER2- Lc	8.34753
MB-4883	ER+/HER2- Lc	7.662924
MB-4887	ER+/HER2- Lc	8.045639
MB-4897	ER+/HER2- Lc	7.575199
MB-4898	ER+/HER2- Lc	7.295151
MB-4899	ER+/HER2- Lc	7.506069
MB-4941	ER+/HER2- Lc	7.891384
MB-4950	ER+/HER2- Lc	7.716981
MB-4959	ER+/HER2- Lc	7.309463
MB-4961	ER+/HER2- Lc	7.311293
MB-4962	ER+/HER2- Lc	7.676548
MB-4966	ER+/HER2- Lc	7.790479
MB-4967	ER+/HER2- Lc	7.38483
MB-4968	ER+/HER2- Lc	6.687311
MB-4977	ER+/HER2- Lc	7.323473
MB-4978	ER+/HER2- Lc	7.287888
MB-4981	ER+/HER2- Lc	7.432938
MB-4987	ER+/HER2- Lc	7.309569
MB-4992	ER+/HER2- Lc	7.56951
MB-4996	ER+/HER2- Lc	6.99444
MB-4999	ER+/HER2- Lc	7.470442
MB-5004	ER+/HER2- Lc	7.399305
MB-5011	ER+/HER2- Lc	7.176658
MB-5013	ER+/HER2- Lc	6.883131
MB-5015	ER+/HER2- Lc	6.935372
MB-5020	ER+/HER2- Lc	7.445428
MB-5027	ER+/HER2- Lc	7.604758
MB-5043	ER+/HER2- Lc	7.023354
MB-5050	ER+/HER2- Lc	7.807029
MB-5053	ER+/HER2- Lc	7.212076
MB-5059	ER+/HER2- Lc	7.167328
MB-5064	ER+/HER2- Lc	7.379103
MB-5066	ER+/HER2- Lc	7.098212
MB-5068	ER+/HER2- Lc	7.632299
MB-5073	ER+/HER2- Lc	7.483194
MB-5079	ER+/HER2- Lc	6.876517

MB-5084	ER+/HER2- Lc	7.336885
MB-5088	ER+/HER2- Lc	7.883133
MB-5092	ER+/HER2- Lc	7.193981
MB-5110	ER+/HER2- Lc	7.766011
MB-5113	ER+/HER2- Lc	7.858793
MB-5118	ER+/HER2- Lc	7.643842
MB-5119	ER+/HER2- Lc	7.623158
MB-5122	ER+/HER2- Lc	7.110608
MB-5123	ER+/HER2- Lc	6.807725
MB-5134	ER+/HER2- Lc	7.855662
MB-5143	ER+/HER2- Lc	7.499326
MB-5150	ER+/HER2- Lc	7.658963
MB-5161	ER+/HER2- Lc	7.165676
MB-5163	ER+/HER2- Lc	7.680534
MB-5171	ER+/HER2- Lc	7.259564
MB-5175	ER+/HER2- Lc	7.938701
MB-5178	ER+/HER2- Lc	7.546488
MB-5179	ER+/HER2- Lc	7.275358
MB-5182	ER+/HER2- Lc	7.392249
MB-5183	ER+/HER2- Lc	7.261445
MB-5184	ER+/HER2- Lc	7.95357
MB-5185	ER+/HER2- Lc	7.670111
MB-5189	ER+/HER2- Lc	7.985337
MB-5201	ER+/HER2- Lc	7.873388
MB-5204	ER+/HER2- Lc	7.217941
MB-5206	ER+/HER2- Lc	7.359574
MB-5214	ER+/HER2- Lc	7.793692
MB-5221	ER+/HER2- Lc	7.382025
MB-5224	ER+/HER2- Lc	7.279233
MB-5230	ER+/HER2- Lc	7.337105
MB-5240	ER+/HER2- Lc	7.024764
MB-5251	ER+/HER2- Lc	7.288315
MB-5253	ER+/HER2- Lc	6.719613
MB-5264	ER+/HER2- Lc	6.807428
MB-5267	ER+/HER2- Lc	7.34097
MB-5268	ER+/HER2- Lc	7.389015
MB-5277	ER+/HER2- Lc	7.89652
MB-5278	ER+/HER2- Lc	7.692906
MB-5281	ER+/HER2- Lc	7.759807
MB-5284	ER+/HER2- Lc	7.072999
MB-5287	ER+/HER2- Lc	7.237495

MB-5290	ER+/HER2- Lc	6.974383
MB-5293	ER+/HER2- Lc	7.697077
MB-5300	ER+/HER2- Lc	7.65103
MB-5308	ER+/HER2- Lc	7.13892
MB-5310	ER+/HER2- Lc	7.235324
MB-5324	ER+/HER2- Lc	7.11717
MB-5329	ER+/HER2- Lc	7.484739
MB-5330	ER+/HER2- Lc	7.499048
MB-5345	ER+/HER2- Lc	7.420636
MB-5358	ER+/HER2- Lc	6.87989
MB-5360	ER+/HER2- Lc	6.660783
MB-5364	ER+/HER2- Lc	7.004781
MB-5365	ER+/HER2- Lc	7.439674
MB-5368	ER+/HER2- Lc	7.163673
MB-5369	ER+/HER2- Lc	7.415708
MB-5373	ER+/HER2- Lc	6.97161
MB-5377	ER+/HER2- Lc	7.557849
MB-5382	ER+/HER2- Lc	7.021639
MB-5383	ER+/HER2- Lc	6.993505
MB-5388	ER+/HER2- Lc	6.527903
MB-5393	ER+/HER2- Lc	7.238627
MB-5395	ER+/HER2- Lc	6.870745
MB-5398	ER+/HER2- Lc	7.123107
MB-5399	ER+/HER2- Lc	7.670217
MB-5401	ER+/HER2- Lc	7.586581
MB-5403	ER+/HER2- Lc	7.118185
MB-5407	ER+/HER2- Lc	7.474975
MB-5410	ER+/HER2- Lc	7.547441
MB-5412	ER+/HER2- Lc	7.690237
MB-5422	ER+/HER2- Lc	8.148776
MB-5424	ER+/HER2- Lc	7.862035
MB-5425	ER+/HER2- Lc	7.674681
MB-5428	ER+/HER2- Lc	7.657481
MB-5429	ER+/HER2- Lc	7.471674
MB-5441	ER+/HER2- Lc	7.253933
MB-5444	ER+/HER2- Lc	7.521681
MB-5451	ER+/HER2- Lc	6.925131
MB-5455	ER+/HER2- Lc	7.186584
MB-5457	ER+/HER2- Lc	7.371988
MB-5464	ER+/HER2- Lc	7.492879
MB-5472	ER+/HER2- Lc	7.553813

MB-5473	ER+/HER2- Lc	7.466755
MB-5475	ER+/HER2- Lc	6.922544
MB-5478	ER+/HER2- Lc	7.540608
MB-5484	ER+/HER2- Lc	6.706813
MB-5489	ER+/HER2- Lc	7.71295
MB-5490	ER+/HER2- Lc	7.685232
MB-5492	ER+/HER2- Lc	7.374513
MB-5495	ER+/HER2- Lc	7.945656
MB-5499	ER+/HER2- Lc	7.882657
MB-5510	ER+/HER2- Lc	7.208281
MB-5514	ER+/HER2- Lc	7.258642
MB-5533	ER+/HER2- Lc	7.345054
MB-5541	ER+/HER2- Lc	7.151199
MB-5543	ER+/HER2- Lc	7.699202
MB-5563	ER+/HER2- Lc	7.827173
MB-5567	ER+/HER2- Lc	7.519171
MB-5571	ER+/HER2- Lc	7.189823
MB-5579	ER+/HER2- Lc	7.431378
MB-5580	ER+/HER2- Lc	7.640432
MB-5582	ER+/HER2- Lc	7.811454
MB-5583	ER+/HER2- Lc	7.259988
MB-5589	ER+/HER2- Lc	7.600473
MB-5591	ER+/HER2- Lc	7.893489
MB-5596	ER+/HER2- Lc	7.336601
MB-5597	ER+/HER2- Lc	7.285545
MB-5599	ER+/HER2- Lc	7.755574
MB-5601	ER+/HER2- Lc	7.589506
MB-5603	ER+/HER2- Lc	7.682944
MB-5614	ER+/HER2- Lc	7.411268
MB-5617	ER+/HER2- Lc	7.210128
MB-5620	ER+/HER2- Lc	7.101087
MB-5626	ER+/HER2- Lc	7.546115
MB-5635	ER+/HER2- Lc	7.471735
MB-5640	ER+/HER2- Lc	7.50402
MB-5642	ER+/HER2- Lc	7.831921
MB-5645	ER+/HER2- Lc	7.661314
MB-5656	ER+/HER2- Lc	7.901313
MB-6001	ER+/HER2- Lc	7.521681
MB-6006	ER+/HER2- Lc	7.421092
MB-6014	ER+/HER2- Lc	7.040784
MB-6016	ER+/HER2- Lc	7.775632

MB-6017	ER+/HER2- Lc	7.63855
MB-6018	ER+/HER2- Lc	6.994825
MB-6019	ER+/HER2- Lc	7.641001
MB-6021	ER+/HER2- Lc	7.02008
MB-6024	ER+/HER2- Lc	7.510925
MB-6029	ER+/HER2- Lc	7.293535
MB-6030	ER+/HER2- Lc	7.343252
MB-6042	ER+/HER2- Lc	7.489604
MB-6050	ER+/HER2- Lc	7.926861
MB-6051	ER+/HER2- Lc	7.314065
MB-6065	ER+/HER2- Lc	7.403533
MB-6069	ER+/HER2- Lc	7.052626
MB-6082	ER+/HER2- Lc	7.328591
MB-6083	ER+/HER2- Lc	7.765512
MB-6092	ER+/HER2- Lc	6.596639
MB-6103	ER+/HER2- Lc	7.462227
MB-6107	ER+/HER2- Lc	7.544569
MB-6108	ER+/HER2- Lc	7.596713
MB-6118	ER+/HER2- Lc	7.340555
MB-6125	ER+/HER2- Lc	7.391482
MB-6138	ER+/HER2- Lc	7.973299
MB-6147	ER+/HER2- Lc	7.747434
MB-6167	ER+/HER2- Lc	7.395477
MB-6168	ER+/HER2- Lc	7.398109
MB-6171	ER+/HER2- Lc	7.521681
MB-6185	ER+/HER2- Lc	7.836462
MB-6190	ER+/HER2- Lc	8.264613
MB-6194	ER+/HER2- Lc	7.515154
MB-6195	ER+/HER2- Lc	7.601555
MB-6204	ER+/HER2- Lc	7.657481
MB-6207	ER+/HER2- Lc	7.820392
MB-6211	ER+/HER2- Lc	7.428894
MB-6212	ER+/HER2- Lc	7.496171
MB-6214	ER+/HER2- Lc	7.456273
MB-6218	ER+/HER2- Lc	7.72292
MB-6225	ER+/HER2- Lc	7.539906
MB-6229	ER+/HER2- Lc	7.48139
MB-6231	ER+/HER2- Lc	7.84425
MB-6232	ER+/HER2- Lc	7.988028
MB-6233	ER+/HER2- Lc	7.745419
MB-6234	ER+/HER2- Lc	7.593792

MB-6238	ER+/HER2- Lc	7.054675
MB-6253	ER+/HER2- Lc	7.589946
MB-6254	ER+/HER2- Lc	7.260007
MB-6256	ER+/HER2- Lc	7.524017
MB-6308	ER+/HER2- Lc	7.286584
MB-6319	ER+/HER2- Lc	7.559636
MB-7003	ER+/HER2- Lc	7.158636
MB-7004	ER+/HER2- Lc	6.943497
MB-7005	ER+/HER2- Lc	6.859587
MB-7006	ER+/HER2- Lc	7.105653
MB-7010	ER+/HER2- Lc	6.780055
MB-7013	ER+/HER2- Lc	7.167025
MB-7019	ER+/HER2- Lc	6.963369
MB-7026	ER+/HER2- Lc	7.05795
MB-7029	ER+/HER2- Lc	7.457668
MB-7032	ER+/HER2- Lc	7.014853
MB-7034	ER+/HER2- Lc	6.708241
MB-7037	ER+/HER2- Lc	6.6429
MB-7040	ER+/HER2- Lc	7.113933
MB-7041	ER+/HER2- Lc	7.215417
MB-7043	ER+/HER2- Lc	7.246168
MB-7044	ER+/HER2- Lc	7.421092
MB-7056	ER+/HER2- Lc	7.480019
MB-7057	ER+/HER2- Lc	6.950554
MB-7058	ER+/HER2- Lc	6.676248
MB-7060	ER+/HER2- Lc	7.305699
MB-7061	ER+/HER2- Lc	7.320192
MB-7062	ER+/HER2- Lc	6.936829
MB-7063	ER+/HER2- Lc	6.996418
MB-7066	ER+/HER2- Lc	7.422457
MB-7074	ER+/HER2- Lc	7.169519
MB-7076	ER+/HER2- Lc	7.393211
MB-7077	ER+/HER2- Lc	7.068213
MB-7080	ER+/HER2- Lc	6.584549
MB-7085	ER+/HER2- Lc	6.543367
MB-7091	ER+/HER2- Lc	7.614985
MB-7093	ER+/HER2- Lc	6.812792
MB-7101	ER+/HER2- Lc	7.231134
MB-7106	ER+/HER2- Lc	7.040369
MB-7113	ER+/HER2- Lc	6.758905
MB-7118	ER+/HER2- Lc	7.127166

MB-7123	ER+/HER2- Lc	7.286584
MB-7132	ER+/HER2- Lc	6.856521
MB-7137	ER+/HER2- Lc	6.923887
MB-7141	ER+/HER2- Lc	7.041582
MB-7162	ER+/HER2- Lc	6.932112
MB-7163	ER+/HER2- Lc	7.380274
MB-7164	ER+/HER2- Lc	7.081334
MB-7167	ER+/HER2- Lc	6.88075
MB-7168	ER+/HER2- Lc	6.883094
MB-7185	ER+/HER2- Lc	7.077166
MB-7195	ER+/HER2- Lc	7.074328
MB-7212	ER+/HER2- Lc	6.86155
MB-7215	ER+/HER2- Lc	6.912935
MB-7216	ER+/HER2- Lc	7.338318
MB-7217	ER+/HER2- Lc	6.725213
MB-7218	ER+/HER2- Lc	7.031487
MB-7219	ER+/HER2- Lc	7.050548
MB-7220	ER+/HER2- Lc	7.107751
MB-7228	ER+/HER2- Lc	6.85
MB-7231	ER+/HER2- Lc	7.030252
MB-7232	ER+/HER2- Lc	7.018855
MB-7233	ER+/HER2- Lc	6.474864
MB-7235	ER+/HER2- Lc	7.293535
MB-7236	ER+/HER2- Lc	6.775941
MB-7241	ER+/HER2- Lc	6.407379
MB-7243	ER+/HER2- Lc	7.183373
MB-7244	ER+/HER2- Lc	6.940732
MB-7249	ER+/HER2- Lc	7.245723
MB-7264	ER+/HER2- Lc	7.318836
MB-7276	ER+/HER2- Lc	7.437116
MB-7277	ER+/HER2- Lc	6.470648
MB-7278	ER+/HER2- Lc	6.755221
MB-7284	ER+/HER2- Lc	7.374062
MB-7285	ER+/HER2- Lc	6.930172
MB-7289	ER+/HER2- Lc	7.226912
MB-7293	ER+/HER2- Lc	7.143147
MB-7295	ER+/HER2- Lc	6.765162
MB-0113	HER2+	7.536208
MB-0129	HER2+	8.006231
MB-0130	HER2+	7.523567
MB-0148	HER2+	7.376333

MB-0152	HER2+	7.574901
MB-0165	HER2+	7.66911
MB-0201	HER2+	7.52627
MB-0220	HER2+	7.552488
MB-0225	HER2+	7.93341
MB-0230	HER2+	6.896603
MB-0236	HER2+	7.416166
MB-0291	HER2+	7.366319
MB-0294	HER2+	7.457127
MB-0307	HER2+	6.973352
MB-0314	HER2+	7.572266
MB-0346	HER2+	7.2746
MB-0361	HER2+	7.349887
MB-0371	HER2+	7.088382
MB-0373	HER2+	7.562289
MB-0378	HER2+	6.893906
MB-0381	HER2+	7.038371
MB-0389	HER2+	7.034683
MB-0395	HER2+	6.951246
MB-0421	HER2+	7.12062
MB-0434	HER2+	7.646397
MB-0438	HER2+	7.655764
MB-0462	HER2+	7.083379
MB-0465	HER2+	7.184102
MB-0467	HER2+	7.651315
MB-0479	HER2+	7.419283
MB-0482	HER2+	7.163155
MB-0552	HER2+	7.033896
MB-0593	HER2+	7.1933
MB-0615	HER2+	7.489127
MB-0656	HER2+	7.30235
MB-0662	HER2+	7.60961
MB-0663	HER2+	7.83854
MB-0895	HER2+	7.410124
MB-2513	HER2+	7.081733
MB-2517	HER2+	6.772255
MB-2626	HER2+	7.498993
MB-2632	HER2+	7.110394
MB-2735	HER2+	8.208898
MB-2742	HER2+	7.56283
MB-2758	HER2+	7.660887

MB-2786	HER2+	7.188777
MB-2844	HER2+	7.031804
MB-2847	HER2+	7.528534
MB-2895	HER2+	7.080921
MB-2923	HER2+	7.952145
MB-2964	HER2+	6.969574
MB-2983	HER2+	7.506739
MB-2984	HER2+	7.392675
MB-2994	HER2+	6.961581
MB-3025	HER2+	8.095414
MB-3103	HER2+	7.987515
MB-3122	HER2+	7.375172
MB-3272	HER2+	7.496384
MB-3329	HER2+	7.592273
MB-3355	HER2+	7.652058
MB-3360	HER2+	7.266823
MB-3361	HER2+	8.107543
MB-3379	HER2+	7.57665
MB-3382	HER2+	7.755374
MB-3386	HER2+	7.777244
MB-3435	HER2+	7.017244
MB-3467	HER2+	7.471437
MB-3470	HER2+	6.945318
MB-3488	HER2+	7.621118
MB-3497	HER2+	7.203484
MB-3528	HER2+	7.522125
MB-3606	HER2+	7.245854
MB-3866	HER2+	7.727877
MB-3978	HER2+	7.592395
MB-4127	HER2+	8.445233
MB-4270	HER2+	7.391911
MB-4276	HER2+	7.531977
MB-4643	HER2+	7.636601
MB-4644	HER2+	7.995138
MB-4663	HER2+	8.031167
MB-4688	HER2+	7.298262
MB-4724	HER2+	7.710432
MB-4725	HER2+	7.504936
MB-4729	HER2+	8.341324
MB-4731	HER2+	7.715507
MB-4745	HER2+	8.172286

MB-4763	HER2+	8.380303
MB-4796	HER2+	7.330933
MB-4846	HER2+	8.001786
MB-4858	HER2+	7.720726
MB-4866	HER2+	8.331697
MB-4871	HER2+	8.365581
MB-4878	HER2+	7.970144
MB-4879	HER2+	7.64541
MB-4886	HER2+	8.199878
MB-4896	HER2+	8.23444
MB-4904	HER2+	7.625434
MB-4908	HER2+	7.544123
MB-4929	HER2+	8.68439
MB-4930	HER2+	7.775088
MB-4935	HER2+	7.480179
MB-4952	HER2+	7.414373
MB-5019	HER2+	7.305857
MB-5054	HER2+	8.061359
MB-5062	HER2+	7.335376
MB-5063	HER2+	7.38699
MB-5078	HER2+	7.418152
MB-5114	HER2+	7.569885
MB-5120	HER2+	7.609155
MB-5147	HER2+	7.713966
MB-5164	HER2+	7.570451
MB-5166	HER2+	7.5126
MB-5172	HER2+	7.954632
MB-5174	HER2+	7.072286
MB-5195	HER2+	7.529347
MB-5199	HER2+	7.765013
MB-5212	HER2+	7.788198
MB-5213	HER2+	7.381748
MB-5229	HER2+	7.493545
MB-5231	HER2+	7.732928
MB-5238	HER2+	7.186967
MB-5259	HER2+	7.667702
MB-5296	HER2+	8.079304
MB-5312	HER2+	7.492271
MB-5315	HER2+	7.453992
MB-5318	HER2+	7.631339
MB-5327	HER2+	8.101365

MB-5331	HER2+	8.28739
MB-5351	HER2+	7.073875
MB-5366	HER2+	8.055628
MB-5381	HER2+	7.74976
MB-5409	HER2+	7.587565
MB-5411	HER2+	7.567964
MB-5414	HER2+	7.685829
MB-5417	HER2+	7.815605
MB-5418	HER2+	7.805492
MB-5420	HER2+	7.622139
MB-5426	HER2+	8.467517
MB-5458	HER2+	7.480482
MB-5459	HER2+	8.961946
MB-5474	HER2+	7.784878
MB-5483	HER2+	8.100484
MB-5498	HER2+	8.581356
MB-5535	HER2+	7.858974
MB-5558	HER2+	8.001197
MB-5593	HER2+	7.537583
MB-6048	HER2+	7.531997
MB-6049	HER2+	7.612621
MB-6063	HER2+	7.586089
MB-6113	HER2+	7.453101
MB-6116	HER2+	7.589009
MB-6131	HER2+	8.34753
MB-6156	HER2+	7.675626
MB-6157	HER2+	8.098216
MB-6160	HER2+	7.941895
MB-6182	HER2+	7.368707
MB-6246	HER2+	7.772075
MB-6314	HER2+	8.350733
MB-6330	HER2+	7.802905
MB-6334	HER2+	7.578965
MB-6337	HER2+	7.749988
MB-6358	HER2+	7.768987
MB-6363	HER2+	7.922645
MB-7001	HER2+	6.976017
MB-7020	HER2+	7.559636
MB-7027	HER2+	7.65945
MB-7035	HER2+	7.722431
MB-7059	HER2+	7.309149

MB-7067	HER2+	7.946166
MB-7068	HER2+	7.068624
MB-7069	HER2+	7.670217
MB-7073	HER2+	7.596215
MB-7082	HER2+	6.90208
MB-7088	HER2+	7.101495
MB-7115	HER2+	7.256555
MB-7128	HER2+	6.672362
MB-7135	HER2+	7.043244
MB-7143	HER2+	6.986047
MB-7187	HER2+	6.817274
MB-7250	HER2+	7.049336
MB-7251	HER2+	7.323299
MB-7256	HER2+	7.747953
MB-7260	HER2+	7.684549
MB-7273	HER2+	6.733938
MB-7275	HER2+	7.246168
MB-7279	HER2+	6.351743
MB-7281	HER2+	7.272166
MB-7291	HER2+	7.174962

HER2+
7.536208
8.006231
7.523567
7.376333
7.574901
7.66911
7.52627
7.552488
7.93341
6.896603
7.416166
7.366319
7.457127
6.973352
7.572266
7.2746
7.349887
7.088382
7.562289
6.893906
7.038371
7.034683
6.951246
7.12062
7.646397
7.655764
7.083379
7.184102
7.651315
7.419283
7.163155
7.033896
7.1933
7.489127
7.30235
7.60961
7.83854
7.410124
7.081733
6.772255

7.498993
7.110394
8.208898
7.56283
7.660887
7.188777
7.031804
7.528534
7.080921
7.952145
6.969574
7.506739
7.392675
6.961581
8.095414
7.987515
7.375172
7.496384
7.592273
7.652058
7.266823
8.107543
7.57665
7.755374
7.777244
7.017244
7.471437
6.945318
7.621118
7.203484
7.522125
7.245854
7.727877
7.592395
8.445233
7.391911
7.531977
7.636601
7.995138
8.031167
7.298262

7.710432
7.504936
8.341324
7.715507
8.172286
8.380303
7.330933
8.001786
7.720726
8.331697
8.365581
7.970144
7.64541
8.199878
8.23444
7.625434
7.544123
8.68439
7.775088
7.480179
7.414373
7.305857
8.061359
7.335376
7.38699
7.418152
7.569885
7.609155
7.713966
7.570451
7.5126
7.954632
7.072286
7.529347
7.765013
7.788198
7.381748
7.493545
7.732928
7.186967
7.667702

8.079304
7.492271
7.453992
7.631339
8.101365
8.28739
7.073875
8.055628
7.74976
7.587565
7.567964
7.685829
7.815605
7.805492
7.622139
8.467517
7.480482
8.961946
7.784878
8.100484
8.581356
7.858974
8.001197
7.537583
7.531997
7.612621
7.586089
7.453101
7.589009
8.34753
7.675626
8.098216
7.941895
7.368707
7.772075
8.350733
7.802905
7.578965
7.749988
7.768987
7.922645

6.976017
7.559636
7.65945
7.722431
7.309149
7.946166
7.068624
7.670217
7.596215
6.90208
7.101495
7.256555
6.672362
7.043244
6.986047
6.817274
7.049336
7.323299
7.747953
7.684549
6.733938
7.246168
6.351743
7.272166
7.174962

Anova: Single Factor

SUMMARY

Tukey treatment group	Groups	Count	Sum	Average	Variance
A	ER-/HER2-	290	2178.52889	7.51216858	0.2353508
B	ER+/HER- Hig	603	4473.30201	7.41841129	0.18744252
C	ER+/HER2- L	619	4494.14065	7.26032415	0.13803239
D	HER2+	188	1422.25424	7.5651821	0.17094676

ANOVA

Source of Variati	*SS*	*df*	*MS*	*F*	*P-value*
Between Gro	20.97746	3	6.99248668	39.7791013	7.7028E-25
Within Grou	298.1278	1696	0.17578292		
Total	319.1053	1699			

treatments	Tukey HSD Q statistic	Tukey HSD p-value	inferfence
A vs B	4.4255	0.009639	** p<0.01
A vs C	11.9377	0.001005	** p<0.01
A vs D	1.9098	0.527305	insignificant
B vs C	9.3195	0.001005	** p<0.01
B vs D	5.9267	0.001005	** p<0.01
C vs D	12.3484	0.001005	** p<0.01

F crit
2.61014911
